# AirMeasurer: open‐source software to quantify static and dynamic traits derived from multiseason aerial phenotyping to empower genetic mapping studies in rice

**DOI:** 10.1111/nph.18314

**Published:** 2022-07-28

**Authors:** Gang Sun, Hengyun Lu, Yan Zhao, Jie Zhou, Robert Jackson, Yongchun Wang, Ling‐xiang Xu, Ahong Wang, Joshua Colmer, Eric Ober, Qiang Zhao, Bin Han, Ji Zhou

**Affiliations:** ^1^ State Key Laboratory of Crop Genetics & Germplasm Enhancement, Academy for Advanced Interdisciplinary Studies, Jiangsu Collaborative Innovation Center for Modern Crop Production Co‐sponsored by Province and Ministry Nanjing Agricultural University Nanjing 210095 China; ^2^ National Center for Gene Research, CAS Center for Excellence in Molecular Plant Sciences Chinese Academy of Sciences Shanghai 200233 China; ^3^ Cambridge Crop Research National Institute of Agricultural Botany (NIAB) Cambridge CB3 0LE UK; ^4^ Earlham Institute Norwich Research Park Norwich NR4 7UH UK

**Keywords:** 2D/3D trait analysis, aerial phenotyping, genetic mapping, predictive modelling, rice, static and dynamic traits

## Abstract

- Low‐altitude aerial imaging, an approach that can collect large‐scale plant imagery, has grown in popularity recently. Amongst many phenotyping approaches, unmanned aerial vehicles (UAVs) possess unique advantages as a consequence of their mobility, flexibility and affordability. Nevertheless, how to extract biologically relevant information effectively has remained challenging.
- Here, we present AirMeasurer, an open‐source and expandable platform that combines automated image analysis, machine learning and original algorithms to perform trait analysis using 2D/3D aerial imagery acquired by low‐cost UAVs in rice (*Oryza sativa*) trials.
- We applied the platform to study hundreds of rice landraces and recombinant inbred lines at two sites, from 2019 to 2021. A range of static and dynamic traits were quantified, including crop height, canopy coverage, vegetative indices and their growth rates. After verifying the reliability of AirMeasurer‐derived traits, we identified genetic variants associated with selected growth‐related traits using genome‐wide association study and quantitative trait loci mapping.
- We found that the AirMeasurer‐derived traits had led to reliable loci, some matched with published work, and others helped us to explore new candidate genes. Hence, we believe that our work demonstrates valuable advances in aerial phenotyping and automated 2D/3D trait analysis, providing high‐quality phenotypic information to empower genetic mapping for crop improvement.

## Abstract 2

See also the Commentary on this article by Yang & Zhai, **236**: 1229–1231.

## Introduction

Rice (*Oryza sativa*) is one of the key staple foods, feeding > 50% of the global population (Muthayya *et al*., 2014). Breeding for rice improvements in yield potential, grain quality and resistance to stresses is vital for its climate‐change adaptation and, thus, food security in many rice‐consuming nations (Nakashima *et al*., 2007; Jagadish *et al*., 2012). This relies on selecting favourable phenotypes of agronomic traits from thousands of varieties in the field, which in turn heavily relies on specialists' visual assessment (Bevan *et al*., 2017; Roitsch *et al*., 2019). To help accelerate the selection procedure, many field‐based phenotyping approaches have been introduced (Zhao *et al*., 2019; Yang *et al*., 2020).

Additionally, as agronomically important traits are controlled by the expression of multiple genes and modulated by the environment, phenotyping can assist researchers to understand underlying biological mechanisms that contribute to genetic gain (Hartung & Schiemann, 2014; Furbank *et al*., 2019). Through genome‐wide association studies (GWAS), the genetic architecture of some agronomic traits in rice has been dissected (Huang *et al*., 2010; Yang *et al*., 2014; Tang *et al*., 2019), laying the foundation of identifying functional diversity of alleles to discover valuable genes (Xing & Zhang, 2010). These contributions have led to advances in rice genetics and the development of new varieties with desired qualities, including high yield potential, resistance to stresses and increased resource‐use efficiency (Barabaschi *et al*., 2015; Du *et al*., 2018; Li *et al*., 2018).

Certain traits such as plant height can be phenotyped at a specific time point; however, for growth‐ and yield‐related traits that are genetically complex and influenced heavily by environmental factors, their phenotypes need to be examined dynamically (Naito *et al*., 2017; Mu *et al*., 2022). Nevertheless, to achieve this target, consistent data collection and trait analysis are required, which has posed significant challenges in developing reliable solutions for practical breeding programmes and field‐based plant research (Shakoor *et al*., 2017; Pieruschka & Schurr, 2019). In essence, several problems need to be addressed, including: (1) *scalability*, trials are normally large‐scale and at multiple sites; (2) *affordability*, resources are usually limited and solutions need to be cost‐effective; (3) *accuracy and repeatability*, analysis results should be consistent and reproducible in other trials; (4) *processing cycle*, the duration between breeding cycles or multiseason experiments is often brief, requiring data to be processed, analyzed and fed‐back promptly to enable timely decisions (Großkinsky *et al*., 2015; Atkinson *et al*., 2018). Recently, several advances have been adopted by breeders and plant researchers, but many attempts remain at early stages (White *et al*., 2012; Juliana *et al*., 2019). New tools derived from some academic research have often worked at relatively small scale and with limited accessibility as a result of bespoke hardware, proprietary software and specialized packages, preventing them from being employed easily (Yang *et al*., 2020, 2021). Furthermore, to exploit genomic resources, traits of interest and genetic diversity need to be assessed across sites and seasons, demanding accessible data collection and analysis toolkits (Naito *et al*., 2017; Atkinson *et al*., 2018). Hence, methodological advances shall intend to address the above challenges, which is the emphasis of this study.

One of the most exciting advances recently was the rapid development of unmanned aerial vehicles (UAVs, also known as unmanned aircraft systems) and their applications in crop monitoring resulting from their mobility, throughput and affordability (Shi *et al*., 2016; Maimaitijiang *et al*., 2017; Jang *et al*., 2020). There are numerous examples in the literature reporting UAV‐based phenotyping using sensors such as red‐green‐blue (RGB) cameras, multi‐ and hyperspectral devices, Light Detection and Ranging (LiDAR), and thermal and infrared sensors (Kachamba *et al*., 2016; Gracia‐Romero *et al*., 2017; Harkel *et al*., 2020; Hyyppä *et al*., 2020). Some work also reported quantitative trait loci (QTL) mapping of traits including plant height and vegetation fraction (Hassan *et al*., 2019; Wang *et al*., 2019; Ogawa *et al*., 2021). Nevertheless, most of these studies focused on estimating static traits collected at specific time points (Shakoor *et al*., 2017; Rodene *et al*., 2022), which often missed the dynamic nature of plant growth and development. Key agronomic traits (e.g. senescence and stem elongation) vary in time and space, which require new approaches to collect and analyse (Xu *et al*., 2018; Anderson *et al*., 2020). In fact, in a trial containing diverse genotypes, each line grows at a different pace, and thus dynamic analysis can provide meaningful comparisons between the genotypes (Hartung & Schiemann, 2014). Finally, changing behaviours of target traits, within or across seasons, can characterize the plant's complex responses to external stimuli, which are direct evidence to reveal spatial and temporal changes in the expression of genes and their regulators (Roitsch *et al*., 2019; Mu *et al*., 2022).

To extract meaningful information from UAV‐collected imagery, many analytic solutions have been developed to measure traits related to yield, stress tolerance and growth patterns, using morphological, spectral and textural properties (Perez‐Sanz *et al*., 2017; Jiang *et al*., 2021), most of which have focused on dryland crops. For example, Easy MPE (Tresch *et al*., 2019) combined excess green (ExG) and automatic thresholding to study soybean; AirSurf (Bauer *et al*., 2019) employed deep learning to count and classify lettuces; Grid (Chen & Zhang, 2020; Tang *et al*., 2021) utilized pixel‐wise K‐means clustering to delineate irregular (e.g. zigzag) or regular (e.g. grid‐based) trial layouts for wheat trials; R/UAS::plotshpcreate (Anderson & Murray, 2020) created polygon shapefiles using parameters (e.g. field direction and plot size) to study maize; FIELDimageR (Matias *et al*., 2020) incorporates manual inputs (e.g. row and column numbers) into the extraction of plot‐based traits for potato.

Still, limited tools are available for nonexperts to examine multigenic traits and develop markers for paddy field crops (e.g. rice), which are complex as a consequence of changing water levels (e.g. resulting from rainfall and draining) and many voluntary plants (e.g. duckweed) compared with dryland crops (Ogawa *et al*., 2021). Moreover, few research groups have the resources to process large‐scale aerial images, or to develop complex algorithms to address problems in automated trait analysis (Roitsch *et al*., 2019; Zhu *et al*., 2021). Hence, along with the development of open‐source computer vision, machine learning and data science libraries (Howse, 2013; Virtanen *et al*., 2020), open solutions will be valuable to equip plant researchers with new toolkits to study complicated crops.

In order to address some of the challenges, we have developed AirMeasurer, an open‐source platform that automates trait analysis for rice trials using 2D orthomosaics and 3D point clouds acquired by low‐cost UAVs. First, we established tailored protocols for regular flight missions and data pre‐processing. Secondly, varied 2D/3D analysis algorithms were integrated into the platform to quantify static traits such as seedling number, plant height, canopy coverage and vegetative indices, using morphological, spectral and textural signals. Thirdly, we developed an original algorithm to compute dynamic traits based on static traits, including growth rates of the target traits and their rapid growth phases, which were time‐consuming or impossible to score previously. To ensure that our work could reach the broader research community, we created a graphical user interface (GUI) for nonexperts to use. Finally, to validate the platform and its utility in research, we applied the AirMeasurer‐derived traits collected from hundreds of rice landraces and recombinant inbred lines (RILs) in a multiseason case study (2019–2021) to genetic mapping studies (i.e. GWAS and QTL mapping) and identified reliable loci.

## Materials and Methods

### Plant materials and field experiments

In order to develop a UAV‐based imaging protocol for multisite phenotyping, we established two experiments (2019–2021; Supporting Information Fig. S1a): (1) one focused on 254 landraces (Huang *et al*., 2012) in Shanghai (the 2019/2020 seasons), including 103 *japonica*, 40 intermedia and 111 *indica* types; (2) the other studied 191 homozygous RILs in Hainan (the 2020/2021 seasons), derived from the crossing parents Nipponbare (*Oryza sativa* ssp. *Japonica*) and 93–11 (*Oryza sativa* ssp. *Indica*), two popular varieties (Huang *et al*., 2010). In 2019, 177 RILs were used for manual assessment as a consequence of agronomic issues with some RILs during grain‐filling. The sites were chosen owing to their geography and weather conditions. Crops at both sites were managed using standard husbandry and agronomic inputs according to local conditions. Landraces (Fig. S1b) and RILs (Fig. S1c) were sown in 2 × 1.1 m plots, 18 plants per plot. To maximize the efficient use of field space and facilitate initial selection (Payne, 2006), we did not introduce plot‐level replicates; however, the same lines were repeatedly used in this multiseason case study. Details of the trial design, plant materials and geo‐locations are provided in Notes S1.

### Ground truthing

In‐field ground truth measurements to validate AirMeasurer‐derived traits were conducted by field workers. Maximum plant height was measured with a metre ruler in the late reproductive phase. After grain‐filling, six plants in a plot were straightened and the distance from ground level to the top of rice spikes was measured. Heading date was scored manually, when there were five plants with panicles emerging 25 mm above the flag leaf sheath. To verify traits such as ExG and canopy coverage used for dynamic trait analysis, images of 29–30 randomly selected plots at six growth stages between early vegetation and early ripening (177 plots in total) were analyzed manually using the Fiji/ImageJ software (Schindelin *et al*., 2012), through which plot‐based green‐channel intensity values (0–255; measured from linear histogram) and canopy coverage (in pixels; using the auto‐thresholding function) were obtained. To validate AirMeasurer‐derived plant height at different growth stages, technicians manually measured calibrated 3D point clouds (with unwanted terrain features removed) to obtain plot‐level canopy height at eight time points throughout the 2019 season (177 per point, 1416 in total).

### Workflow of UAV‐based phenotyping

**Fig. 1:**
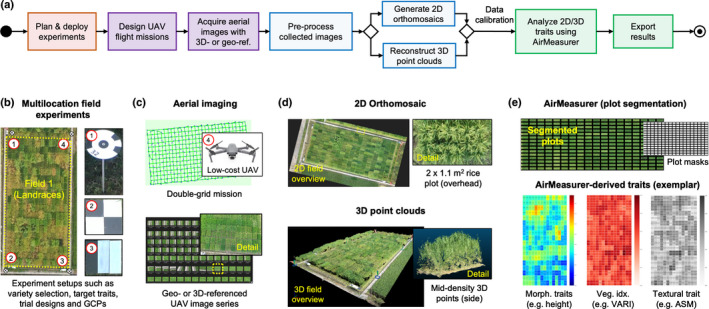
A general workflow of unmanned aerial vehicle (UAV) based field phenotyping and phenotypic analysis established for collecting 2D/3D aerial images, processing 3D point clouds, and measuring plot‐based morphological, spectral and textural traits. (a) A high‐level workflow established to perform UAV‐based field phenotyping and phenotypic analysis at multiple sites and over the course of multiple seasons. (b) Field experiments designed based on biological questions concerning plant varieties, target traits, treatments, trial layouts and in‐field setups (e.g. ground control points, GCPs). (c) The selection of imaging protocols to collect aerial image series with 3D‐ or geo‐referencing information. (d) Data pre‐processing to produce 2D orthomosaics and 3D point clouds for the experimental field with plot‐level plant resolution. (e) Automated trait analysis using a combination of 2D/3D image processing, spectral analysis, and machine learning techniques to perform plot segmentation and plot‐based trait analysis using morphological, spectral, and textural signals (all the traits produced by AirMeasurer are listed in Table 1).

When carrying out aerial phenotyping, we implemented a four‐step workflow (Fig. 1a): (1) *experiment setups* – including trial design (e.g. field layouts, target traits) and ground control points (GCPs; Figs 1b, S1d); (2) *aerial imaging* – providing guidelines to pilots to execute flight plans (Figs 1c, S1e); (3) *data pre‐processing* – producing 2D field‐level orthomosaic images (in TIFF) and 3D point cloud files (in LAS) from acquired aerial images using the pix4dmapper software (Pix4D, Lausanne, Switzerland; Fig. 1d); and (4) *phenotypic analysis* – combining spectral, textural and morphological properties of plants to perform automated trait analysis (Fig. 1e).

### Aerial imaging using low‐cost UAVs

At each site, in‐field settings (Fig. 1b) such as GCPs, height reference panels, spectral reflectance mats, or real‐time kinematic positioning (RTK) were applied according to recommended practices published previously (Watanabe *et al*., 2017). To ensure that the imaging protocol could be adopted easily, we chose to use low‐cost drones (e.g. Mavic 2 Pro; DJI, Shenzhen, China). Because smaller UAVs generated less downdraft, they could limit wind disruption of plant canopies during the low‐altitude imaging. We designed two mission plans: (1) field‐level imaging (25–35 m altitude), collecting RGB images speedily to limit colour distortion caused by natural illuminance (e.g. Fig. 1b left); (2) plot‐level imaging, conducting flights with tailored flight parameters at low altitudes (10–15 m). Flights were normally carried out 10–12 times per season, among which eight flights were selected for time series measures (detailed mission plans, imaging protocols and guidelines are included in Notes S2).

### 3D point cloud processing and canopy height model

**Fig. 2:**
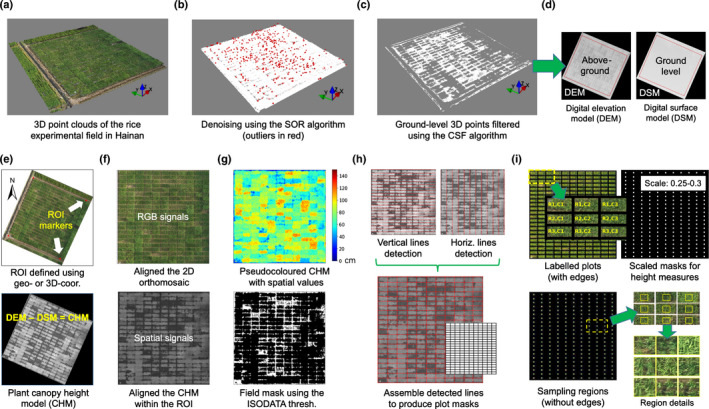
Algorithmic steps for processing unmanned aerial vehicle (UAV) collected 3D point clouds to generate aligned canopy height model (CHM) within region of interest (ROI) together with plot segmentation for plot‐based trait analysis. (a) A 3D point cloud file produced from pre‐processing (in LAS format). (b) Outliers (red) removed in the point clouds using the Statistical Outlier Removal (SOR) algorithm. (c, d) The Cloth Simulation Filter (CSF) algorithm applied to differentiate ground‐level and aboveground 3D points, resulting in a digital elevation model (DEM) and a digital surface model (DSM). (e) Region of interest (ROI), denoted by four red markers recorded from ground control points (GCPs) with 3D‐ or geo‐coordinates; then, DSM subtracted from DEM to generate a canopy height model (CHM), which uses greyscale values (0–255) to present plant height values. (f) A 2D perspective transformation applied to produce aligned red‐green‐blue (RGB) and CHM images using the ROI markers. (g) Pseudocolour applied to the aligned CHM according to a unified height scale bar (0–150+ cm; right); then, the iterative self‐organizing data (ISODATA) thresholding algorithm employed to produce a field‐level mask from the CHM. (h) The Hough transform algorithm used to detect horizontal and vertical lines separately, followed by the assembly of detected lines to produce initial plot masks. (i) All of the plots labelled based on the trial design; then, the scaling function applied to remove edge effects and overlapping plants among neighbouring plots, resulting in refined sampling regions for height (scale = 0.25–0.3) and colour‐related measures in all the plots.

There can be unwanted noise in 3D point clouds generated by the Structure‐from‐Motion (SfM) algorithm (Singh & Frazier, 2018). To measure morphological features reliably (Fig. 2a), we first denoised the SfM‐generated 3D points (e.g. for a 0.1‐ha field, low‐density 3D reconstruction could produce > 30 million points). Second, we implemented the Statistical Outlier Removal (SOR) algorithm (Hodge & Austin, 2004) to remove outliers (red‐coloured points, Fig. 2b). Third, a ground‐level filter was developed based on the Cloth Simulation Filter (CSF) algorithm (Zhang *et al*., 2016), classifying denoised 3D points into ground‐level and aboveground groups. Because the CSF was designed for ultra‐large land surveillance, we optimized the filter by reducing its grids and nodes (Fig. 2c). Finally, we removed unwanted terrain features (e.g. the field‐level slope) using geo‐ or 3D‐coordinates recorded from GCPs (saved in a shapefile, SHP). The procedure to correct geometric distortion is included in Notes S2 and S3, which shows the improved height measurements after removing field‐level slopes.

Next, we generated a digital surface model (DSM, i.e. ground‐level points) and a digital elevation model (DEM, i.e. aboveground points) using the *LidarTinGridding* function (Lindsay, 2016) in whiteboxtools (Fig. 2d). We defined region of interest (ROI) according to the SHP file (red markers, Fig. 2e). The DSM was subtracted from the DEM to retain aboveground plant information, resulting in a canopy height model (CHM) representing plant spatial signals with greyscale values (i.e. the brighter a pixel, the higher the point). Finally, we combined the CHM with spectral signals using the *getPerspectiveTransform* function (Mezirow, 1978) in openCV, which realigned the CHM (Fig. 2f, upper) with the field‐level orthomosaic (Fig. 2f, lower). Spatial features were pseudocoloured (Fig. 2g, upper), ranging from 0 (dark blue, for bare plots) to 150+ cm (dark red, for tall plants). Python‐based software implementation for the above algorithms is given in Notes S4.

### Plot segmentation

In order to acquire plot‐based trait information routinely, plant plots should be identified consistently. Recent solutions such as Easy MPE, GRID, AirSurf, R/UAS::plotshpcreate and FIELDimageR have been applied to segment plots or plant blocks for species such as soybean, wheat and maize, which were valuable advances for dryland crops. We trialled them in our paddy rice experiments and encountered segmentation issues as a consequence of unclear plot boundaries, changing water levels and overlapped rice plants during ripening (Notes S5).

Consequently, we developed an optimized plot segmentation algorithm (Notes S6): (1) applied an iterative self‐organizing data thresholding (Irvin *et al*., 1997) to a field‐level CHM and generated a global mask to represent plot edges (Fig. 2g, lower); (2) the Hough transform (Duda *et al*., 1972) was employed to seek horizontal and vertical lines in the mask, respectively (Fig. 2h, upper); (3) when some boundaries were undetectable, vertical and horizontal lines could be drawn manually to improve plot delineation via the GUI; (4) as most of the plots were not distanced evenly even with RTK‐assisted seed drilling, we merged the adjacent lines; (5) after detecting plot boundaries, we assembled the remained lines to generate plot masks (Fig. 2h, lower), based on which all the plots were labelled according to the trial design for indexing and cross‐referencing purposes (Fig. 2i, upper left); (6) to minimize edge effects and remove overlapped plants between neighbouring plots, a scaling function was designed to rescale the plot masks to measure different traits (e.g. scale = 0.25–0.3 for height measurements, depending on the degree of plant overlapping; Fig. 2i, upper right); and (7) finally, the refined masks were used to generate plot‐level sampling regions (Fig. 2i, lower).

### Automated trait analysis

**Fig. 3:**
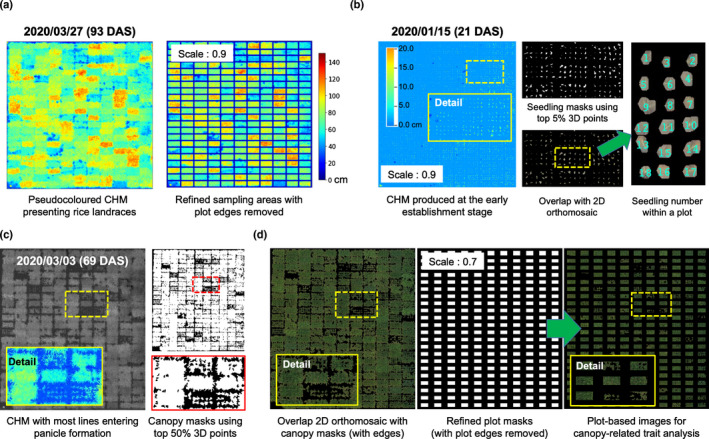
Algorithmic steps for quantifying plot‐based morphological traits such as rice seedling number and canopy‐related traits using both spatial and spectral signals. (a) Plot masks rescaled (scale = 0.9) to segment a canopy height model (CHM) image collected at 93 d after sowing (DAS); the segmented CHM (right) pseudocoloured according to the unified height scale bar (0–150+ cm). (b) The rescaled plot masks applied to divide a field‐level CHM acquired at early establishment (21 DAS) with a new height scale bar (0–20 cm; left), displaying height differences for short rice seedlings. Top 5% of 3D points (*H*_95th_) in a plot utilized to produce a plot‐based seedling mask, followed by overlapping the mask with 2D orthomosaic (collected at 21 DAS; middle); finally, excess green index (ExG) computed to remove nonseedling objects, resulting in the quantification of seedling number per plot (right). (c) A field‐level CHM (69 DAS) used to compute canopy coverage index (CCI; left); top 10% of 3D points (*H*_90th_) in a plot used to create plot‐based canopy masks (right). (d) After overlapping the canopy masks with 2D orthomosaic (69 DAS; left), edges of the canopy removed using the scaling function (scale = 0.7; middle), resulting in refined plot‐based canopy regions for computing canopy coverage and canopy ExG indices.

Rice growth and development can be associated with stem elongation (i.e. changes in height) over time (Hosoi & Omasa, 2012). We utilized both spatial and spectral signals to analyze growth‐related traits. For different morphological traits, varied vertical levels of spatial signals were used. For example, we chose the top 10% height values (*H*_90th_, i.e. top 10% of the 3D points; see reasoning in Notes S7) in the CHMs (scale = 0.25–0.3) to compute canopy plant height after grain‐filling. For early establishment, as colour or textural signals were unreliable in identifying seedlings owing to weedy plants and changing water levels, we therefore first segment plant signals from CHMs (scale = 0.9; Fig. 3a); then, seedling masks were generated using *H*_95th_ as a result of short seedlings (Fig. 3b, left); after removing noisy objects (e.g. nongreen pixels) based on ExG values, we separated seedling objects from their surrounding pixels using morphological erosion (Fig. 3b, middle), followed by indexing seedlings (Fig. 3b, right; Fig. S2).

Comparably, we applied similar steps to measure canopy coverage before canopy closure: (1) using *H*_50th_ to represent plot canopy as canopy density was low during early vegetative phase (Fig. 3c, left); (2) applying the local adaptive thresholding (Singh *et al*., 2012) to generate a field‐level mask (Fig. 3c, right); (3) overlapping the mask with 2D orthomosaic (Fig. 3d, left); (4) removing plot edges as some gaps between plots were unclear at canopy closure (scale = 0.7; Fig. 3d, middle); (5) using the Lab colour space (McLaren, 1976) to filter nongreen pixels (Fig. 3d, right); and (6) computing normalized canopy coverage index (CCI; 0 to 1, where 1 stands for 100% coverage; Notes S8).

According to a recent report (Svensgaard *et al*., 2021), RGB sensors can be applied to perform reliable spectral analysis without radiation calibration. Hence, we used RGB sensor to compute growth‐related vegetation indices in the study. A series of vegetative indices and textural traits (e.g. canopy uniformity) were produced (Notes S9). All of the traits produced by AirMeasurer are listed in Table 1.

**Table 1:** All of the traits that can be produced by the AirMeasurer platform (their equations, normalization, references and biological relevance are provided in Supporting Information Notes S9).

Signals	Name (plot‐based static traits)	Dynamic traits (based on static traits)
Morphological traits	1. Canopy plant height^a^ (all growth stages)	18. Rapid growth phase^b^ (RGP), for traits measured from 0 DAS to ripening.
	2. Canopy coverage index^a^ (CCI), from 0 d after sowing (DAS) to canopy closure	
	3. Seedling number^b^ (early establishment)	
	4. 3D canopy index^b^ (3DCI, stem elongation – ripening)	
	5. 3D leaf area index^b^ (tillering – ripening)	
Spectral traits	6. Excess green^a^ (ExG; all stages)	19. Fastest growth rate^b^ (FGR), within the RGP of traits measured.
	7. Excess red^a^ (ExR; all stages)	
	8. Normalised vegetative index^a^ (NVI; all stages)	
	9. Green leaf index^a^ (GLI; all stages)	
	10. Visible atmospherically resistant index^a^ (VARI; all stages)	
	11. Normalized difference yellowness index^a^ (NDYI; all stages)	
Textural traits	12. Greyscale co‐occurrence matrices^a^ (GLCMs; stem elongation – ripening)	20. Average growth rate^b^ (AGR), the duration is changeable (e.g. 0 DAS – the ${\text{Max}}_{\text{height}}$ day, 0 DAS – the FGR day, or within the RGP).
	13. Angular second moment^a^ (ASM, canopy uniformity; canopy closure – ripening)	
	14. GLCM‐based canopy dissimilarity^a^ (canopy closure – ripening)	
Model‐predicted traits (based on static & dynamic traits)	15. Heading date^b^ (predicted using supervised machine learning techniques)	
	16. The ${\text{Max}}_{\text{trait}}$ day^b^ (estimated using the dynamic trait analysis)	
	17. The beginning of ripening^b^ (estimated using the dynamic trait analysis)	

^a^ Integrated in the GUI software.

^b^ Executed via modules or trained models through Jupyter notebooks or Python scripts.

### Analysis of dynamic traits

**Fig. 4:**
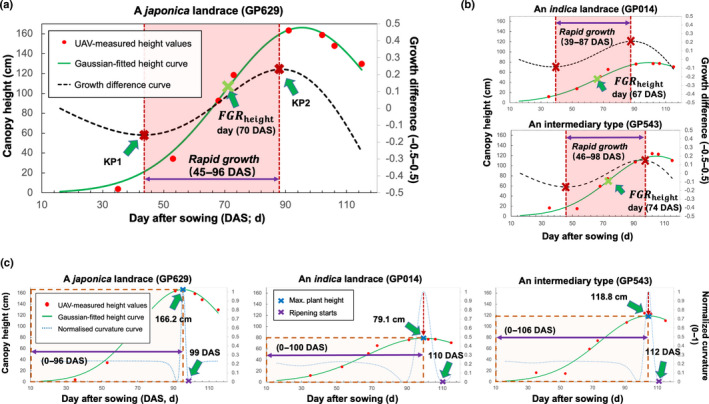
Algorithmic steps for quantifying dynamic phenotypes of an example trait, canopy height growth rate; three types of rice landraces are shown to illustrate the procedure and the capability of estimating growth‐related traits. (a) Eight canopy height values (red dots) recorded between sowing and grain‐filling for a given *japonica* landrace, which were relatively evenly distanced during key growth stages, between 10 and 115 d after sowing (DAS). The Gaussian function applied to produce a growth curve of canopy height ($f{\left(x\right)}_{\text{height}}$; green colour), based on which a growth‐difference curve ($f{\left(x\right)}_{\text{diff}}$; black dash curve) was created. $f{\left(x\right)}_{\text{diff}}$ measures value changes on $f{\left(x\right)}_{\text{height}}$, indicating the rate of canopy height change during the season. Turning points (i.e. knee points, KPs; red crosses) on $f{\left(x\right)}_{\text{diff}}$ located to represent the rapid growth phase of canopy height (${\mathrm{RGP}}_{\text{height}}$; in days; red shading area), indicating the most rapid period of stem elongation. Within the ${\mathrm{RGP}}_{\text{height}}$, the fastest growth rate (${\text{FGR}}_{\text{height}}$; the light‐green cross) located by computing the first derivative of $f{\left(x\right)}_{\text{height}}$ within the ${\text{RGP}}_{\text{height}}$ period. (b) The same algorithmic steps followed to analyze dynamic phenotypes for two *indica* and *intermediate* landraces. (c) The maximum canopy height (${\mathrm{Max}}_{\text{height}}$; in cm), its associated DAS, and key growth stages such as the beginning of ripening estimated using maximum and minimum curvature values on a normalized‐curvature curve $f{\left(x\right)}_{\mathrm{cuv}}$ (dotted blue) derived from the $f{\left(x\right)}_{\text{height}}$ for three types of rice landraces.

Because dynamic or longitudinal phenotypes can be more informative in revealing plant–environment interactions (Campbell *et al*., 2019), we derived dynamic traits from static traits collected at different growth stages, rather than using values scored at arbitrary time points to represent growth patterns. Inspired by previous research (Anderson *et al*., 2019), we chose to measure dynamic phenotypes from the fitted curves even if some phenotyping points might be missing. The following section describes steps to compute dynamic phenotypes for an example trait, canopy height growth:

1. Eight height values were used between sowing and grain‐filling for a given *japonica* landrace (red dots in Fig. 4a). The eight points were relatively evenly distanced between 10 and 115 d after sowing (DAS). Because the height of rice canopy tends to decrease during the later grain‐filling period, we tested several fitting functions (e.g. stepwise regression) and chose the Gaussian function to fit plant height changes (green curve, Fig. 4a).
2. The Gaussian‐fitted height curve $f{\left(x\right)}_{\text{height}}$ then was used to generate a growth‐difference curve $f{\left(x\right)}_{\text{diff}}$ (black dash curve, Fig. 4a) through the *KneedLocator* function (Satopää *et al*., 2011), which measures value changes on $f{\left(x\right)}_{\text{height}}$, signifying the rate of plant height changes (i.e. increasing, decreasing or constant).
3. Turning points (i.e. knee points, KPs; red crosses, Fig. 4a) were located on $f{\left(x\right)}_{\text{diff}}$, indicating height change phases. To locate theses KPs, we found the first ($f^{\prime }{\left(x\right)}_{\text{diff}}$) and second ($f\prime \prime {\left(x\right)}_{\text{diff}}$) derivatives on $f{\left(x\right)}_{\text{diff}}$; KP1 was detected when $f^{\prime }{\left(x\right)}_{\text{diff}}$ = 0 and $f\prime \prime {\left(x\right)}_{\text{diff}}$ > 0, whereas KP2 was detected when $f^{\prime }{\left(x\right)}_{\text{diff}}$ = 0 and $f\prime \prime {\left(x\right)}_{\text{diff}}$ < 0. We named the phase between KP1 and KP2 as rapid height growth phase (${\mathrm{RGP}}_{\text{height}}$; in days), denoting the period of rapid stem elongation.
4. Within the ${\mathrm{RGP}}_{\text{height}}$, we found the first derivative $f^{\prime }{\left(x\right)}_{\text{height}}$ (green curve) to locate the day when canopy height was changing at the fastest growth rate (i.e. the ${\mathrm{FGR}}_{\text{height}}$ day, in DAS; light‐green cross; Fig. 4a) together with computing average growth rate (${\mathrm{AGR}}_{\text{height}}$; %), between 0 DAS and the ${\mathrm{FGR}}_{\text{height}}$ day.

Then, we applied the above steps to analyze *indica* and *intermediate* landraces (e.g. GP014 and GP543; Fig. 4b). ${\mathrm{RGP}}_{\text{height}}$ values for the genotypes were identified together with the ${\mathrm{FGR}}_{\text{height}}$ days and ${\text{AGR}}_{\text{height}}$. To assess phenotypic changes for other growth‐related traits, we employed the algorithm to study variables such as ExG (i.e. ${\text{RGP}}_{\text{ExG}}$, ${\text{FGR}}_{\text{ExG}}$ and ${\text{AGR}}_{\text{ExG}}$) and CCI (i.e. ${\text{RGP}}_{\text{CCI}}$, ${\text{FGR}}_{\text{CCI}}$ and ${\text{AGR}}_{\text{CCI}}$). We also applied $f{\left(x\right)}_{\text{height}}$ (green curves, Fig. 4c) to estimate ${\text{Max}}_{\text{height}}$ and other key growth stages (e.g. the beginning of ripening) using a normalized‐curvature curve $f{\left(x\right)}_{\mathrm{cuv}}$ (dotted blue, Fig. 4c). The maximum curvature on $f{\left(x\right)}_{\mathrm{cuv}}$ was located to represent the ${\text{Max}}_{\text{height}}$ day (blue crosses, Fig. 4c), followed by the estimation of the beginning of ripening (purple crosses, Fig. 4c) using the minimum curvature. Moreover, ${\text{AGR}}_{\text{height}}$, ${\text{AGR}}_{\text{ExG}}$ and ${\text{AGR}}_{\text{CCI}}$ (all in %) between 0 DAS and the ${\text{Max}}_{\text{height}}$ day also were quantified. To compute $f{\left(x\right)}_{\mathrm{cuv}}$, we used the equation below:

(Eqn 1) $$K=\frac{|f\prime \prime {\left(x_{i}\right)}_{\text{height}}|}{{\left(1+{\left(f^{\prime }{\left(x_{i}\right)}_{\text{height}}\right)}^{2}\right)}^{\frac{3}{2}}}$$

($f{\left(x_{i}\right)}_{\text{height}}$, Gaussian‐fitted height curve; *i* is between 10 and 115 DAS).

### Python‐based software implementation

A relative lack of open analytic solutions impedes researchers from exploiting newly introduced methods. Hence, we chose to develop the airmeasurer GUI using Python programming language together with a modular design, so that each function or module in the platform could be accessed and modified independently. We used the Tkinter toolkit (Shipman, 2013) to develop a cross‐platform GUI (in EXE). Open‐source libraries such as SciPy, OpenCV and Scikit‐Learn were employed to develop 2D/3D trait analysis algorithms and machine‐learning based predictive modelling. The GUI software, executable Jupyter notebooks, and user guides are provided for academic use (*Availability and Requirements*).

### GWAS analysis and QTL mapping

The AirMeasurer‐measured and manual‐scored traits collected from landraces were used to perform GWAS analysis to find the associated‐loci controlling phenotypes. The RIL population was used to verify the static and dynamic traits through QTL mapping. For GWAS analysis, an efficient mixed‐model association eXpedited (EMMAx) was performed (Kang *et al*., 2010). Single‐nucleotide polymorphisms (SNPs) with minor allele frequency (MAF) < 0.05 were excluded. Lines with missing phenotypes as a result of agronomic reasons were excluded. In total 254 landraces (2019/2020 seasons) were used to conduct GWAS. The matrix of pairwise genetic distances obtained by the simple SNP matching coefficients was employed to model the variance–covariance matrix of the random effect. Permutation tests were applied to help define the threshold of association signals (Churchill & Doerge, 1994). For each trait, we reshuffled the phenotypic data and performed association analysis using EMMAx with same parameters. To determine the significant threshold in GWAS, we accomplished 100 permutation analyses for each trait. Manhattan and quantile‐quantile (QQ) plots were produced by using the Perl scripts (Mägi & Morris, 2010). For the 191 homozygous RILs (2020/2021 seasons), sequencing and genotyping were conducted using the published pipeline and SEG‐map (Zhao *et al*., 2010). Windows QTL cartographer v.2.5 (Wang, 2007) was employed for QTL analysis of composite interval mapping. LOD value was computed to indicate the possibility of QTLs based on likelihood ratio tests.

## Results

### Collected 2D/3D aerial images

Using low‐cost UAVs to monitor rice experiments between 2019 and 2021, many series of 2D/3D aerial images were generated. For example, eight flights conducted in the 0.1‐ha trial in Shanghai generated 10 GB 2D/3D imagery from over 100 GB raw images in a season. For the 0.2‐ha trial in Hainan, 13 GB 2D/3D imagery was created from eight flights (145 GB raw images). We uploaded a series of testing files to our GitHub repository (10 GB in total) for researchers to test and improve AirMeasurer.

### The GUI software

**Fig. 5:**
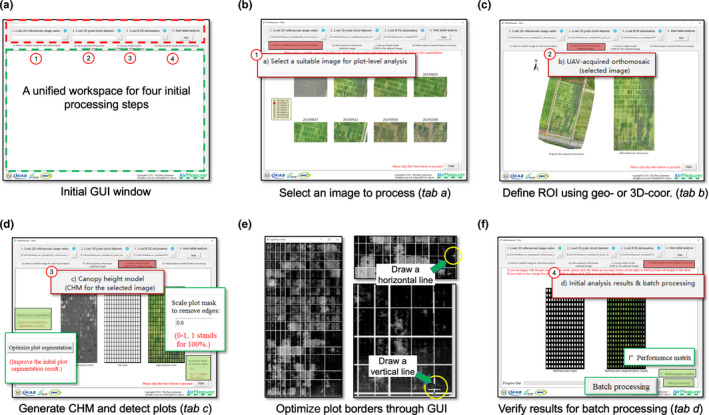
Graphic user interface (GUI) of airmeasurer developed for nonexpert users to readily use, which is capable of batch processing a series of 2D orthomosaics and 3D point clouds for 2D/3D trait analysis. (a) Initial GUI window of airmeasurer, consisting of input and analysis sections. A series of 2D orthomosaics, 3D point clouds, and 3D‐ or geo‐coordinates (in SHP format) could be selected in the input section to initiate the initial analysis. (b) ‘tab a’ used to select an image with relatively clear gaps between plots from a list of input 2D orthomosaics. (c) ‘tab b’ used to define region of interest (ROI) of the field experiment using 3D‐ or geo‐coordinates. (d and e) ‘tab c’ used to generate a field‐level plant canopy height model (CHM) and plot masks. If the generated masks failed to delineate all the plot boundaries, an ‘Optimize plot segmentation’ button (coloured green) could be used to draw horizontal or vertical lines using a mouse (yellow circles); also, the ‘Scale plot mask’ input box could be used to scale down the plot masks (0–1, where 1 stands for 100% of the original mask), removing plot edges and overlapping plants. (f) ‘tab d’ used to visualise pre‐processing results, a ‘Batch processing’ button to initiate automated trait analysis together with a progress bar and a checkbox for generating a performance matrix for all the rice genotypes. After the batch processing, trait analysis results (in comma‐separated values, CSV), plot‐based red‐green‐blue and CHM images (in JPG format) could be downloaded via the GUI. GUI‐produced traits are listed in Table 1.

The initial GUI window of airmeasurer software consists of an input (red dash rectangle) section and a unified workspace (green dash rectangle; Fig. 5a). Users can select 2D/3D image series and a SHP file to begin the processing, including: (1) ‘tab a’ shows the central portion of the input orthomosaics within several seconds so that users can choose one image to proceed (Fig. 5b); (2) ‘tab b’ defines ROI and aligns the selected orthomosaic (Fig. 5c); (3) ‘tab c’ generates a CHM using the associated 3D point clouds and performs initial plot segmentation (Fig. 5d); (4) if some plot boundaries fail to delineate, users can use a mouse to draw horizontal and vertical lines to improve the plot delineation (yellow circles; Fig. 5e); (5) users can refine the plot masks using the scale function (0–1, where 1 stands for 100% of the original masks; Fig. 5d right); and (6) ‘tab d’ visualizes initial results and a button for batch processing with a progress bar and a checkbox for generating a performance matrix for genotypes (Fig. 5f). A different image can be reselected in ‘tab a’ to repeat the above procedure. The initial plot masks will be used to benchmark all the input images during batch processing. Finally, plot‐based trait analysis and processed plot‐level images can be downloaded. Using an ordinary computer (Intel Core i7 CPU, 16 GB RAM with integrated graphics), 16 2D/3D images (10 GB) took 3 h to process. A detailed step‐by‐step user guide is provided in Notes S10 and Video S1.

### Multiseason plant height analysis

**Fig. 6:**
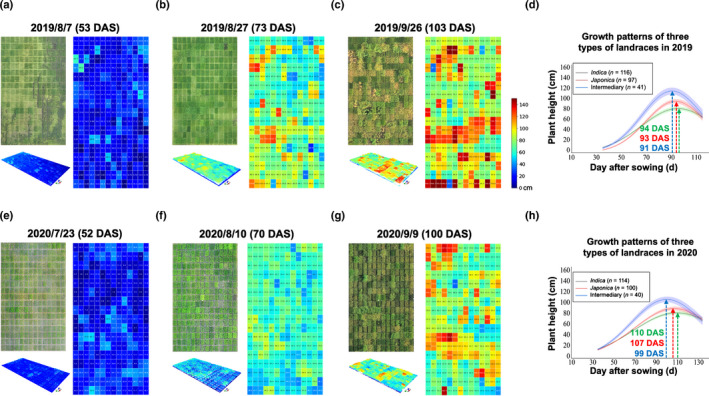
A series of 2D orthomosaics, pseudocoloured height maps and 3D point clouds collected by low‐cost unmanned aerial vehicles (UAVs) in the 2019 and 2020 seasons from 254 rice landraces in Shanghai. (a–c) 3D point clouds (from a 60° perspective) and overhead 2D orthomosaic of 254 landraces generated from a series of UAV phenotyping conducted over the 2019 season in Shanghai (to the left). Pseudocoloured height maps (to the right), showing plot‐based canopy plant height values for all the plots in the field. (d) Quantification of growth curves using AirMeasurer‐measured canopy height values for three types of rice landraces (i.e. *indica*, *japonica* and intermediary) over the 2019 season. Coloured shading areas denote confidence intervals (15^th^–85^th^ percentiles). The three coloured dashed arrows indicate when the average maximum height values of the three types of landraces were reached (in days after sowing, DAS). (e–h) Experiments of the same 254 landraces repeated in the 2020 season, producing 3D point clouds, 2D orthomosaics, the height maps and derived growth curves. The unified height scale bar (0–150+ cm) for the subfigures is shown.

We applied the AirMeasurer system to process flights conducted in multiseason rice trials. For visual display, we selected three 2D orthomosaics to present overhead imagery and three 3D point clouds to exhibit field‐level plant spatial features, from a 30° perspective, on 53, 73 and 103 DAS in the 2019 season, when landraces entered vegetative, reproductive and ripening phases (Fig. 6a–c, left). Pseudocoloured height maps (Fig. 6a–c, right; with a unified scale bar) were created using AirMeasurer‐derived height measures, showing height changes of the 254 landraces during the season. We applied the Gaussian‐fitted curves and categorised the landraces into three groups according to their domestic types (i.e. *indica*, *japonica* and intermediary), with coloured shading areas denoting 15^th^–85^th^ percentile confidence intervals (Fig. 6d).

The fitted curves roughly followed a sigmoid pattern but with dissimilar developmental rates. For example, the intermediary group peaked on 91 DAS ($\bar{{\text{Max}}_{\text{height}}}$ = 1.18 m), followed by the *japonica* group (93 DAS; $\bar{{\text{Max}}_{\text{height}}}$ = 0.97 m) and the *indica* group (94 DAS; $\bar{{\text{Max}}_{\text{height}}}$ = 0.82 m). For the 2020 height measures, three flights (on 52, 70 and 100 DAS) were selected to visualize plot‐based height differences (Fig. 6e–g). The Gaussian‐fitted curves identified similar growth patterns (Fig. 6h); for example, the $\bar{{\text{Max}}_{\text{height}}}$ days are between 95 and 110 DAS, and the 2020 lines were 5–10 cm shorter than the same lines studied in 2019. Complete height measurements for the two seasons (Datasets S1, S2), 2D orthomosaics and pseudocoloured height maps for the 191 RILs are provided (Fig. S3).

### Performance matrix and dynamic trait analysis

**Fig. 7:**
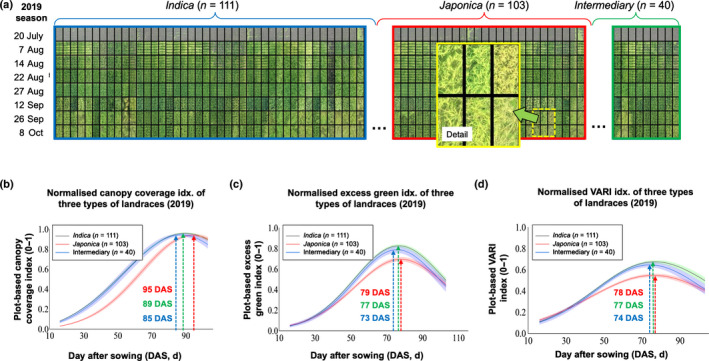
Matrix generated to provide a comprehensive overview of the performance of 254 rice landraces in the 2019 season, through which dynamic analysis of normalized canopy coverage index (CCI), excess green (ExG) and visible atmospherically resistant index (VARI) were performed. (a) Eight 2D orthomosaics collected between 20 July and 8 October 2019 used to generate the performance matrix, where each cell was an example canopy image of a rice genotype, such that genotypes were columns and UAV phenotyping timepoints were rows. In the performance matrix, the 254 rice landraces rearranged according to three domestic types, i.e. *indica* (blue), *japonica* (red) and intermediary (green). (b–d) Using the matrix, dynamic analysis was performed to study traits such as CCI, ExG and VARI, demonstrating their different growth patterns and the time points when their maximum values were reached, e.g. ${\text{Max}}_{\text{CCI}}$ (85–95 d after sowing, DAS), ${\text{Max}}_{\text{ExG}}$ (73–79 DAS) and ${\text{Max}}_{\text{VARI}}$ (74–78 DAS).

In order to analyze dynamic traits effectively, we created a new function to help reorganize plant genotypes. For example, by extracting plot‐level images from the 254 rice landraces and inserting them into a matrix according to their domestic groups, a ‘performance matrix’ was created using eight orthomosaics collected between 20 July and 8 October 2019 (Fig. 7a). In the matrix, each cell was an overhead image of a rice genotype, such that genotypes were columns and phenotyping timepoints were rows (see the entire 2019 performance matrix in Notes S11).

We used the matrix to examine different traits. The first row was utilized to quantify plot‐based seedling number (Dataset S3; Fig. S2). To perform dynamic analysis of traits such as CCI, we used Gaussian‐fitted curves to study the increase of CCI until 100% coverage was reached (Fig. 7b), based on which ${\text{FGR}}_{\text{CCI}}$, ${\text{AGR}}_{\text{CCI}}$ and the ${\text{Max}}_{\text{CCI}}$ day were computed (Dataset S4). For spectral traits such as ExG and visible atmospherically resistant index (VARI), their associated dynamic traits were also quantified (Fig. 7c,d; Datasets S5, S6). Likewise, the matrix was employed to estimate dynamic changes for other indices (Dataset S7; Fig. S4). Noticeably, to estimate height‐related traits (e.g. ${\text{AGR}}_{\text{height}}$, ${\text{RGP}}_{\text{height}}$ and ${\text{FGR}}_{\text{height}}$; Dataset S9), both the matrix and associated CHMs were used.

### Estimation of heading date

Based on dynamic traits, we explored the estimation of a complex trait, heading date. We predicted the trait using multiple dynamic traits (e.g. height, CCI and VARI) and machine‐learning modelling (Notes S12), including: trait selection (Fig. S5a), dynamic trait analysis (Fig. S5b), feature engineering and selection (Fig. S5c), model training and selection (Fig. S5d), and model validation (Fig. S5e). Among many models tested, support vector regression (SVR) obtained the best coefficient of determination (*R* ^2^ = 0.725; with *P* < 0.001 in linear regression; Notes S12).

### Validation of AirMeasurer‐derived traits

**Fig. 8:**
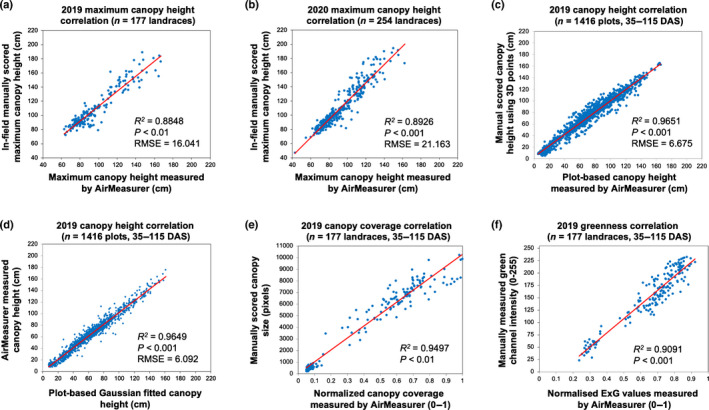
Coefficient of determination (*R* ^2^) computed to evaluate correlations between AirMeasurer‐derived and manually scored maximum plant height, normalized canopy coverage index (CCI) and normalized excess green index (ExG). The correlations between AirMeasurer‐derived, Gaussian‐fitted and manually measured canopy height values also are provided. (a) Plot‐based correlation between the maximum height measured by AirMeasurer and manual scoring using 177 rice landraces in the 2019 season. (b) Correlation between the maximum height trait measured by AirMeasurer and manual scoring using 254 landraces in the 2020 season. (c) Correlation between AirMeasurer‐derived canopy height values (based on calibrated 3D point clouds) and manual scoring of point cloud data to derive canopy height values using 177 landraces measured at eight time points (35–115 d after sowing (DAS)) across the 2019 season, 1416 plots in total; and (d) correlation between AirMeasurer‐derived canopy height values and Gaussian‐fitted values. (e) Correlation between normalized canopy coverage index (0–1) measured by AirMeasurer and the manually scored canopy area of plot images (in pixels) using 177 plots in 2019. (f) Correlation between normalized ExG index (0–1) measured by AirMeasurer and manually measured green values (0–255) using 177 plot images (35–115 DAS) in 2019.

AirMeasurer‐derived traits were validated by a range of ground truth data. The correlation between the 2019‐AirMeasurer‐derived ${\text{Max}}_{\text{height}}$ and the 2019‐manual‐scored ${\text{Max}}_{\text{height}}$ was visualized using 177 landraces (*R* ^2^ = 0.8848, *P* < 0.001; root mean square error, RMSE = 16.041; Fig. 8a), showing a very strong positive correlation. We repeated the validation with 254 landraces measured in the field in 2020. A similar *R* ^2^ was obtained (0.8926, *P* < 0.001, RMSE = 21.163; Fig. 8b). Owing to different methods, AirMeasurer‐derived height values were consistently shorter than manual scoring, which was expected as plants were straightened in manual scoring. The result indicates that AirMeasurer could soundly estimate maximum plant height among diverse rice genotypes (including landraces) with a high accuracy. We also verified the AirMeasurer‐measured height at eight time points (35–115 DAS) using 1416 plots (177 plots per time point) against plot‐based canopy height that was measured manually from eight 3D point clouds (*R* ^2^ = 0.9651, *P* < 0.001, RMSE = 6.675; Fig. 8c), as well as against the Gaussian‐fitted canopy height (*n* = 1416 plots; *R* ^2^ = 0.9649, *P* < 0.001, RMSE = 6.092; Fig. 8d). Significant positive correlations were obtained, indicating the reliability of the AirMeasurer‐estimated height trait throughout the season. For traits such as ExG and CCI that also were used for genetic mapping, 177 plots (at six growth stages, with 29–30 plots per stage) were used to compare the two traits obtained by manual and AirMeasurer‐based approaches, both resulting in strong correlations: *R* ^2^ = 0.9497 for CCI (*P* < 0.01; Fig. 8e) and *R* ^2^ = 0.9091 for ExG (*P* < 0.001; Fig. 8f).

### QTL mapping using height‐related traits

**Fig. 9:**
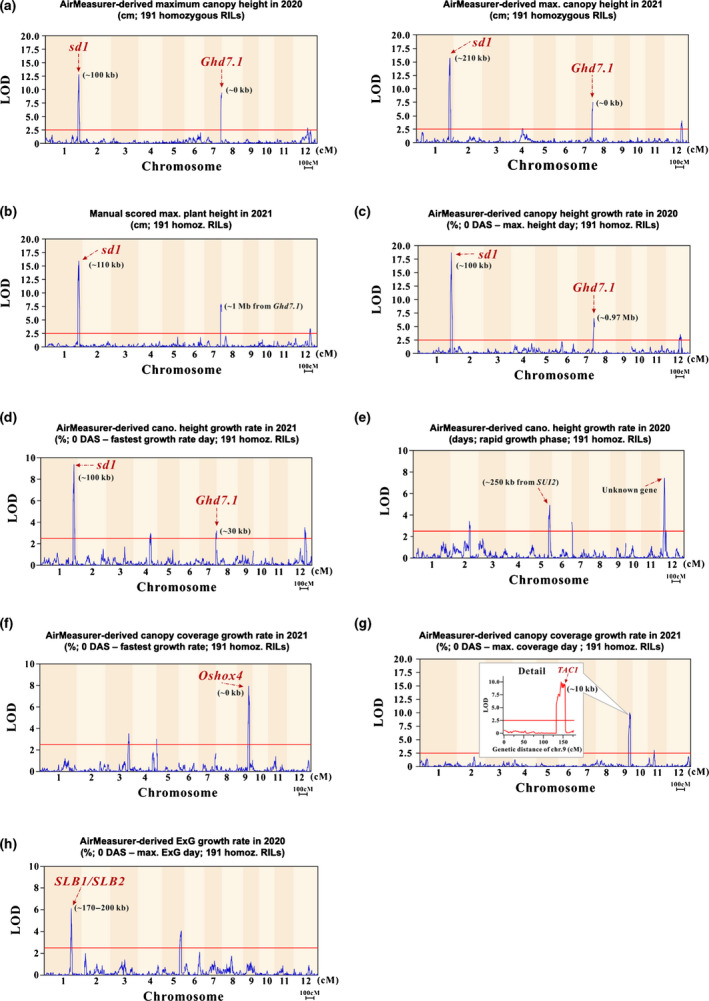
Genetic linkage analysis of various AirMeasurer‐derived growth‐related traits and manually scored maximum plant height, collected from 191 homozygous recombinant inbred lines (RILs) trialled in 2020 and 2021. For the significant single‐nucleotide polymorphisms (SNPs) identified, known genes are indicated by red arrows. (a) Chromosomal location of significant quantitative trait locus (QTLs) identified using AirMeasurer‐derived ${\text{Max}}_{\text{height}}$ trait in 2020. The *x*‐axis denotes the genetic distance of 12 chromosomes and *y*‐axis the logarithm of the odds (LOD) value, with a significant threshold set at 2.5 (red horizontal line). The QTLs are close to the *sd1* gene (chromosome 1) and the *Ghd7.1* gene (chromosome 7). (b) Height QTLs identified using manually measured maximum plant height in the 2021 season; these also were located close to the *sd1* and *Ghd7.1* genes. (c) QTL for the ${\text{AGR}}_{\text{height}}$ trait, between 0 d after sowing (DAS) and the ${\text{Max}}_{\text{height}}$ day, in 2020. (d) QTL for the ${\text{AGR}}_{\text{height}}$ trait (0 DAS – the ${\text{FGR}}_{\text{height}}$ day) in 2021. (e) Four loci associated with the ${\text{RGP}}_{\text{height}}$ trait collected in the 2020 season, including one located near *SUI2* (chromosome 5), and another significant locus on chromosome 12 that is not associated with any known gene. (f) Two QTLs for the ${\text{AGR}}_{\text{CCI}}$ in 2021, determined over the period between 0 DAS and the ${\text{FGR}}_{\text{CCI}}$ day. The major QTL co‐locates with *Oshox4*. (g) QTL for the average growth rate of CCI in 2021 determined over the period between 0 DAS and the ${\text{Max}}_{\text{CCI}}$ day. One strong locus on chromosome 9 (three peaks between 19.2 Mb and 21.6 Mb) co‐locates with a known gene (*TAC1*) that controls canopy structure, and *LGD1* that regulates vegetative growth in rice. (h) QTLs for the ${\text{AGR}}_{\text{ExG}}$ trait for the interval 0 DAS – the ${\text{Max}}_{\text{ExG}}$ day. The major QTL co‐locates with *SLB1*/*SLB2* and *D61*. Table 2 summarizes the QTLs associated with the above growth‐related traits. Abbreviations: maximum canopy height (${\text{Max}}_{\text{height}}$; cm), average growth rate for a target trait (${\mathrm{AGR}}_{\text{trait}}$; %), the fastest growth rate of canopy height (${\mathrm{FGR}}_{\text{height}}$; %), the rapid growth phase (${\text{RGP}}_{\text{height}}$; days), canopy coverage index (CCI), excess green (ExG), maximum CCI (${\text{Max}}_{\text{CCI}}$), maximum ExG (${\text{Max}}_{\mathrm{ExG}}$) and the fastest growth rate of CCI (${\text{FGR}}_{\text{CCI}}$; %).

In order to evaluate the biological relevance of AirMeasurer‐derived traits in genetic mapping studies, we first used the overlapped 191 homozygous RILs in 2020 and 2021 season for genetic linkage analysis. The AirMeasurer‐derived ${\text{Max}}_{\text{height}}$ trait was used to map QTLs in the population, with the *x*‐axis representing the genetic distance of 12 chromosomes and *y*‐axis the LOD value. The threshold (red horizontal line) was set as 2.5 and known loci were indicated with red arrows. Two QTLs related to ${\text{Max}}_{\text{height}}$ were identified (Fig. 9a), among which one significant QTL (LOD = 15.7; chromosome 1) indicated a locus controlling rice plant height in the two seasons, consistent with the QTL mapping using the 2021 manual data and reported previously using the same population (Wang *et al*., 2011). In fact, there is a known gene *sd1* that shortens rice stems (Sasaki *et al*., 2002), *c*. 100–210 kb from the locus. The second highest peak located using the ${\text{Max}}_{\text{height}}$ trait was on chromosome 7 (LOD = 7.3), *c*. 0 kb from *Ghd7.1*, a gene plays an important role in grain productivity and rice heading (Yan *et al*., 2013); however, the second peak identified with the manual scoring was *c*. 1 Mb from *Ghd7.1* (LOD = 7.9; Fig. 9b).

Besides the static trait, we applied the dynamic trait, ${\text{AGR}}_{\text{height}}$, to map QTLs and several QTLs were detected. Using the 2020 ${\text{AGR}}_{\text{height}}$ (0 DAS – the ${\text{Max}}_{\text{height}}$ day), two QTLs were identified (Fig. 9c): *c*. 110 kb from *sd1* (LOD = 18.6) and *c*. 0.97 mb from *Ghd7.1* (LOD = 6.5). The 2021 ${\text{AGR}}_{\text{height}}$ (0 DAS – the ${\text{FGR}}_{\text{height}}$ day) was used and located same QTLs (Fig. 9d): *c*. 100 kb from *sd1* (LOD = 9.3) and *c*. 30 kb from *Ghd7.1* (LOD = 3.2). In particular, we identified four loci with the 2020 ${\text{RGP}}_{\text{height}}$ trait (Fig. 9e), two strong signals are: (1) *c*. 250 kb from *SUI2* (LOD = 4.9), which regulates rice stem development (Virlet *et al*., 2017); (2) *c*. 3.7 Mb on chromosome 12 (LOD = 7.4), which is not associated with any known gene.

### QTL mapping using growth‐related traits

Then, we mapped QTLs using other AirMeasurer‐derived growth traits such as CCI and ExG. The 2021 ${\text{AGR}}_{\text{CCI}}$ (0 DAS – the ${\text{FGR}}_{\text{CCI}}$ day) was used to locate two QTLs (Fig. 9f), including *Oshox4* (*c*. 0 kb; LOD = 7.9), overexpressing the gene leads to dwarfing and increased tillers, and thus the canopy size (Dai *et al*., 2008). One strong locus was identified on chromosome 9 (LOD = 9.6; Fig. 9g) using the 2021 ${\text{AGR}}_{\text{CCI}}$ trait (0 DAS – the ${\text{Max}}_{\text{CCI}}$ day), indicating a locus linking to canopy expansion. Actually, *TAC1* (Yu *et al*., 2007) is *c*. 10 kb away, which controls a spread‐out or compact plant architecture. The QTL (19.2–21.6 Mb) had three peaks: 20.05 Mb, 21.05 Mb and 21.55 Mb, respectively (detailed in Fig. 9g); besides *TAC1*, there is a gene at 20.07 Mb, *LGD1* (Thangasamy *et al*., 2012), which regulates vegetative growth. By mapping QTLs using the 2020 ${\text{AGR}}_{\text{ExG}}$ (0 DAS – ${\text{Max}}_{\text{ExG}}$ day), several vegetation‐related QTLs were identified on chromosome 1 (LOD = 6.1; Fig. 9h), including: (1) *SLB1* and *SLB2* (Cardoso *et al*., 2014), controlling tillering, and (2) *D61* (Yamamuro *et al*., 2000), connected with internode elongation. All of the QTLs identified through QTL mapping are listed in Table 2.

**Table 2:** Quantitative trait loci (QTLs) identified using the height‐ and growth‐related traits in the recombinant inbred line (RIL) population of 191 homozygous lines.

Traits	Year	chr.	Peak gent. pos.	IRGSP4.0 (Mb)	LOD	*R* ^2^	Genes
${\text{Max}}_{\text{height}}$ (cm), AirMeasurer	2020	1	374.0	40.25	12.8	22.3%	*sd1* (40.14 Mb)
		7	195.3	30.20	9.4	15.4%	*Ghd7.1* (30.28 Mb)
		12	162.6	17.70	2.9	4.1%	
${\text{Max}}_{\text{height}}\;$(cm), AirMeasurer	2021	1	374.0	40.25	15.7	25.5%	*sd1* (40.14 Mb)
		7	195.3	30.20	7.3	10.9%	*Ghd7.1* (30.28 Mb)
		12	195.7	22.05	4.0	5.9%	
Maximum plant height (cm), manual	2021	1	374.3	40.35	15.9	24.2%	*sd1* (40.14 Mb)
		7	187.7	29.25	7.9	10.7%	*Ghd7.1* (30.28 Mb)
		12	190.2	21.10	3.4	4.3%	
		12	195.7	22.05	3.3	4.3%	
${\text{AGR}}_{\text{height}}$ (%; 0 DAS – the ${\text{Max}}_{\text{height}}$ day), AirMeasurer	2020	1	374.0	40.25	18.6	30.0%	*sd1* (40.14 Mb)
		7	188.7	29.30	6.5	8.9%	*Ghd7.1* (30.27 Mb)
		12	162.6	17.70	3.5	4.6%	*Xa25*/Os12g0476200 (17.4 Mb)
${\text{AGR}}_{\text{height}}$ (%; 0 DAS – the ${\text{FGR}}_{\text{height}}$ day), AirMeasurer	2021	1	374.0	40.25	9.3	16.8%	*sd1* (40.14 Mb)
		7	196.3	30.30	3.2	5.3%	*Ghd7.1* (30.27 Mb)
		12	191.2	21.25	3.5	5.8%	*OsVIL2*/Os12g0533500 (21.39 Mb)
${\text{RGP}}_{\text{height}}$ (days), AirMeasurer	2020	2	216.7	26.40	3.4	6.0%	*OsYABBY4*/Os02g0643200 (26.7 Mb)
		5	225.4	27.85	4.9	8.9%	*SUI2*/Os05g0554400 (27.6 Mb)
		7	0.0	0.50	3.3	5.9%	*OsGA2ox5*/Os07g0103500 (0.2 Mb)
		12	49.9	3.70	7.4	14.6%	Unknown
${\text{AGR}}_{\text{ExG}}$ (%; 0 DAS – the ${\text{Max}}_{\text{ExG}}$ day)	2020	1	301.7	30.60	6.1	12.4%	*SLB1*(30.77 Mb), *SLB2*(30.8 Mb), *D61*(31.6 Mb)
		5	216.6	27.30	4.1	8.0%	*SUI2*/Os05g0554400 (27.6 Mb)
${\text{AGR}}_{\text{CCI}}$ (%; 0 DAS – the ${\text{Max}}_{\text{CCI}}$ day)	2021	9	145.1	20.05	10.0	17.4%	*LGD1*/Os09g0502100 (20.07 Mb)
		9	149.5	20.95	9.7	16.9%	*OsMADS7*/Os09g0507200 (20.3 Mb)
		9	154.3	21.55	9.6	16.8%	*TAC1* (21.56 Mb)
		11	72.2–72.9	6.4–6.8	3.1	4.8%	*Pia*/Os11g0225100 (6.5 Mb)
${\text{AGR}}_{\text{CCI}}$ (%; 0 DAS – the ${\text{FGR}}_{\text{CCI}}$ day)	2021	3	264.0–264.4	31.35–31.6	3.5	6.1%	*OsIAA13*/Os03g0742900 (31.24 Mb)
		9	131.7	18.55	7.9	14.5%	*OsZHD1* (18.35 Mb), *Oshox4* (18.55 Mb), *OsbZIP73* (18.77 Mb)

### GWAS using height‐related traits

**Fig. 10:**
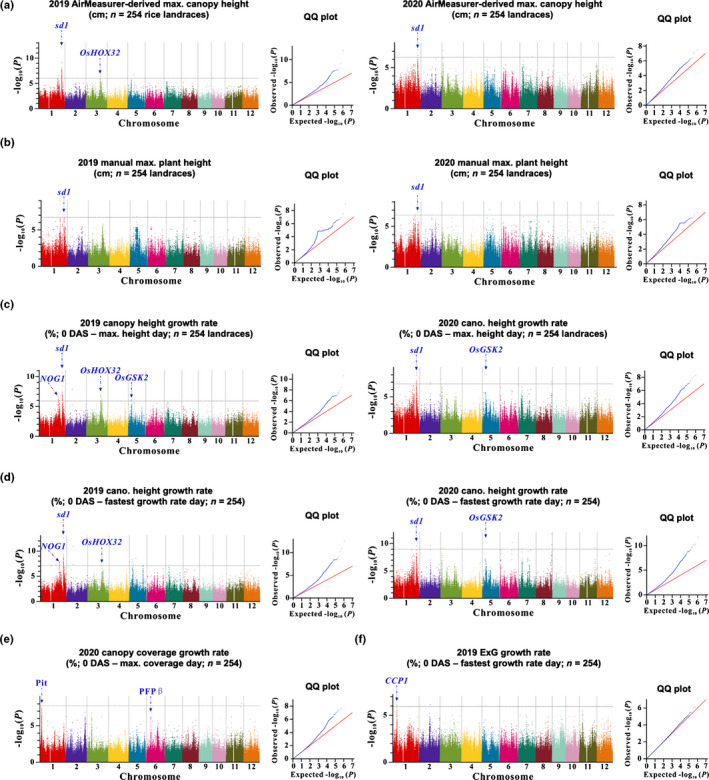
Manhattan plots and quantile‐quantile (QQ) plots for AirMeasurer‐derived traits subjected to a genome‐wide association study (GWAS) of 254 rice landraces trialled in 2019 and 2020. The significance threshold is shown by the horizontal grey dotted line. Known genes that co‐locate with significant loci are indicated by blue arrows. See Fig. 9 legend for trait abbreviations. (a) Manhattan plot and a QQ plot for the AirMeasurer‐derived ${\text{Max}}_{\text{height}}$ trait measured in 2019. The strongest signal on chromosome 1 was close to the *sd1* gene and a strong signal on chromosome 3 was close to the *OsHox32* gene. (b) Manhattan plot for the manually scored maximum plant height trait collected in the 2019 and 2020 seasons. (c) The 2019 ${\text{AGR}}_{\text{height}}$ (0 DAS – the ${\text{Max}}_{\text{height}}$ day) was used to identify four significant SNPs, co‐locating with known genes: *sd1*, *OsHox32* (chromosome 3), *NOG1* (chromosome 1) and *OsGSK2* (chromosome 5). Analysis repeated using the same trait collected in 2020 and reproduced two SNPs, close to *sd1* and *OsGSK2*. (d) GWAS performed with the trait ${\text{AGR}}_{\text{height}}$ (0 DAS – the ${\text{FGR}}_{\text{height}}$ day). Similar results were obtained in both seasons. (e) Plots for the 2020 ${\text{AGR}}_{\text{CCI}}$ (0 DAS – the ${\text{Max}}_{\text{CCI}}$ day) trait. Two signals were identified, one close to the *Pit* gene on chromosome 1 and the other near the *PFPβ* gene on chromosome 6. (f) In the analysis of the 2019 ${\text{AGR}}_{\text{ExG}}$ trait (0 DAS – the ${\text{FGR}}_{\text{ExG}}$ day), the strongest signal co‐located with the *CCP1* gene on chromosome 1. Table 3 lists all of the significant association signals of the above growth‐related traits. DAS, days after sowing.

Besides QTL mapping, we utilized AirMeasurer‐derived traits in GWAS analysis with the 254 rice landraces. We identified several significant SNPs associated with the two‐season height‐related traits and presented them in the Manhattan plot and QQ plot, with a grey dotted line indicating the threshold of the genome‐wide significant *P*‐value (Table S1) and a false detection rate (FDR) of 0.2. For example, using the 2019 ${\text{Max}}_{\text{height}}$ trait, the strongest signal on chromosome 1 (−log_10_(*P*) = 12, indicated with a blue arrow; Fig. 10a, left) was *c*. 208 kb from *sd1*. On chromosome 3, the strongest signal (−log_10_(*P*) = 6.42) identified was *c*. 10.7 kb from the *OsHox32* gene, which is known for pleiotropic effects on plant architecture and leaf development (Chen *et al*., 2021). We repeated the analysis using the 2020 ${\text{Max}}_{\text{height}}$ trait and produced similar results on chromosome 1 (*c*. 208 kb from *sd1*; −log_10_(*P*) = 7.14; Fig. 10a, right). The findings were consistent with the GWAS analysis using the two‐season manual ${\text{Max}}_{\text{height}}$ scoring (*c*. 202–208 kb from *sd1*; −log_10_(*P*) = 6.91–6.63; Fig. 10b).

Next, we chose the 2019 ${\text{AGR}}_{\text{height}}$ (0 DAS – the ${\text{Max}}_{\text{height}}$ day) and identified four significant SNPs associated with the trait (Fig. 10c, left). Besides the strongest signal (−log_10_(*P*) = 10.62; *c*. 208 kb from *sd1*), the second strongest signal (−log10(*P*) = 6.98) was *c*. 11 kb from *OsHox32* on chromosome 3, followed by a strong signal (−log10(*P*) = 6.6) *c*. 122 kb from *NOG1* on chromosome 1, and the last one *c*. 373 kb from *OsGSK2* on chromosome 5. The *NOG1* gene increases rice grain production (Huo *et al*., 2017), whereas *OsGSK2* regulates the mesocotyl length (Sun *et al*., 2018), both genes relate to plant growth and development. We repeated the analysis using the 2020 data (Fig. 10c, right) and reproduced two SNPs that close to *sd1* (−log10(*P*) = 8.29; *c*. 188 kb) and *OsGSK2* (−log10(*P*) = 8.07; *c*. 373 kb). To test other height‐related dynamic traits, we used the two‐season ${\text{AGR}}_{\text{height}}$ (0 DAS – the ${\text{FGR}}_{\text{height}}$ day) and obtained similar results (Fig. 10d), with a significant SNP on chromosome 5 repeatedly located (−log10(*P*) = 8.28–10.28; *c*. 306.106–372.984 away from *OsGSK2*). Table 3 lists all the significant signals associated with the above traits.

**Table 3:** Genome‐wide significant association signals of height‐related traits collected from 254 rice landraces using EMMAx.

Trait	Yr.	Chr.	Position^a^	−log_10_ *P*	Distance^b^ (kb)	Candidate genes	Gene symbol
${\text{Max}}_{\text{height}}$ (cm), AirMeasurer	19	1	40 349 753	12	208.421	Os01g0883800	*sd1*
		3	25 428 611	6.42	10.659	Os03g0640800	*OsHox32*
	20	1	40 349 753	7.14	208.421	Os01g0883800	*sd1*
		3	2178 057	7.03			
		9	15 190 326	7.46			
Maximum plant height (cm), manual	19	1	33 179 314	9			
		1	40 343 458	6.91	202.126	Os01g0883800	*sd1*
	20	1	35 921 458	6.21			
		1	40 349 753	6.63	208.421	Os01g0883800	*sd1*
${\text{AGR}}_{\text{height}}$ (%; 0 DAS – the ${\mathrm{Max}}_{\text{height}}$ day), AirMeasurer	19	1	33 186 547	6.6	122.199	Os01g0752200	*NOG1*
		1	40 349 753	10.62	208.421	Os01g0883800	*sd1*
		3	25 429 147	6.98	11.195	Os03g0640800	*OsHox32*
		3	27 284 658	6.44			
		5	6266 446	6.09	373.094	Os05g0207500	*OsGSK2*
	20	1	40 329 637	8.29	188.305	Os01g0883800	*sd1*
		2	19 202 188	6.97			
		3	2178 057	7.53			
		5	6266 556	8.07	372.984	Os05g0207500	*OsGSK2*
${\text{AGR}}_{\text{height}}$ (%; 0 DAS – the ${\mathrm{FRG}}_{\text{height}}$ day), AirMeasurer	19	1	33 186 547	7.76	122.199	Os01g0752200	*NOG1*
		1	40 349 753	12.57	208.421	Os01g0883800	*sd1*
		3	25 417 528	7.21	0.424	Os03g0640800	*OsHox32*
		5	5013 282	8.28	306.106	Os05g0207500	*OsGSK2*
		5	24 055 973	7.14			
	20	1	40 329 637	9.56	188.305	Os01g0883800	*sd1*
		2	19 202 188	9.53			
		3	2178 057	9.11			
		5	626 656	10.28	372.984	Os05g0207500	*OsGSK2*
		7	6156 842	9.01			

^a^ Position in bp according to IRGSP 4.0.

^b^ The distance between SNP and candidate gene. DAS, d after sowing.

### GWAS using other growth‐related traits

Finally, we chose the AirMeasurer‐derived CCI and ExG traits to perform GWAS and found three SNPs (Table S2). Using the 2020 ${\text{AGR}}_{\text{CCI}}$ trait (0 DAS – the ${\text{Max}}_{\text{CCI}}$ day), two signals were identified (Fig. 10e): (1) one signal (−log_10_(*P*) = 7.4) was *c*. 329 kb from the *Pit* gene on chromosome 1, a disease resistance gene (Hayashi & Yoshida, 2009); (2) another (−log_10_(*P*) = 6.49) was *c*. 224 kb from the *PFPβ* gene on chromosome 6, which associates with carbon metabolism during grain‐filling (Duan *et al*., 2016). Using the 2019 ${\text{AGR}}_{\text{ExG}}$ trait (0 DAS – the ${\text{FGR}}_{\text{ExG}}$ day), the strongest signal (−log_10_(*P*) = 6.07) was *c*. 304 kb from the *CCP1* gene on chromosome 1 (Fig. 10f), which functions palea development (Yan *et al*., 2015). Furthermore, GWAS was attempted with the 2019‐heading‐date trait estimated by both manual and AirMeasurer approaches (Notes S13).

## Discussion

In order to exploit available genomic resources to address climate change challenges, selected traits need to be assessed under field conditions across locations and years. Conventional phenotyping requires making many measurements of target traits, which is arduous and difficult to implement at busy periods of the season, resulting in newly developed methods (Pieruschka & Schurr, 2019; Jang *et al*., 2020). This study demonstrates that the use of low‐cost UAVs can acquire larger and regular plant data from the field, based on which high‐quality 2D/3D aerial imagery with field‐ and plot‐level resolutions can be generated to enable automated analysis of static and dynamic traits that are biologically relevant. Furthermore, this approach is potentially valuable for assessing rates of genetic gain in larger trials, facilitating the calculation of heritability for agronomic traits and accurate genetic mapping for developing molecular markers. Nevertheless, metrics such as economic costs, scalability, analysis accuracy and throughput, or processing time need to be considered to evaluate the above research objectives, which is beyond the scope of this study but important for future studies.

### Static and dynamic traits

The use of AirMeasurer helped us analyze target traits coherently, which was achieved by removing unwanted field‐level terrain features, transferring 3D to 2D signals for efficient processing, analyzing 3D points at different vertical levels and identifying plots consistently. These methodological advances were proved to be useful in examining static traits (e.g. height, CCI and ExG) at different growth stages for paddy rice (in particular the landraces), which was complex to study during the season.

Inspired by previous work (Würschum *et al*., 2014; Anderson *et al*., 2019), we developed a bespoke approach to estimate dynamic traits derived from time series measures of static traits, enabling us to gain insights into dynamic features (e.g. growth rate and RGP) of target traits, without excessive phenotyping. Instead of using phenotypes measured at arbitrary time points, dynamic analysis helped us evaluate phenotypic variation reliably with hundreds of genotypes.

Additionally, through integrating dynamic traits into machine‐learning modelling, we predicted a complex trait, heading date, which could lead to new estimates of QTL × Environment interactions. Recent studies (Lowry *et al*., 2019; Mu *et al*., 2022) have reported similar approaches that used multi‐location traits in QTL mapping. Also, we demonstrated that AirMeasurer‐derived traits could be used for multiseason QTL discovery, which was confirmed by the results highlighting the locations of known QTLs. Although the main objective of this study was *not* to discover novel QTLs, nor to validate robustness of such QTLs across different germplasm sets and environments, it would only require simple adjustments to trial designs (e.g. more replicates) and greater repetition of trials across locations and years in order to produce reliable estimates of trait heritability and QTLs.

### AirMeasurer as a research tool

Genetic mapping of dynamic or longitudinal traits can be a powerful tool for developing novel molecular markers that cannot easily be revealed using static measurement, partly because of temporal regulation of gene expression (Harder *et al*., 2019). We explored the use of AirMeasurer‐derived traits to identify associated loci. For example, QTLs were mapped for traits such as ${\text{Max}}_{\text{height}}$, ${\text{AGR}}_{\text{height}}$ during ${\text{RGP}}_{\text{height}}$ or at the ${\text{FGR}}_{\text{height}}$ day. If the QTLs are shown to be robust across years, locations and different germplasm sets, these then could be used to develop growth‐related molecular markers. Some of the identified loci were co‐located with known genes, as well as with other genes within the interval had unknown functions, which could lead to new candidate genes. QTLs located using traits derived from CCI and ExG (e.g. ${\text{Max}}_{\text{cov}}$, ${\text{FGR}}_{\text{cov}}$ and ${\text{Max}}_{\text{ExG}}$) also indicate potentially useful loci.

Likewise, we used AirMeasurer‐derived traits in GWAS analysis. Comparable loci were identified from rice landraces. Height‐related trait (e.g. ${\text{AGR}}_{\text{height}}$) led to the consistent identification of signals such as the nearby genes (e.g. *sd1*, *OsHox32*, *NOG1* and *OsGSK2*) relevant to plant height, architecture and growth regulation, indicating the value of dynamic traits in studying genetically diverse landrace populations. Moreover, using dynamic traits such as ${\text{AGR}}_{\text{height}}$, ${\text{Max}}_{\text{cov}}$ and ${\text{FGR}}_{\text{ExG}}$, we located some previously unknown strong signals, which may be valuable for identifying small effects of individual allelic differences (e.g. loci on Chromosome 2; Fig. 10c,d) that jointly contribute to the regulation of trait expressions. Finally, AirMeasurer‐estimated heading date traits could bring a new perspective to GWAS analysis. Loci identified using a small number of *indica* landraces (*n* = 97) were just *c*. 17.14 kb from *OsSOC1* gene on chromosome 3 (−log_10_(*P*) = 5.79) and *c*. 30.24 kb from the *Hd3a* gene on chromosome 6 (−log_10_(*P*) = 4.65), both of which are heading‐date related (Notes S13).

### Limitations of the platform

We have encountered problems that are not uncommon when applying drones in aerial phenotyping: (1) *weather conditions* – small UAVs cannot be operated in unstable weather such as high or gusty wind (> 15 ms^−1^), rainfall, or heavy fog (Tmušić *et al*., 2020); (2) *geo‐referencing* – GPS modules installed on low‐cost drones had metre‐level deviation and thus geo‐referencing errors needed to be rectified; (3) *nature illuminance* – image colour and contrast could vary noticeably with changing light conditions, and we mitigated this issue by conducting a field‐level imaging; and (4) *aviation regulations* – the change in aviation regulations casts uncertainty on aerial phenotyping, requiring regular communications with local civil air traffic control authorities; without official authorization, the payload capacity of a drone was restricted, indicating the advantage and practicality in using small drones for routine phenotyping. In the study, we used an RGB camera for growth‐related spectral analysis as a low‐cost alternative to more costly hyper‐ or multispectral sensors. It is worth noting that visible spectra are limited in early diseases detection and sensing abiotic stress responses as accurate spectral information is key to assess plant responses to certain external stimuli (Tmušić *et al*., 2020).

For data pre‐processing, we used the proprietary pix4dmapper software to generate 3D point clouds and 2D orthomosaics. We have tested several open‐source software types (e.g. visualsfm, meshroom) for the same task and encountered technical problems such as prolonged computational time, incorrect geo‐referencing, and mismatched 2D/3D patches. Another problem during the processing was to denoise large‐scale 3D point clouds. We used the SOR for the task, which required 15–20 min to denoise 60+ million 3D points. Hence, algorithms such as local‐outlier and cluster‐based outlier detection (Kriegel *et al*., 2009), and deep‐learning (DL) methods (Casajus *et al*., 2019) should be considered to speed up this task. Although the AirMeasurer's plot segmentation algorithm could reliably be applied to field experiments with regular gridded plot layout designs (Notes S10), it cannot be extended to analyze irregular plot layouts (e.g. zigzag arrangements). DL approaches such as multilayer perceptron that can incorporate multidimensional ground/plant signals might be more useful for this mission.

### Future applications

Further developments could include the analysis of high‐density 3D point clouds. Rice flowering starts 1 d after the heading, during which anthers (1–1.5 mm in diameter) can be observed on different panicles. By flying smaller UAVs (e.g. DJI Mini) at a 4‐m altitude, we could achieve a ground‐sampling‐distance (GSD) of 1–1.5 mm per pixel. Thus, it is feasible to measure anther extrusion using high‐density 3D points acquired by smaller drones, which could find applications in hybrid breeding programmes where the selection of male parents with certain flowering characteristics is crucial.

Low‐cost UAVs and dynamic trait analysis also could be applied to examine traits such as grain‐filling, which are challenging to quantify using conventional approaches. By conducting daily flights during ripening, fitted curves could enable the estimation and eventually the prediction of the initiation and duration of this key trait. However, it is expected that the Gaussian function might not be suitable for such growth patterns, and thus other fitting methods shall be explored.

Although we did not thoroughly test AirMeasurer to analyze other crops, we have successfully applied the platform to examine wheat trials with limited parametric changes (Notes S10, S14), suggesting potential applications of AirMeasurer for other plant species. As the modular‐designed AirMeasurer was developed in Python, which is widely supported, we trust that this platform could be shared, extended and upgraded by the community relatively easily, providing open and readily accessible solutions for the broader research community.

## Competing interests

None declared.

## Author contributions

Ji Zhou wrote the manuscript with inputs from EO, GS, QZ, YZ and HL; Ji Zhou, GS and Jie Zhou designed the AirMeasurer platform and GUI software with help from QZ and HL; GS, Jie Zhou, HL and Ji Zhou implemented the software; YW, AW, GS and LX performed the rice experiments and UAV‐based field phenotyping under BH, QZ and Ji Zhou's supervision; RJ and EO provided expertise in aerial imaging and crop modelling; GS, Jie Zhou and HL tested and optimised the software; Ji Zhou, GS, EO, JC and QZ performed the data analysis and modelling; and YZ, AW, HL, Ji Zhou and QZ performed GWAS analysis and QTL mapping under BH's supervision. All authors read and approved the final manuscript. GS, HL and YZ contributed equally to this work.

## Funding

HL, YZ, YW, AW, QZ and the rice field experiments were supported by the Chinese Academy of Sciences under BH's supervision (XDA24020205 to QZ). UAV‐based phenotyping was supported by the National Natural Science Foundation of China (32 070 400 to Ji Zhou). RJ and Ji Zhou were partially funded by the United Kingdom Research and Innovation's (UKRI) Biotechnology and Biological Sciences Research Council (BBSRC) Designing Future Wheat Programme (BB/P016855/1). JC was supported by the BBSRC's National Productivity Investment Fund CASE Award, Norwich Research Park's Biosciences Doctoral Training Partnership (BB/M011216/1 to Ji Zhou). GS and Jie Zhou were supported by the Fundamental Research Funds for the Central Universities in China (JCQY201902), as well as by the Jiangsu Collaborative Innovation Center for Modern Crop Production, and the Natural Science Foundation of the Jiangsu Province (BK20191311 to Ji Zhou). Both Ji Zhou and EC were partially supported by a PhenomUK project grant funded by the UKRI (MR/R025746/1 to Ji Zhou).

## Open access

The source code is distributed under the Creative Commons Attribution 4.0 international license, permitting academic use, distribution, reproduction in any medium, provided you give appropriate credit to the original authors and the source, provide a link to the Creative Commons license, and indicate if changes were made. Unless otherwise stated, the Creative Commons Public Domain Dedication (http://creativecommons.org/licenses/by/4.0) waiver applies to the data and results made available in this paper. The source code, testing data, and other datasets supporting the results presented here are available at https://github.com/The‐Zhou‐Lab/UAV/releases/tag/V2.0.2. Other data and user guides are openly available on request.

## Supporting information


**Dataset S1** The 2019 season AirMeasurer‐measured height values.
**Dataset S2** The 2020 season AirMeasurer‐measured height values.
**Dataset S3** The 2019 season AirMeasurer‐estimated seedling number.
**Dataset S4** The 2019 season dynamic analysis of canopy coverage index.
**Dataset S5** The 2019 season dynamic analysis of the excess green index.
**Dataset S6** The 2019 season AirMeasurer‐measured VARI index.
**Dataset S7** The 2019 season AirMeasurer‐measured NDYI index.
**Dataset S8** The 2019 season AirMeasurer‐measured ASM index.
**Dataset S9** The 2019 season dynamic analysis of canopy height.

**Fig. S1** Multilocation phenotyping using low‐cost UAVs, customized flight plans and in‐field setups.
**Fig. S2** Plot‐based seedling measurements to examine the number of seedlings for 241 RILs in January 2020 in the Hainan trial centre.
**Fig. S3** Six overhead 2D orthomosaics, pseudocoloured height maps and 3D point clouds from 241 RILs, showing plant height changes between 15 January and 31 March 2020.
**Fig. S4** Range of vegetative indices and textural traits measured by AirMeasurer from 241 RILs between 15 January and 31 March 2020.
**Fig. S5** Combining phenotypic traits and supervised machine learning to predict a complex trait, heading date, with high confidence.
**Notes S1** Trial design and plant materials.
**Notes S2** UAV imaging protocol and in‐field setups.
**Notes S3** Different plant height measures before and after removing terrain features.
**Notes S4** 3D point clouds processing and canopy height model.
**Notes S5** Previous published segmentation solutions trialed in rice field experiments.
**Notes S6** Source code of the plot segmentation algorithm.
**Notes S7** The reasoning behind choosing *H*_90th_ for height measurement.
**Notes S8** Source code for computing canopy coverage and ExG indices.
**Notes S9** Vegetative indices and texture‐based traits.
**Notes S10** A step‐by‐step user guide of the AirMeasurer GUI.
**Notes S11** Entire performance matrix for all plots monitored.
**Notes S12** Estimation of a complex trait – heading date.
**Notes S13** GWAS using heading dates estimated by the SVR model.
**Notes S14** Applying AirMeasurer to examine wheat plots under different nitrogen treatments.
**Table S1** The genome‐wide significant *P*‐value (FDR0.2).
**Table S2** Genome‐wide significant association (GWAS) signals of ExG and CCI using EMMAx.

**Video S1** GUI of AirMeasurer in operation.
Please note: Wiley Blackwell are not responsible for the content or functionality of any Supporting Information supplied by the authors. Any queries (other than missing material) should be directed to the *New Phytologist* Central Office.

## Data Availability

Project name: AirMeasurer for genetic mapping in crops. Project release page and source code: https://github.com/The‐Zhou‐Lab/UAV/releases/tag/V2.0.2. Testing 2D/3D aerial images: 2D orthomosaics (x8) and 3D point clouds (x8), 11.3 GB in total. GUI software: AirMeasurer_v2.0.2.zip (480 MB). Operating system(s): Windows 10 onwards; the Jupyter notebook can be executed across platforms. Requirements: Python 3.7+; Laspy (1.7.0), Whitebox (1.3.0), Gdal (3.2.1), Rasterio (1.2.0), cSF (1.1.1), Scikit‐Image (0.16.2), OpenCV‐Contrib‐Python (3.4.2.16), Pandas (1.0.1), Numpy (1.18.1), and SciPy (1.4.1).
