# Supplementary material for: AirMeasurer: open‐source software to quantify static and dynamic traits derived from multiseason aerial phenotyping to empower genetic mapping studies in rice
Source: New Phytol. 2022 Jul 28;236(4):1584–604. doi: 10.1111/nph.18314 (PMC9796158; doi:10.1111/nph.18314)
Supplement: Supplementary file 2 — Fig. S1 Multilocation phenotyping using low‐cost UAVs, customized flight plans and in‐field setups. Fig. S2 Plot‐based seedling measurements to examine the number of seedlings for 241 RILs in January 2020 in the Hainan trial centre. Fig. S3 Six overhead 2D orthomosaics, pseudocoloured height maps and 3D point clouds from 241 RILs, showing plant height changes between 15 January and 31 March 2020. Fig. S4 Range of vegetative indices and textural traits measured by AirMeasurer from 241 RILs between 15 January and 31 March 2020. Fig. S5 Combining phenotypic traits and supervised machine learning to predict a complex trait, heading date, with high confidence. Notes S1 Trial design and plant materials. Notes S2 UAV imaging protocol and in‐field setups. Notes S3 Different plant height measures before and after removing terrain features. Notes S4 3D point clouds processing and canopy height model. Notes S5 Previous published segmentation solutions trialed in rice field experiments. Notes S6 Source code of the plot segmentation algorithm. Notes S7 The reasoning behind choosing H 90th for height measurement. Notes S8 Source code for computing canopy coverage and ExG indices. Notes S9 Vegetative indices and texture‐based traits. Notes S10 A step‐by‐step user guide of the AirMeasurer GUI. Notes S11 Entire performance matrix for all plots monitored. Notes S12 Estimation of a complex trait – heading date. Notes S13 GWAS using heading dates estimated by the SVR model. Notes S14 Applying AirMeasurer to examine wheat plots under different nitrogen treatments. Table S1 The genome‐wide significant P‐value (FDR0.2). Table S2 Genome‐wide significant association (GWAS) signals of ExG and CCI using EMMAx. [file NPH-236-1584-s001.pdf]

New Phytologist **Supporting Information**

Article title: AirMeasurer: open-source software to quantify static and dynamic traits derived from multi-season aerial phenotyping to empower genetic mapping studies in rice

Authors: Gang Sun, Hengyun Lu, Yan Zhao, Jie Zhou, Robert Jackson, Yongchun Wang, Ling-xiang Xu, Ahong Wang, Joshua Colmer, Eric Ober, Qiang Zhao, Bin Han, Ji Zhou

Article acceptance date: 31 May 2022

The following Supporting Information is available for this article:

Fig. S1 Multi-location aerial phenotyping using low-cost UAVs, customised flight plans and in-field setups.

Fig. S2 The plot-based seedling measurements to examine the number of seedlings per plot for all 241 RILs (191 homozygous) on January 2020 (21 DAS) in the Hainan trial centre.

Fig. S3 Six overhead 2D orthomosaics, pseudo-coloured height maps, and 3D point clouds reconstructed from 241 RILs (191 homozygous), showing plant height changes between 15th January and 31st March 2020.

Fig. S4 A range of vegetative indices and textural traits measured by AirMeasurer from 241 RILs between 15th January and 31st March 2020.

Fig. S5 Combining phenotypic traits and supervised machine learning to predict a complex trait, heading date, with high confidence.

Table S1 The genome-wide significant *P* value (FDR0.2).

Table S2 Genome-wide significant association (GWAS) signals of ExG and CCI related traits using EMMAx.

Notes S1 Trial design and plant materials.

Notes S2 UAV imaging protocol and in-field setups.

Notes S3 Different plant height measures before and after removing terrain features.

Notes S4 3D point clouds processing and canopy height model.

Notes S5 Previous published segmentation solutions trialed in rice field experiments.

Notes S6 Source code of the plot segmentation algorithm.

Notes S7 The reasoning behind choosing H_{90th} for height measurement.

Notes S8 Source code for computing canopy coverage and ExG indices.

Notes S9 Vegetative indices and texture-based traits.

Notes S10 A step-by-step user guide of the AirMeasurer GUI.

Notes S11 The entire performance matrix for all plots monitored.

Notes S12 Estimation of a complex trait – heading date.

Notes S13 GWAS using heading dates estimated by the SVR model.

Notes S14 Applying AirMeasurer to examine wheat plots under different nitrogen treatments.

Video/Movie S1 The GUI of AirMeasurer in operation.

Supporting Information Figures

Fig. S1 Multi-location aerial phenotyping using low-cost UAVs, customised flight plans and a range of in-field setups.

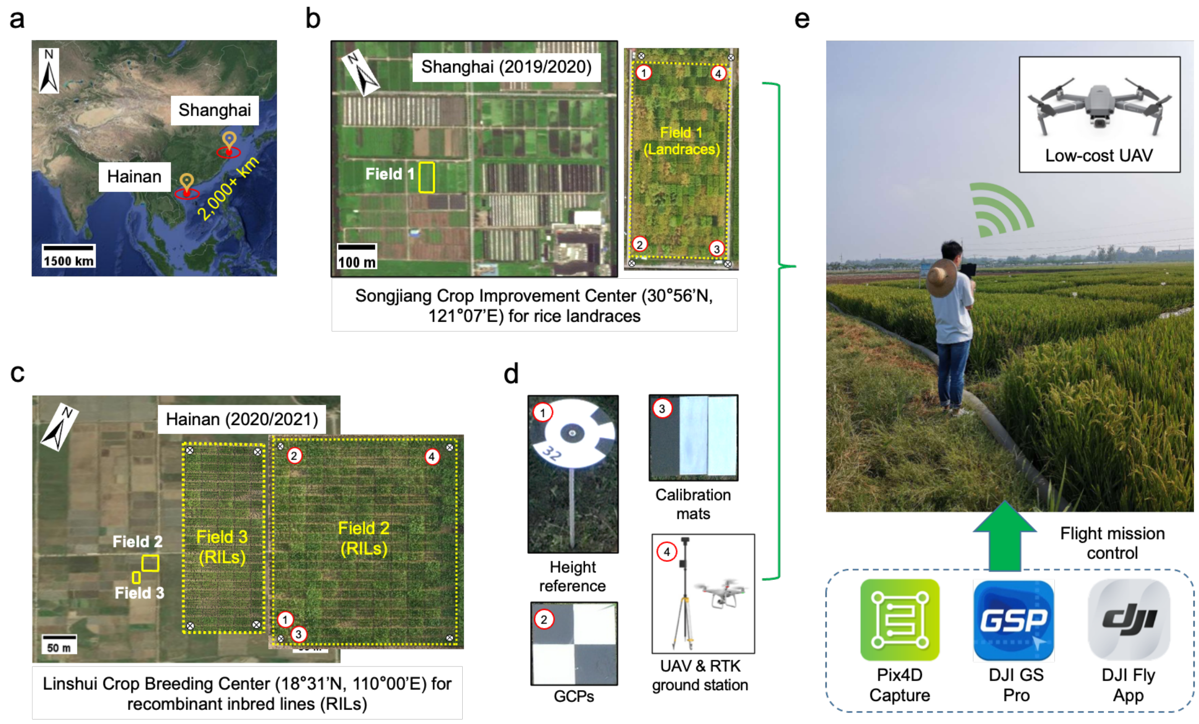

(**a**) Two field experiments were established at two trial centres in the 2019 and 2020 seasons, over 2,000 km apart. (**b**) In the 2019 and 2020 seasons, 254 rice landraces (including 103 *japonica*, 40 intermedia and 111 *indica*.) were sown in Shanghai. (**c**) 191 homozygous recombinant inbred lines (RILs) derived from the crossing parents Nipponbare (*Oryza sativa ssp. japonica*) and 93-11 (*Oryza sativa ssp. indica*) were examined in the 2020 and 2021 seasons, in Hainan. (**d**) Ground control points (GCPs), reflectance calibration mats, and height references were installed in the two fields to help geo-reference UAV images. (**d**) Two flying mission plans were developed to carry out aerial phenotyping to acquire field- and plant-level images using either Pix4D Capture or DJI GS Pro.

Fig. S2 The plot-based seedling measurements to examine the number of seedlings per plot for all the 241 rice RILs on January 2020 (21 days after sowing, DAS) in the Hainan trial centre.

a

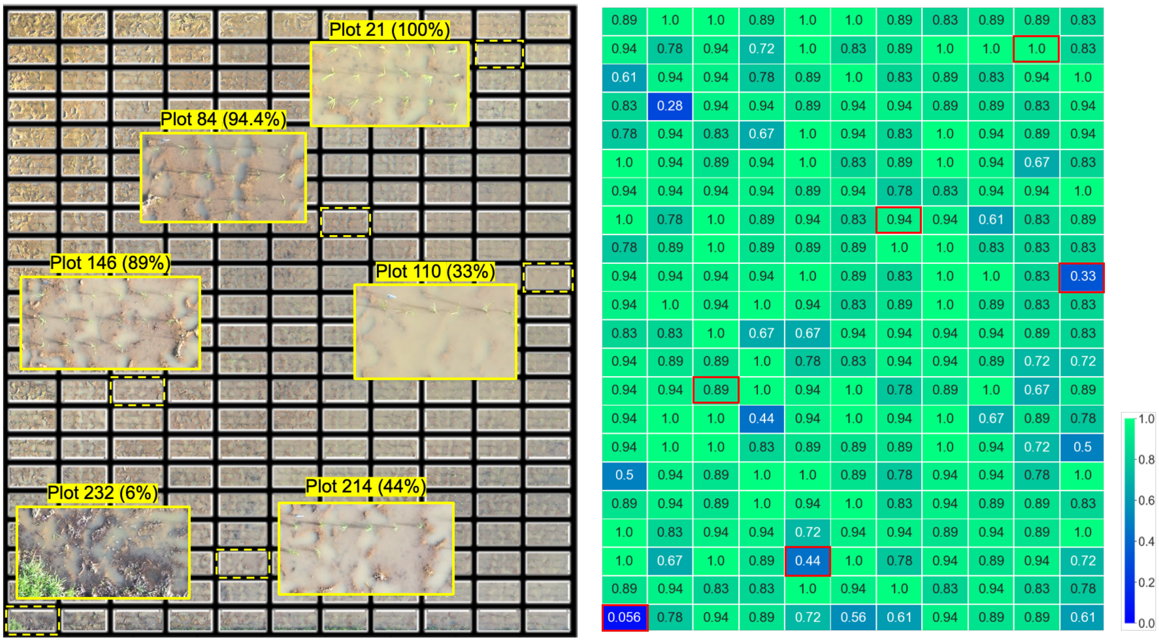

b

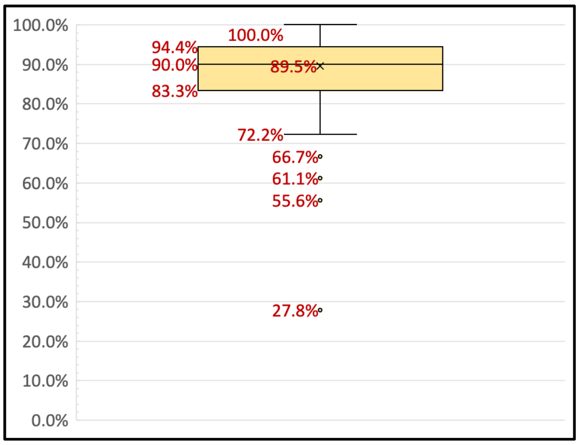

(**a**) An overhead 2D orthomosaic image (left) and pseudo-coloured seedling number map (right) with a seedling emergence percentage scale bar (0-1); the lower left plot was left blank. (**b**) A box-plot with whiskers drawn for 241 plots within the 1.5 interquartile range (IQR) value, demonstrating the locality, spread and skewness groups of the difference between AirMeasurer's seedling scores and manual scoring.

Fig. S3 Six overhead 2D orthomosaics, pseudo-coloured height maps, and 3D point clouds reconstructed from the 241 RILs (191 homozygous), showing rice plant height changes between 15th January and 31st March 2020.

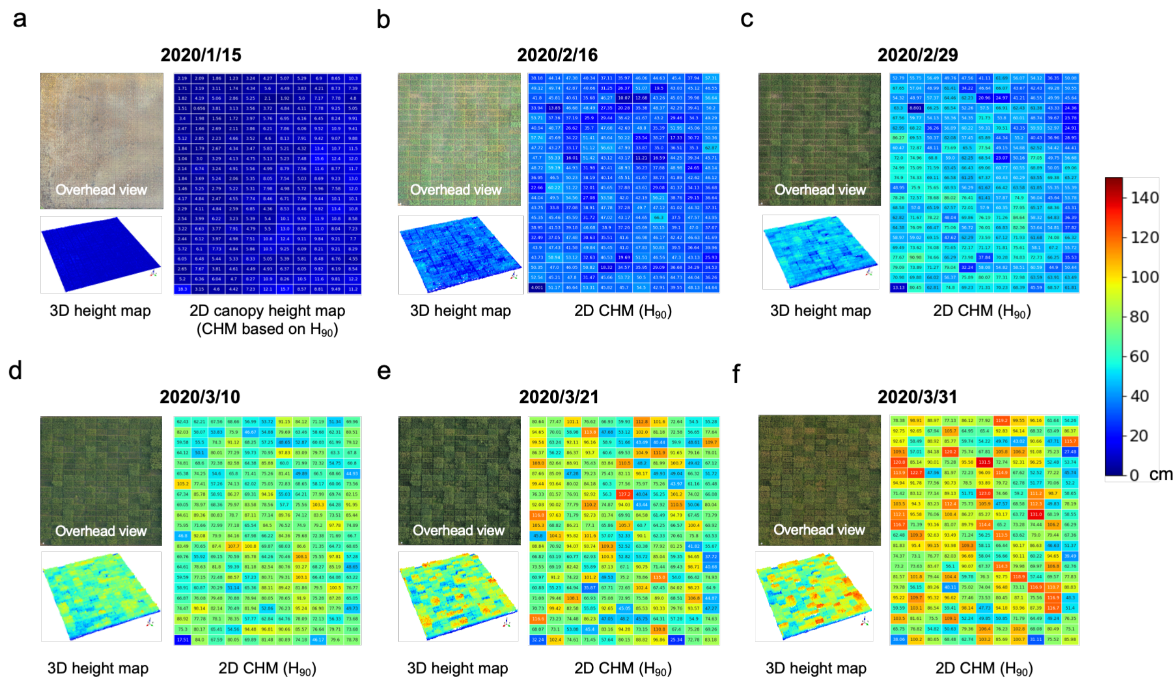

(**a**) 3D point clouds (from a 60-degree perspective) and overhead 2D orthomosaic of the 241 RILs at 21 DAS (15th January 2020) in Hainan (left); the pseudo-coloured plant height map (right), showing height variation between all the RILs; (**b-f**) 3D point clouds and 2D orthomosaics (left) together with the pseudo-coloured height maps (right) between 16th February and 31st March 2020 (53-98 DAS), showing the changes of height values for all the RILs. The unified height scale bar for the six sub-figures is shown (to the right). The lower left plot was left blank

Fig. S4 A range of vegetative indices and textural traits measured by AirMeasurer for the 241 RILs (191 homozygous) between 15th January and 31st March 2020.

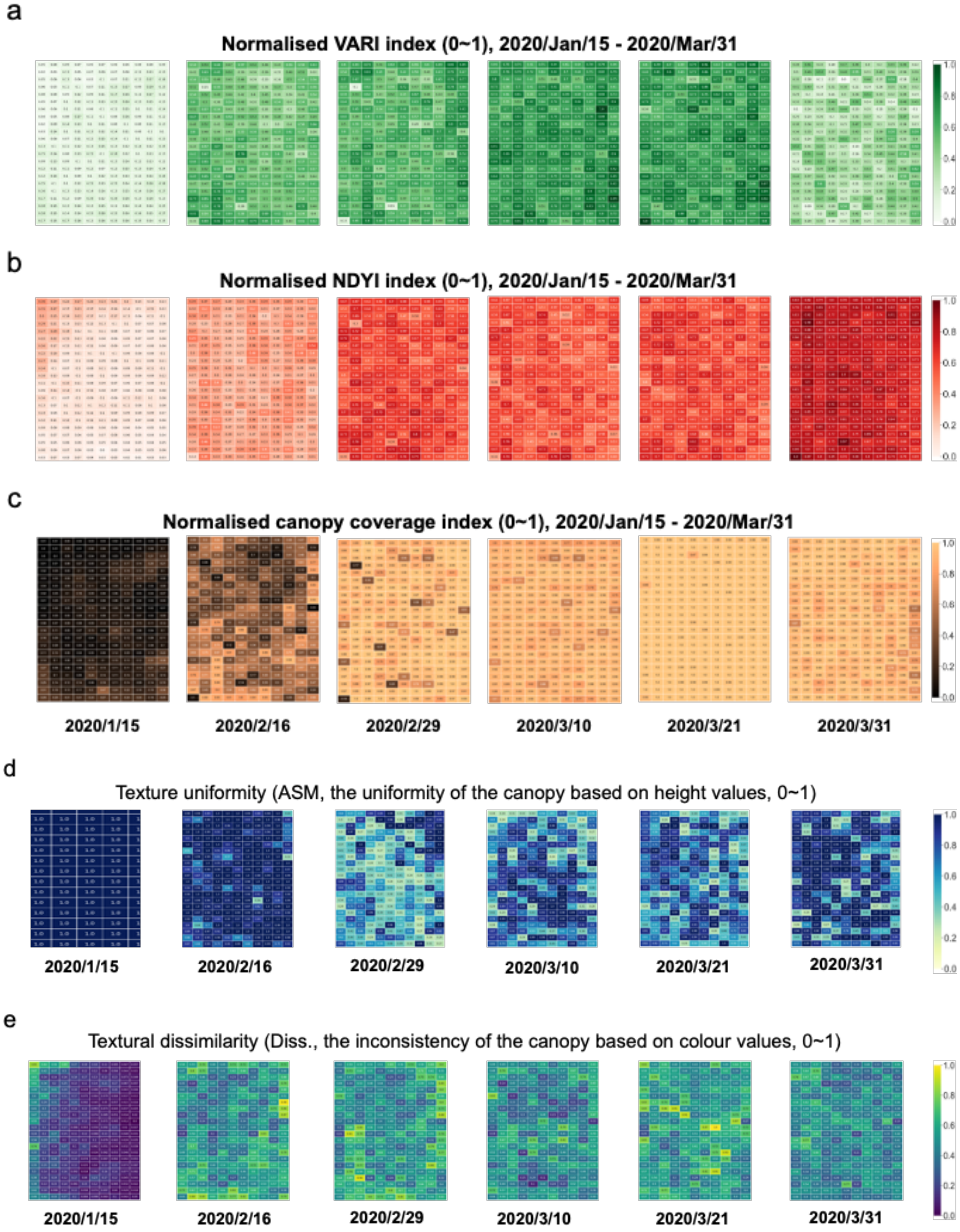

(**a**) The normalised VARI (0-1) measured for the 241 RILs, showing vegetation changes at different growth stages. (**b**) The normalised NDYI (0-1) computed for the RILs, demonstrating changes of yield potential. (**c**) The normalised canopy coverage index (0-1) scored for the RILs, showing the canopy-level development. (**d&e**) Canopy textural features derived from UAV-acquired orthomosaic images

for the 241 RILs, including texture uniformity and textural dissimilarity linking to plant lodging performance. The six time points sampled for the above measures were from 15th January 2020 to 31st March 2020. Normalised scale bars (to the right) for the five traits are shown. Abbreviations: the visible atmospherically resistant index (VARI), the normalized difference yellowness index (NDYI), canopy coverage index (CCI), angular second moment (ASM).

Fig. S5 Combining phenotypic traits and supervised machine learning to predict a complex trait, heading date, with high confidence.

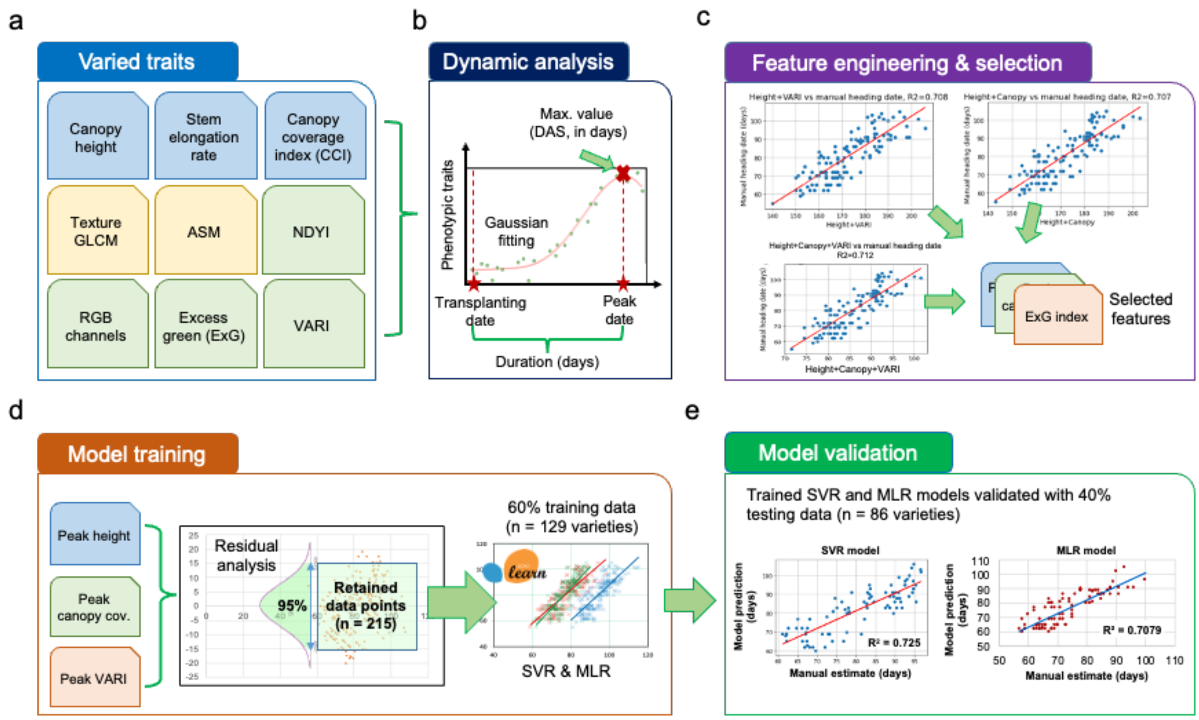

(**a**) AirMeasurer's trait analysis results were collated, containing morphological, textural, and spectral traits. (**b**) The Gaussian fitting was applied to all the traits to compute the duration (in days) between the sowing date and the dates when a given trait reached its peak value. (**c**) Feature engineering and feature selection were performed to select traits correlated with manually estimated heading dates. Three traits (i.e. height, ExG and canopy coverage index, CCI) were identified. (**d**) Residual analysis was performed to remove outliers in the manual estimate, followed by training supervised machine learning models using a training set (60%; n = 129). Both multiple linear regression (MLR) and support vector regression (SVR) models were selected due to their predictive accuracy. (**e**) Both SVR and MLR models were used to forecast the heading date trait using a testing data (40%; n = 86). SVR obtained an R^2 value of 0.725 ($p < 0.0001$) and MLR obtained an R^2 of 0.7079 ($p < 0.0001$), between the model prediction and the manual estimation.

Supporting Information Tables

Table S1 The genome-wide significant *P* value (FDR0.2).

Trait	Year	$-\log_{10}P$	*P*-value
**Max_{height} (cm), AirMeasurer**	19	6.02	9.55E-07
	20	6.25	5.62E-07
**Max. plant height (cm), manual**	19	6.73	1.86E-07
	20	6.35	4.47E-07
**Avg. CGR_{height} (%; 0 DAS – the Max_{height} day), AirMeasurer**	19	5.93	1.17E-06
	20	6.77	1.70E-07
**Avg. CGR_{height} (%; 0 DAS – the FRG_{height} day), AirMeasurer**	19	7.09	8.13E-08
	20	8.97	1.07E-09

Table S2. Genome-wide significant association (GWAS) signals of ExG and CCI related traits using EMMAx.

**Traits**	**Year**	**Chr.**	**Position[a]**	**$-\log_{10}P$**	**Distance[b] (kb)**	**Candidate genes**	**Gene Symbol**
Avg. Growth Rate (%; 0 DAS – the Max_{ExG} day), AirMeasurer	2019	1	6,853,176	6.07	303.584	Os01g0229300	*CCP1*
Avg. Growth Rate (%; 0 DAS – the Max_{CCI} day), AirMeasurer	2020	1	3,014,365	7.4	329.001	Os01g0149500	*Pit*
		6	7,877,366	6.49	223.979	Os06g0247500	*PFPβ*

Chr. Chromosome

[a] Position in bp according to IRGSP 4.0

[b] The distance between SNP and candidate gene

Supporting Information Notes S1 Trial design and plant materials

To develop a UAV-based imaging protocol that can be applied at different sites, we established two field experiments (2019-2021). The first experiment focused on landraces, with 254 varieties tested in the 2019 and 2020 seasons, including 103 *japonica*, 40 intermedia and 111 *indica* types. These landraces were reported previously (Huang *et al.*, 2012) and the trail design in Shanghai is listed in the Table 1 below. The second experiment examined 191 homozygous RILs in the 2020 and 2021 seasons (Huang *et al.*, 2010), which were derived from the crossing parents Nipponbare (*Oryza sativa ssp. Japonica*) and 93-11 (*Oryza sativa ssp. Indica*), two popular commercial varieties. The trail design in Hainan is listed in the Table 2 below.

The landraces were used to carry out GWAS analysis to find the associated-loci controlling phenotypes such as crop height, growth rate and heading date. The RIL population was used to verify AirMeasurer-measured traits such as maximum crop height through QTL mapping. Seeds of the field experiments were obtained from the National Center for Gene Research, Chinese Academy of Sciences (CAS). The GP accession numbers in the tables below can be found via the public database (http://server.ncgr.ac.cn/RiceHap2/Geno.php).

The two trial sites were chosen due to their geography and weather conditions: (1) the Chinese Academy of Sciences (CAS) Songjiang crop cultivation and breeding center (30°56’31.2”N, 121°07’19.2”E, Shanghai China) and (2) the Nanfan crop breeding center (18°31’45.12”N, 110°00’44.64”E, Lingshui, Hainan province, China). The site in Shanghai is under a humid subtropical climate, ideal for cultivating natural rice varieties due to sufficient rainfall and heat during the summer, whereas the Hainan site is under a tropical climate, suitable for multi-generation crop production and rapid generation cycling to improve genetic populations in rice. Crops at both sites were managed using standard husbandry and agronomic inputs according to local conditions. To verify and improve the UAV-based phenotyping with the multi-season case study, in Shanghai, rice landraces were sown in 2 x 1.1 m plots (18 plants per plot) in June 2019 and 2020; in Hainan, RILs were sown in 2 x 1.1 m plots (18 plants per plot) in December 2019 and 2020.

1. The trial design and rice landraces studied in the 2019 and 2020 seasons in Shanghai.

**Accession**	**Accession**	**Accession**	**Accession**	**Accession**	**Accession**	**Accession**	**Accession**	**Accession**	**Accession**	**Accession**	**Accession**	**Accession**	**Accession**	**Accession**
GP015	GP014	GP013	GP012	GP011	GP010	GP009	GP008	GP007	GP006	GP005	GP004	GP003	GP002	GP001
GP030	GP029	GP028	GP027	GP026	GP025	GP024	GP023	GP022	GP021	GP020	GP016	GP018	GP017	GP019
GP031	GP032	GP033	GP034	GP035	GP036	GP037	GP038	GP039	GP040	GP041	GP042	GP043	GP044	GP045
GP060	GP059	GP058	GP057	GP056	GP055	GP054	GP053	GP052	GP051	GP050	GP049	GP048	GP047	GP046
GP061	GP062	GP063	GP064	GP065	GP066	GP067	GP068	GP069	GP070	GP071	GP072	GP073	GP074	GP075
GP091	GP090	GP089	GP087	GP086	GP085	GP084	GP083	GP082	GP081	GP080	GP079	GP078	GP077	GP076
GP092	GP093	GP094	GP095	GP096	GP097	GP098	GP099	GP100	GP101	GP102	GP103	GP104	GP105	GP106
GP121	GP120	GP119	GP118	GP117	GP116	GP115	GP114	GP113	GP112	GP111	GP110	GP109	GP108	GP107
GP122	GP123	GP124	GP125	GP126	GP127	GP128	GP129	GP130	GP131	GP132	GP133	GP134	GP135	GP136
GP503	GP502	GP501	GP500	GP499	GP498	GP497	GP144	GP143	GP142	GP141	GP140	GP139	GP138	GP137
GP504	GP505	GP506	GP507	GP508	GP509	GP510	GP511	GP512	GP513	GP514	GP515	GP516	GP517	GP518
GP535	GP533	GP532	GP531	GP530	GP529	GP528	GP527	GP526	GP525	GP524	GP523	GP522	GP521	GP520
GP536	GP537	GP538	GP539	GP540	GP541	GP542	GP543	GP544	GP545	GP546	GP547	GP548	GP549	GP551
GP566	GP565	GP564	GP563	GP562	GP561	GP560	GP559	GP558	GP557	GP556	GP555	GP554	GP553	GP552
GP567	GP568	GP569	GP570	GP571	GP572	GP573	GP574	GP575	GP576	GP577	GP578	GP579	GP580	GP581
GP596	GP595	GP594	GP593	GP592	GP591	GP590	GP589	GP588	GP587	GP586	GP585	GP584	GP583	GP582
GP597	GP598	GP599	GP601	GP602	GP603	GP605	GP606	GP607	GP608	GP609	GP610	GP611	GP612	GP613
GP629	GP628	GP627	GP626	GP625	GP624	GP623	GP622	GP621	GP620	GP619	GP617	GP616	GP615	GP614

2. The trial design and rice RILs studied in the 2020 and 2021 seasons in Hainan.

**Accession**	**Accession**	**Accession**	**Accession**	**Accession**	**Accession**	**Accession**	**Accession**	**Accession**
R162	R161	R117	R116	R072	R071	R027	R026	HP598
R163	R160	R118	R115	R073	R070	R028	R025	HP599
R164	R159	R119	R114	R074	R069	R029	R024	HP600
R165	R158	R120	R113	R075	R068	R030	R023	HP601
R166	R157	R121	R112	R076	R067	R031	R022	HP602
R167	R156	R122	R111	R077	R066	R032	R021	HP603
R168	R155	R123	R110	R078	R065	R033	R020	HP604

R169	R154	R124	R109	R079	R064	R034	R019	HP606
R170	R153	R125	R108	R080	R063	R035	R018	HP607
R171	R152	R126	R107	R081	R061	R036	R017	HP608
R172	R151	R127	R106	R082	R060	R037	R016	HP609
R173	R150	R128	R105	R083	R059	R038	R015	HP610
R174	R149	R129	R104	R084	R058	R039	R014	HP611
R175	R148	R130	R103	R085	R057	R040	R013	HP612
R176	R147	R131	R102	R086	R056	R041	R012	HP613
R177	R146	R132	R101	R087	R055	R042	R011	HP614
R178	R145	R133	R100	R088	R054	R043	R010	9311
R179	R143	R134	R099	R089	R053	R044	R009	NIP
R180	R142	R135	R098	R090	R052	R045	R008	R001
R181	R141	R136	R096	R091	R051	R046	R007	R002
R182	R140	R137	R095	R092	R050	R047	R006	R003
R183	R139	R138	R094	R093	R049	R048	R005	R004

Supporting Information Notes S2 – UAV imaging protocol and in-field setups

2.1 Mission plans

To set up a new project for conduct UAV imaging for cereal crops, users could use Pix4D or DJI flight control software. Here, we used Pix4DCapture to explain the procedure of setting up UAV imaging. For projects to reconstruct 3D point clouds, users need to select "Double Grid Mission" to plan the UAV phenotyping. For projects that only required 2D orthomosaics, user need to select "Grid Mission" (**Fig. S2.1**).

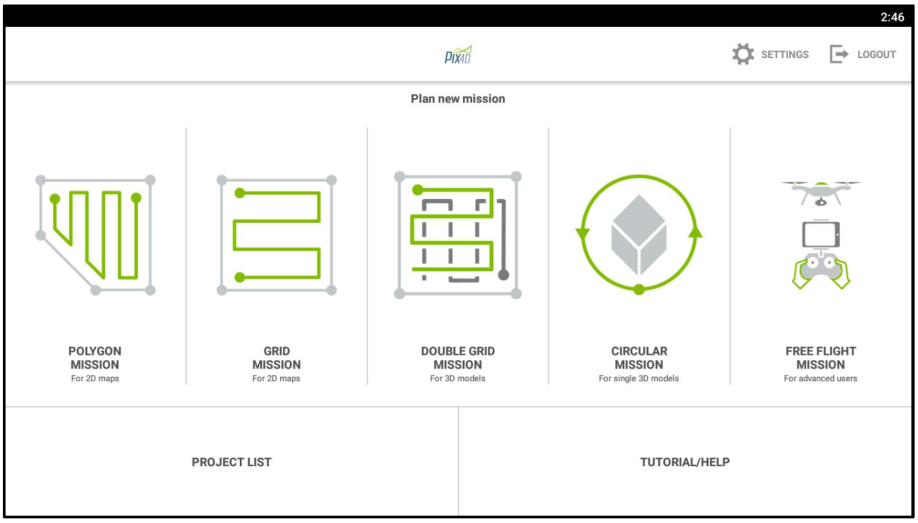

Figure S2.1 Plan UAV imaging mission

A GPS-tagged map will then open showing the current location and a mission plan (**Fig. S2.2**). It is important to ensure that the mobile phone or the tablet used for planning UAV flights are connected with the network, otherwise only the cached map will be displayed at this stage.

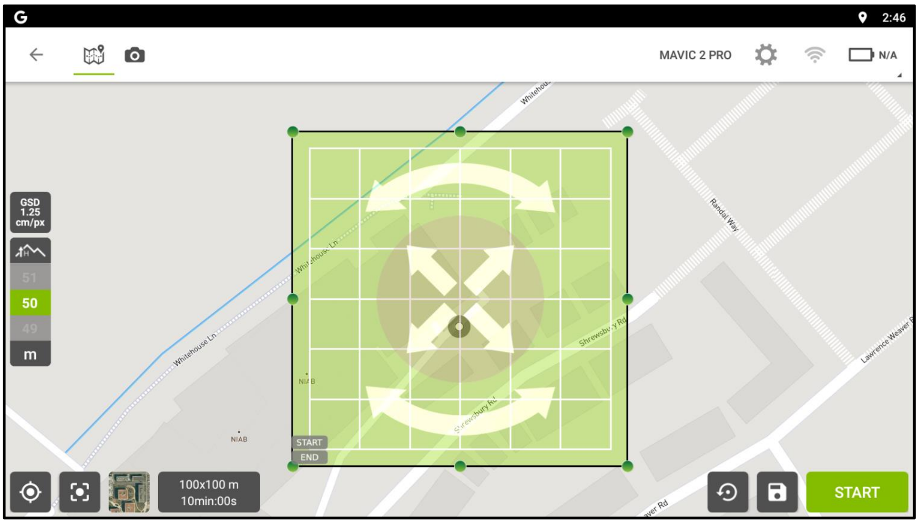

Figure S2.2 Plan aerial imaging region

Users could drag the mission into rough place using the central drag controls, the rotation controls and resizing anchors. When planning the UAV imaging for the first time, we recommend pilots and plant researchers to walk in the experimental field to accurately position the UAV mission with the controller as well as the ground control points (GCPs), so that the experimental region, GCPs and other geo-referencing points could be verified (**Fig. S2.3**).

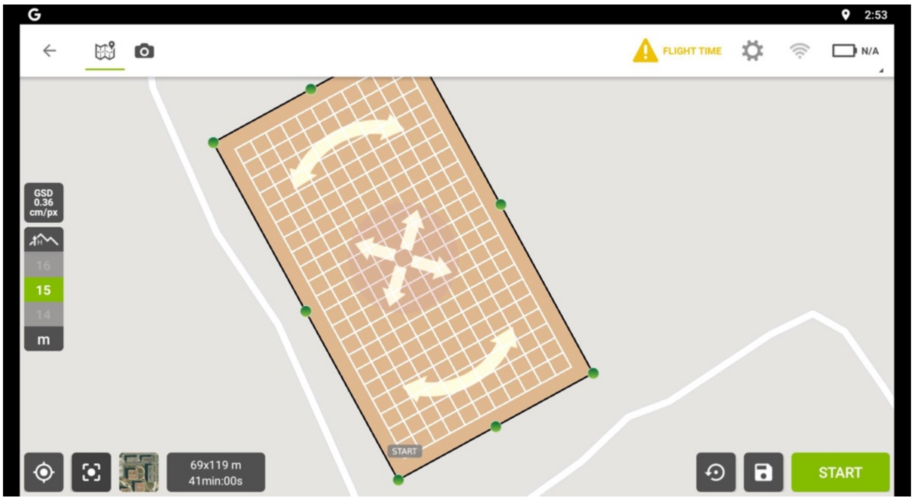

Figure S2.3 Position the imaging region according to the experimental field

2.2 In-field setting and imaging protocols

We recommend placing a reference point in each corner of the experimental field to help geo-reference the experimental area in the data pre-processing. If the slope of the field is bigger than the height of the crops and 3D reconstruction is required, it is useful to place the fifth GCP in the centre of the field and another height reference point randomly in the field (**Fig. S2.4**).

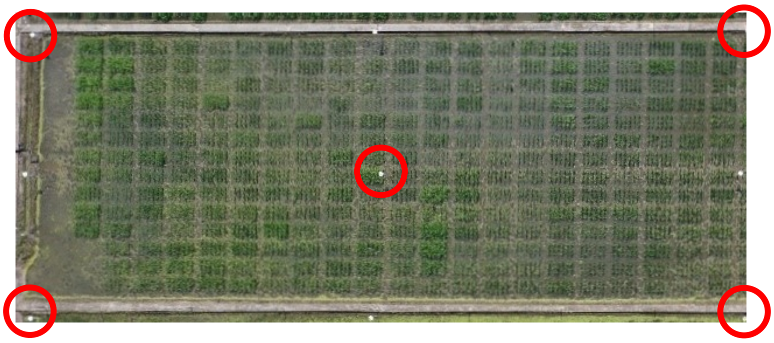

Figure S2.4 Installed GCPs and height references in the field

By positioning the points in opposite corners (i.e. top-right and bottom-left), users can adjust the mission area. We recommend the mission area should be slightly bigger than the experimental field so that the edges of the field could be covered sufficiently (**Fig. S2.5**). For more accurate geo-referencing results, users shall use real-time kinematic (RTK) positioning. For example, by placing the RTK antenna in the middle of a given GCP and record its exact location, the recorded geo-coordinates could

be recorded and then used for data pre-processing, calibrating 3D point clouds and defining region of interest (ROI) of the experimental area.

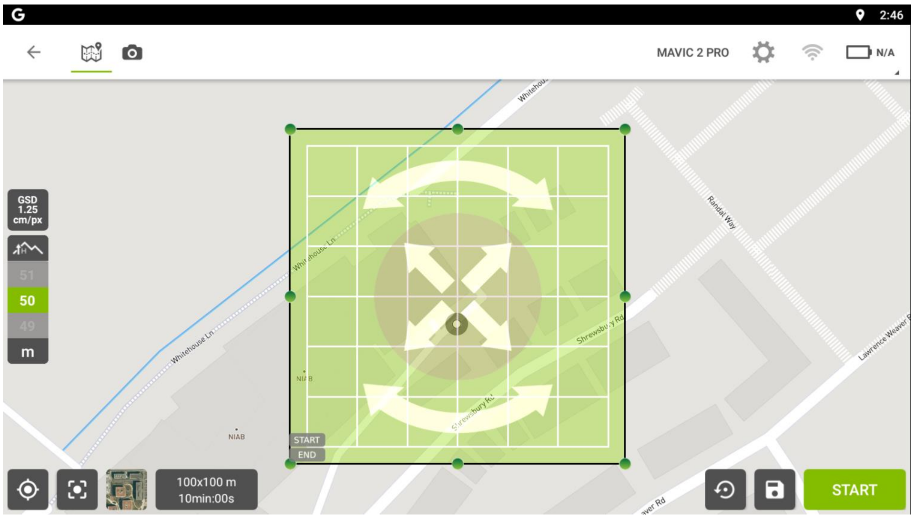

Figure S2.5 Adjust mapping area based on GCPs in the field

The position of the controller shows on the map as a white dot in a grey circle. We recommend using the white-dot position to define the mapping area (**Fig. S2.6**), then reshaping until the position of the controller is in the corresponding corner, but within the flight paths (i.e. white lines).

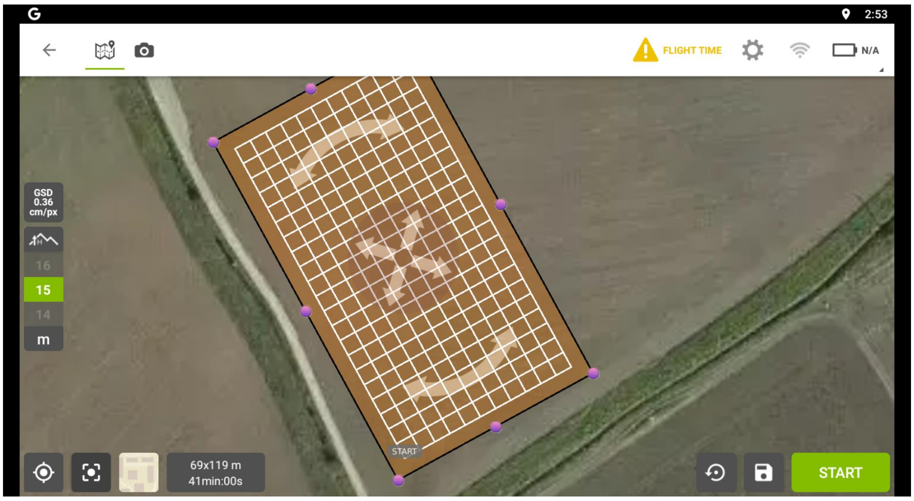

Figure S2.6 Adjust mapping area using the controller in the field

Set the flying altitude of the imaging mission on the left side of the screen (e.g. **Fig. S2.7** shows an imaging height of 15m). To facilitate high-quality 3D reconstruction, lower altitude is recommended. For example, we flew 8-10 metres if we wanted to acquire detailed spectral information of the crops. If the imaging purpose is to generate 2D orthomosaic, we normally flew 30-35 metres to accomplish imaging within 10 mins. It is important to note that any UAV should not be flown in restricted areas and no closer than 5 metre to overhead electrical wires.

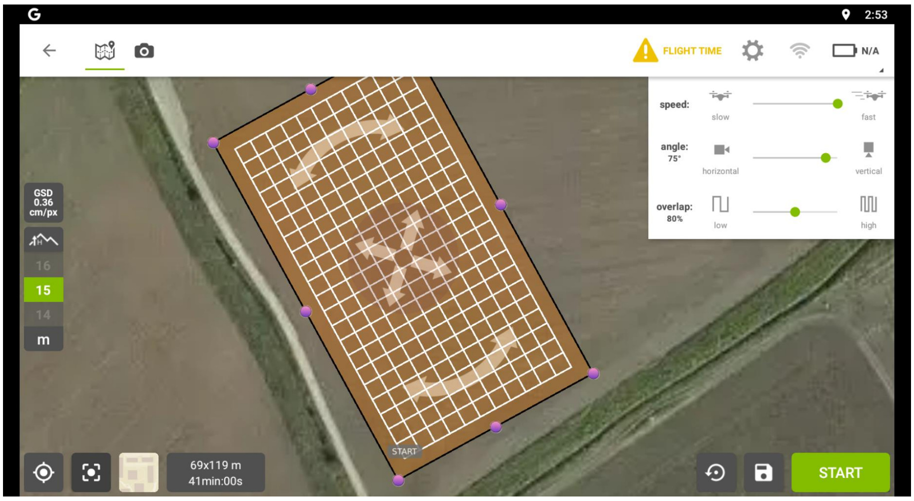

Figure S2.7 Set up flight and imaging parameters for the field experiment

Detailed flight parameters can be set by tapping the ‘Cog’ icon in the top-right of the screen for the flight mission. We normally set drone speed lower than 2 metre per second, camera angle 75-80°, image overlap forward 80% and side 75%. We need to check the estimated flight time of the mission at the bottom of the screen (**Fig. S2.7**). If the time is over 20 minutes (airtime for one battery is around 20-25 minutes), the flying task needs to be split into multiple missions.

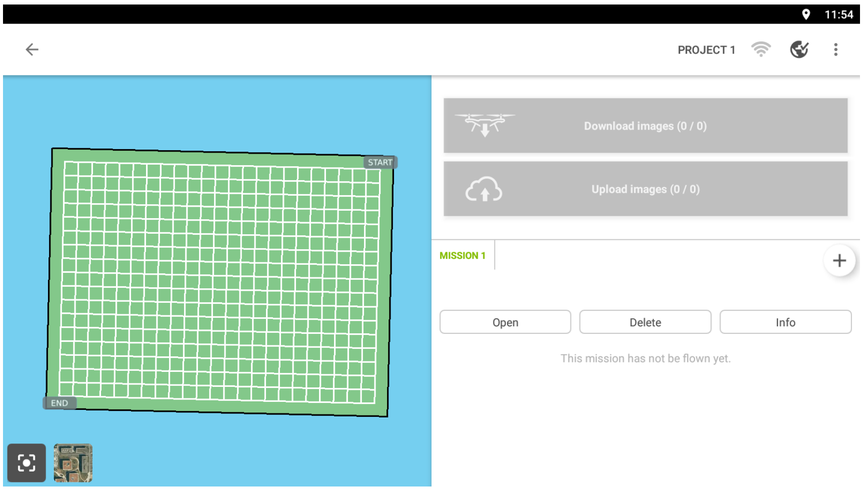

Figure S2.8 Set up flight projects

Users need to tap the floppy disc icon in the bottom-right of the screen (next to the START button, **Fig. S2.7**). By tapping the ‘+’ button on the right side of the screen (below Upload Images in **Fig. 2.8**), users could add a new mission by copying the current project. Then, users need to select the Double Grid Mission again. In the newly opened flight mission setup screen, users could resize the initially defined mission area and set up another mapping area to ensure that the estimated airtime for both mappings are less than 20 minutes (**Fig. S2.9**). The process could be repeated, and multiple missions could be added to the flight project. The last white line of the previous mission should be overlapped with the first white line of this mission.

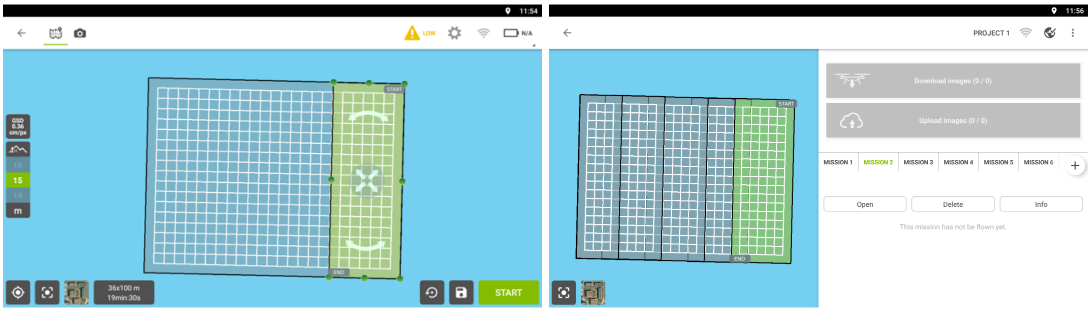

Figure S2.9 Set up another flight mission for the experimental field

Finally, users can tap the save icon to return to the mission select screen and then select the first of the smaller missions (Usually Mission 2) then tap 'open' to run this mission. Once this mission has opened, users can press 'take off' and the UAV will be flown automatically following the above project.

2.3 Pix4D mapper setting

Open Pix4D Mapper and start a new Pix4D project. The protocol here is to set an output folder called "Pix4D" to host all the data pre-processing results. By clicking "Add Images" button, users could select a series of UAV-collected images to produce 2D orthomosaic, 3D point cloud images, and browse the dataset directory to set up the coordinate system (e.g. automated detection; **Fig. S2.10**).

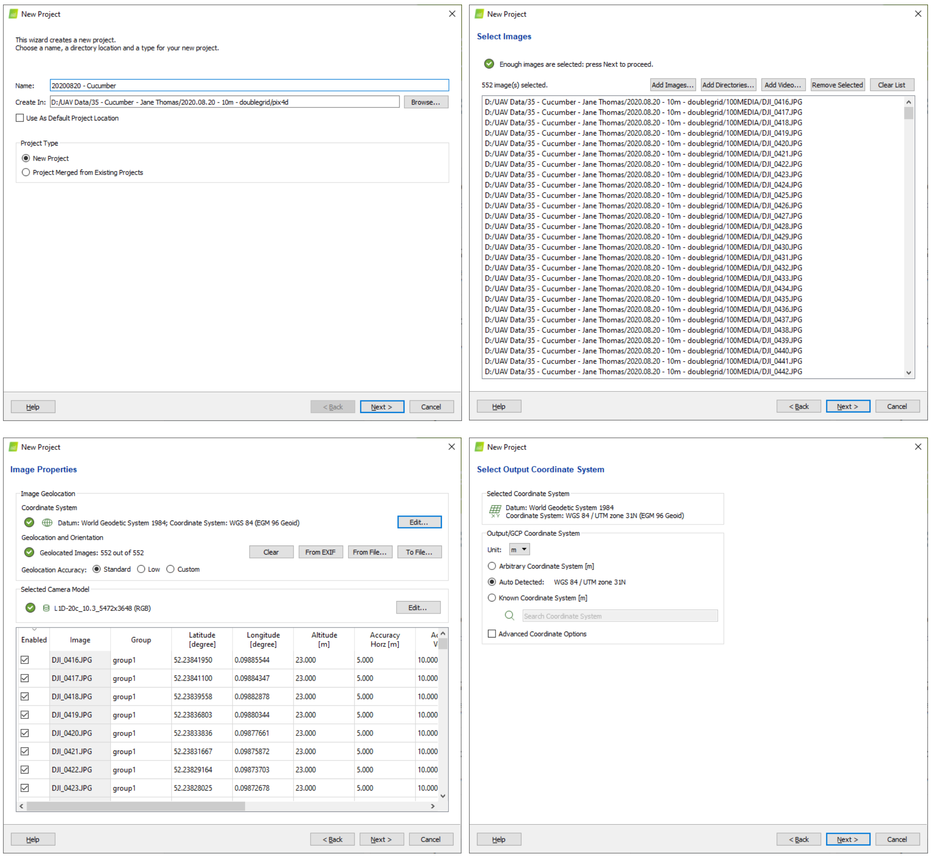

Figure S2.10 Select UAV image series to process

2.4 Processing setting protocols and geo-referencing

To generate 2D orthomosaics and 3D point clouds, users need to accomplish three steps of processing setting in Pix4D Mapper, including:

1) *Initial processing*, for which users need to set up key points image scale (custom, ½ image size), generate quality report, matching image pairs (free flight or terrestrial) with geometrically verified matching, standard calibration mode and rematch mode.
2) *Point cloud and mesh*, for which users need to set up image scale (1/2 half image size with the multiscale selection), minimum number of matches (set to 3), optimal point density, 3D textured mesh (no textured mesh), medium resolution of point clouds, matching window (9x9 pixels) with processing area and annotations, and export file formats (LAS and PLY).
3) *DSM, orthomosaic and index*, for which users need to set up resolution (automatic, 1 GSD), orthomosaic (GeoTIFF, without transparency), and not to generate DSM and DTM through Pix4D Mapper as the Pix4D software could not remove the slope and terrain features in relatively small crop fields.

After setting the processing parameters, we recommend users to only select the initial processing step (i.e. without selecting Step 2 "Point cloud and mesh" and Step 3 "DSM, orthomosaic and index") to start processing (**Fig. S2.11**). The reason for this is because we need to assess the Quality Report as well as to geo-reference UAV images before spending many hours to process the next two steps.

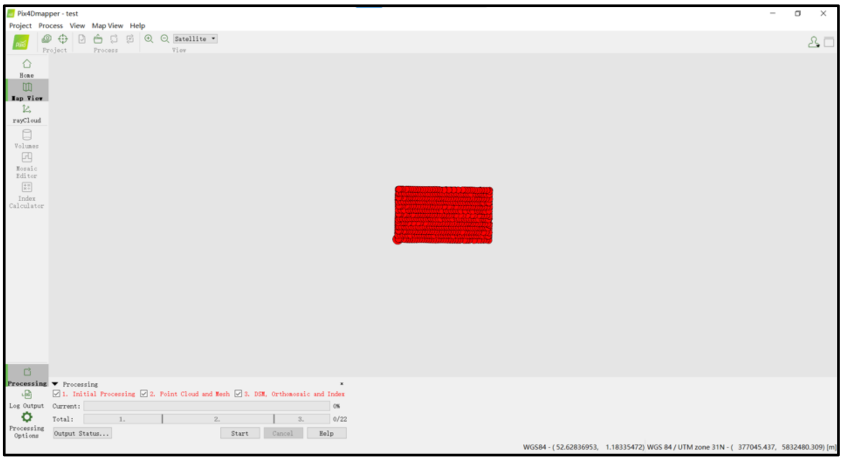

Figure S2.11 Select UAV image series to perform the initial processing

2.5 Geo-referencing ground control points

2.5.1 Georeferencing with RTK information

Please note that this step is needed if RTK information was recorded from the GCPs. Users need to open "GCP/MTP Manager", through which RTK files could be imported to Pix4D Mapper. Then, users

shall click the Basic Editor to associate GCPs with RTK recorded geo-coordinates, which requires users to label the central point of a given GCP in 8-10 images (**Fig. S2.12**).

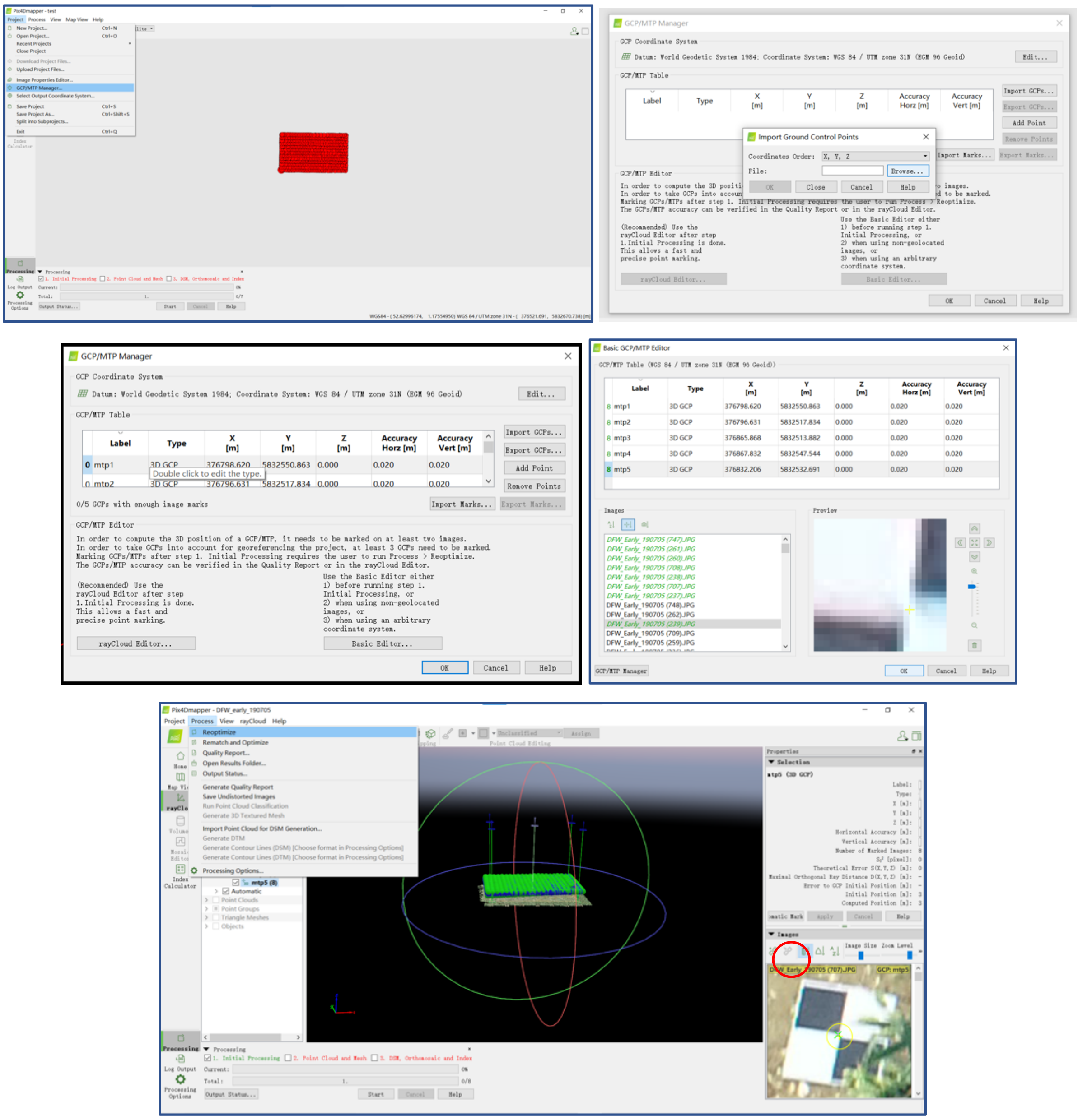

Figure S2.12 Associate geo-coordinates with a given GCP

After associating geo-coordinates with all the GCPs in the field, users shall click “Reoptimize” button in the Process submenu to apply the rectification of geo-referencing of all UAV images. After that, users need to regenerate the Quality Report and then proceed to “DSM, Orthomosaic and Index” and “Point Cloud and Mesh” steps. The 3D reconstruction results before and after geo-referencing UAV images can be seen in the figure below (**Fig. S2.13**).

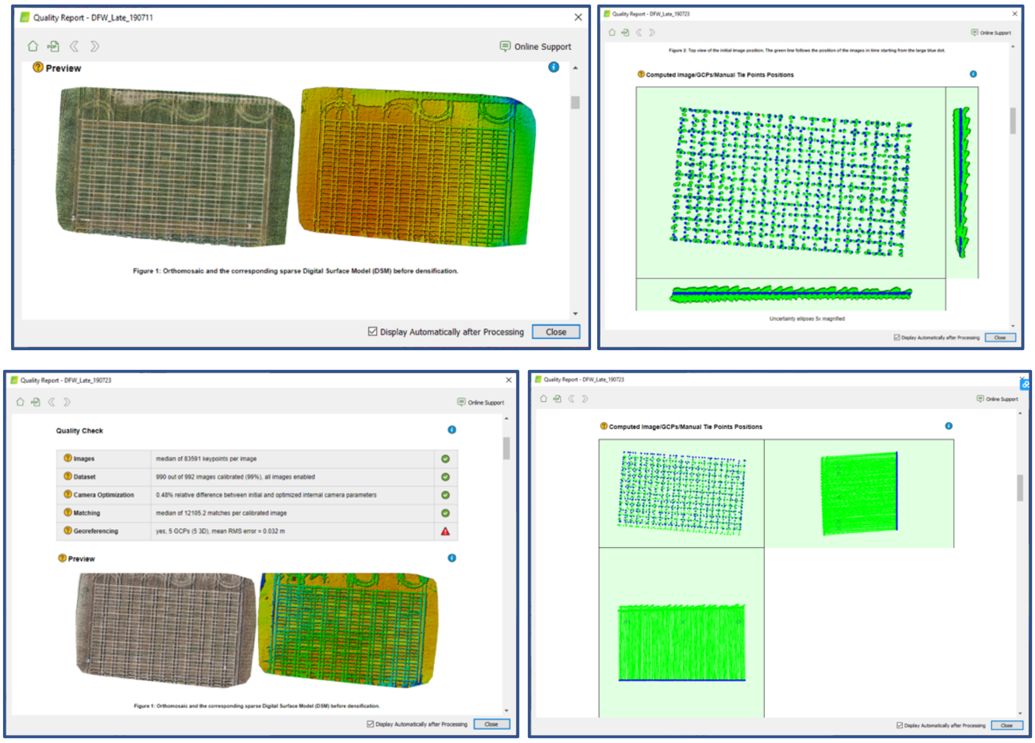

Figure S2.13 The results of Pix4D 3D reconstruction with and without geo-referencing

2.5.2 Georeferencing without RTK information

To geo-reference UAV images without RTK information, we used CloudCompare to measure the 3D coordinates in the 3D scene. First, we opened CloudCompare and clicked "Open File" to open a point cloud file (in PLY format as no RTK information associated) produced by Pix4D (**Fig. S2.14**).

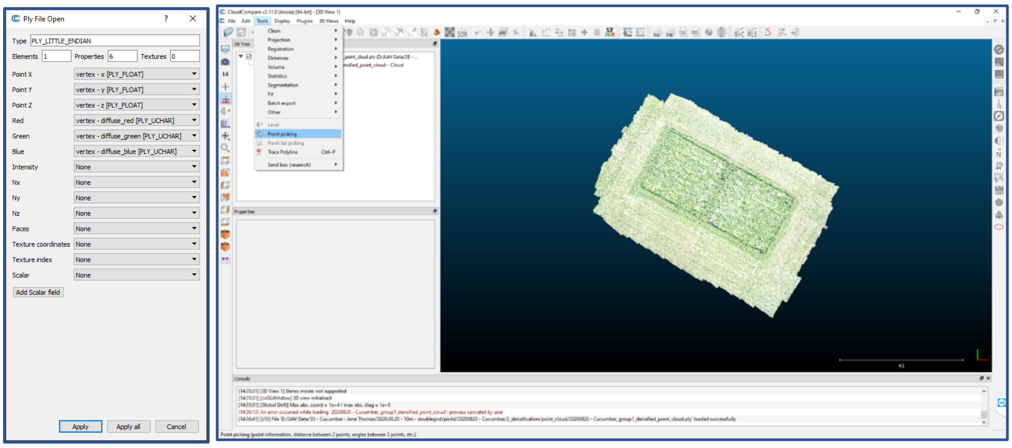

Figure S2.14 Open 3D point clouds in CloudCompare

Then, we clicked "Tools>Point picking" from the top bar to locate the GCPs in the 3D points. By left clicking in the middle of a GCP, we could find its relative 3D coordinates (**Fig. S2.15**). In particular, we record the height reference in the point clouds using this method, so that the actual height value could be associated with the 3D coordinates in the 3D scene. For example, the height reference in the figure below is 1.5m in height.

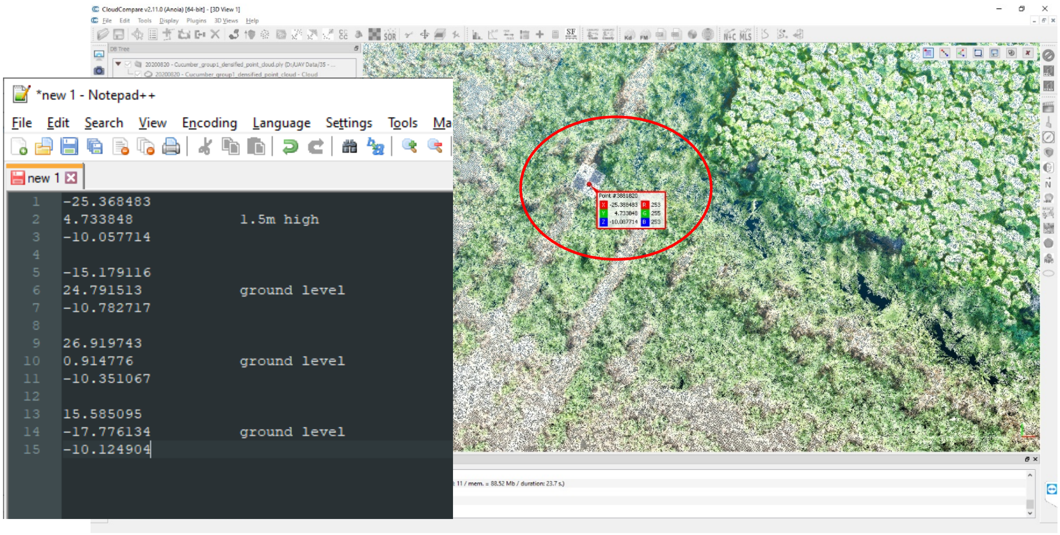

Figure S2.15 Measure 3D coordinates using GCPs and height reference installed in the field

After recording all GCPs and height references in the field, we will need to reconfigure the point clouds. Similar to the RTK approach, we also use Pix4D's "GCP/MTP Manager". By clicking "Add Point" for each GCP, we could edit the coordinate system using the "Arbitrary Coordinate System" based on the pseudo 3D coordinates recorded from CloudCompare (**Fig. S2.16**).

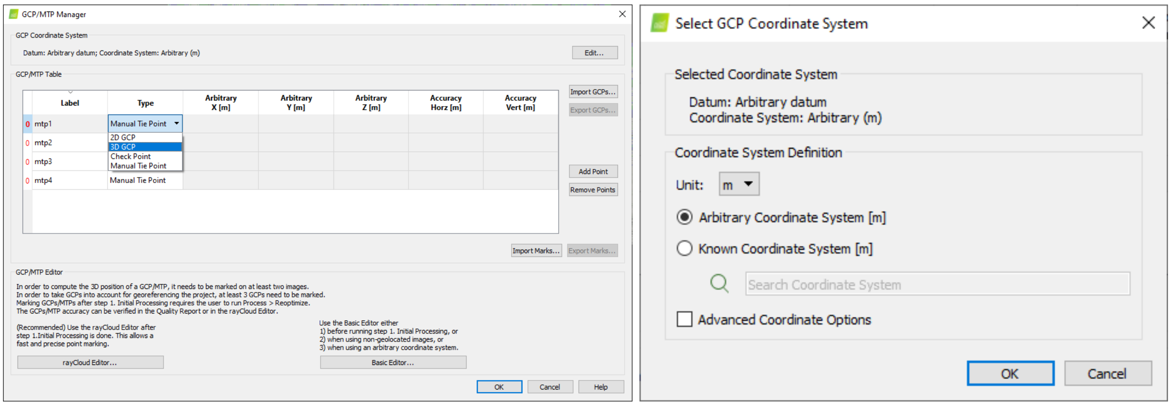

Figure S2.16 Reconfigure 3D coordinates for the crop field in Pix4D Mapper

For each control point, we could enter the 3D coordinates recorded from CloudCompare or the RTK device. We set the 'Z' value on all points to '0' for all the GCPs with the exception of the height reference point, which needs to be set as 1.5m tall. (NOTE: different coordinate systems might use the y- and z-axis differently; for example, the height values below were set in y-axis). If users are using RTK coordinates, users need to double-check the geo-coordinates (e.g. WGS84) they used. Similar to RTK georeferencing, users need to repeat the tagging procedure for 8-10 images for each GCP. Then, users need to "Recalculate" with the reconfigured coordinates.

Supporting Information Notes S3 – Different plant height measures before and after removing terrain features

3.1 Before unwanted terrain features removed

Correlation analysis (the highest square of the correlation coefficient, R^2) between manual scored maximum plant height and AirMeasurer-derived canopy height using 3D point clouds before removing unwanted terrain features (**Fig. S3.1**).

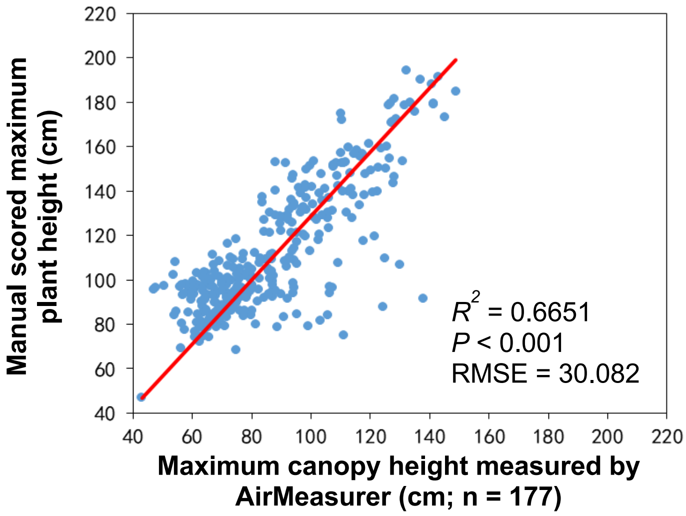

Figure S3.1 Correlation analysis between manual scored and AirMeasurer-derived maximum canopy height using 3D point clouds before removing unwanted terrain features

3.2 After unwanted terrain features removed

Correlation analysis (R^2) between manual scored maximum plant height and AirMeasurer-derived canopy height using 3D point clouds after removing unwanted terrain features (**Fig. S3.2**).

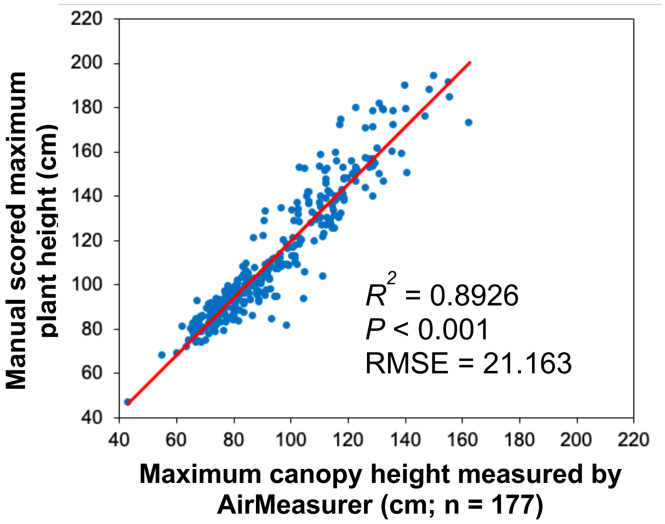

Figure S3.2 Correlation analysis between manual scored and AirMeasurer-derived maximum canopy height using 3D point clouds after removing unwanted terrain features

Supporting Information Notes S4 – 3D point clouds processing and canopy height model

```
# The function to generate CHM from calibrarted 3D point clouds
def make_chm(point_cloud_dir,poly_path):
    test_dir=os.path.abspath(os.path.dirname(point_cloud_dir))
     try:
         # Create an intermediate procedure folder
        os.mkdir(test_dir+'\\'+'process')
        pro_dir=test_dir+'\\'+'process'
        os.mkdir(test_dir+'\\'+'mask') # Create a mask folder
        mask_dir=test_dir+'\\'+'mask'
        os.mkdir(test_dir+'\\'+'result') # Create a result folder
        result_dir = test_dir+'\\'+'result'
        os.mkdir(test_dir+'\\'+'chm')
        chm_dir = test_dir+'\\'+'chm'
    except FileExistsError:
        pro_dir=test_dir+'\\'+'process'
        mask_dir=test_dir+'\\'+'mask'
        result_dir = test_dir+'/'+'result'
        chm_dir = test_dir+'\\'+'chm'

    wbt = whitebox.WhiteboxTools()
    wbt.set_verbose_mode(False)
    las_path = os.listdir(point_cloud_dir)
    for name in tqdm(range(len(las_path))):
        lasfile = point_cloud_dir+'\\'+las_path[name]
        inFile = laspy.file.File(lasfile, mode = "r")
        x,y,z = inFile.x,inFile.y,inFile.z

        # Denoising
        lasdata = zip(x,y,z)
        tree = spatial.cKDTree(list(lasdata))
        sigma=10
        K=50
        k_dist=np.zeros_like(x)
        for i in range(len(x)):
            dist,index =tree.query(np.array([x[i],y[i],z[i]]), K)
            k_dist[i] = np.sum(dist)
        max_distance = np.mean(k_dist) + sigma*np.std(k_dist)
        outer_index=np.where(k_dist>max_distance)
        sor_filter = k_dist<=max_distance
        outFile = laspy.file.File(pro_dir+'\\'+'sor_'+las_path[name],
                          mode='w', header=inFile.header)
        outFile.points = inFile.points[sor_filter]
        outFile.close()
        inFile.close()

        # Classified ground point
        inFile_sor = laspy.file.File(pro_dir+'\\'+'sor_'+las_path[name], mode='r')
        points_sor = inFile_sor.points
        xyz_sor = np.vstack((inFile_sor.x, inFile_sor.y, inFile_sor.z)).transpose()
        csf = CSF.CSF()
        csf.params.bSloopSmooth = False
        csf.params.cloth_resolution = 1
        csf.params.class_threshold =0.01
        csf.setPointCloud(xyz_sor)
        ground = CSF.VecInt()
        non_ground = CSF.VecInt()
        csf.do_filtering(ground, non_ground)
        outFile_ground = laspy.file.File(pro_dir+'\\'+'ground'+las_path[name],
                              mode='w', header=inFile_sor.header)
        outFile_ground.points = points_sor[ground]
        outFile_ground.close()
        inFile_sor.close()

        # Produce CHM
        lasfile_ground = pro_dir+'\\'+'ground'+las_path[name]
        inFile_ground = laspy.file.File(lasfile_ground, mode = "r")
        x_ground,y_ground,z_ground = inFile_ground.x,inFile_ground.y,
                                     inFile_ground.z
        inFile_ground.close()

        wbt.lidar_elevation_slice(
            i=pro_dir+'\\'+'sor_'+las_path[name],
            output=pro_dir+'\\'+'sor_above_'+las_path[name],
            minz=z_ground.min(),
```

```
    maxz=None,
    cls=False
)

wbt.lidar_nearest_neighbour_gridding(
    i= pro_dir+'\\'+'sor_above_'+las_path[name],
    output= pro_dir+'\\'+'sor_above_'+
              os.path.splitext(las_path[name])[0]+'.tif',
    parameter="elevation",
    returns="all",
    resolution=0.01,
    radius=0.01,
    exclude_cls=None,
    minz=None,
    maxz=None
)

wbt.lidar_tin_gridding(
    i= pro_dir+'\\'+'ground'+las_path[name],
    output= pro_dir+'\\'+'ground'+
              os.path.splitext(las_path[name])[0]+'.tif',
    parameter="elevation",
    returns="all",
    resolution=0.01,
    exclude_cls=None,
    minz=None,
    maxz=None,
    max_triangle_edge_length=None
)

wbt.clip_raster_to_polygon(
    i= pro_dir+'\\'+'sor_above_'+
              os.path.splitext(las_path[name])[0] +'.tif',
    polygons= poly_path,
    output= pro_dir+'\\'+'sor_above_roi_'+
              os.path.splitext(las_path[name])[0]+'.tif'
)

wbt.clip_raster_to_polygon(
    i= pro_dir+'\\'+'ground'+os.path.splitext(las_path[name])[0]+'.tif',
    polygons=poly_path,
    output= pro_dir+'\\'+'ground_roi'+
              os.path.splitext(las_path[name])[0]+'.tif'
)

dtm = pro_dir+'\\'+'ground_roi'+
       os.path.splitext(las_path[name])[0]+'.tif'

with rasterio.open(dtm) as src:
    dem_im = src.read(1, masked=True)
    ext = rasterio.plot.plotting_extent(src)

dsm = pro_dir+'\\'+'sor_above_roi_'+
       os.path.splitext(las_path[name])[0]+'.tif'

with rasterio.open(dsm) as src:
    dsm_im = src.read(1, masked=True)
    dsm_meta = src.profile

chm = dsm_im - dem_im

nodatavalue = chm.min()
chm_fi = np.ma.filled(chm, fill_value=nodatavalue)

chm_meta = dsm_meta.copy()
chm_meta.update({'nodata': nodatavalue})

with rasterio.open(chm_dir+'\\'+'chm'+
       os.path.splitext(las_path[name])[0]+'.tif', 'w', **chm_meta) as ff:
    ff.write(chm_fi,1)
```

Supporting Information Notes S5 – Previous published segmentation solutions trialed in rice field experiments

Existing solutions relied on manual inputs to define plot layouts and adjust parameters for specific species/varieties; some employed algorithms (e.g. K-means) that were computationally expensive; and, many solutions largely relied on spectral or textural signals to distinguish plots from surrounding signals (e.g. wheelings between plots).

5.1 Plot segmentation using RGB- or texture-based (entropy) signals

Before developing our plot segmentation algorithm, we have trialed a range of approaches to segment plots from 2D orthomosaic images. For example, we used red-green-blue (RGB) and texture entropy-based images to build the segmentation algorithm. In general, the use of RGB images and the use of canopy height model (CHM) were similar. However, the RGB-based method required a clear plot boundary between plots (e.g. pixel clusters with different colour properties), which was difficult in rice experimental fields due to changing water levels (varied plot size) and weedy/volunteer plants (e.g. green duckweeds) disturbing the separation largely based on colour signals (Holman *et al.*, 2019). The RGB-based plot segmentation approach is relatively easy to establish for dryland crops such as wheat and Maize, particularly before lodging appeared; however, for rice experiments, this was proved to be difficult due to connected patches of duckweeds commonly appeared in rice paddy fields (**Fig. S5.1**).

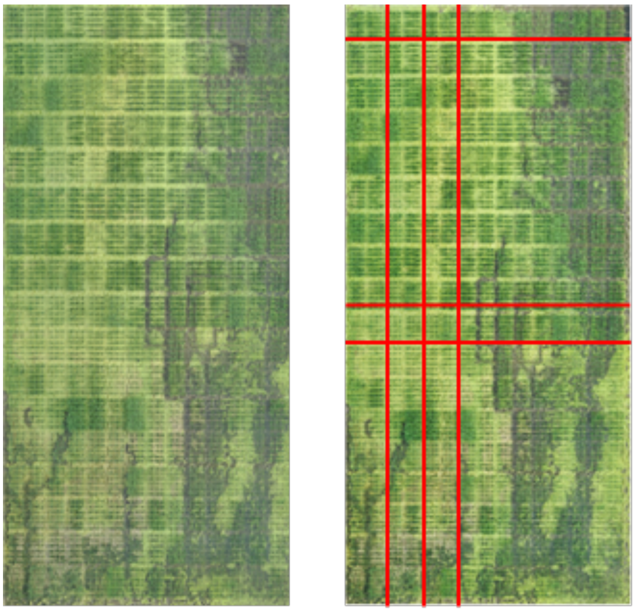

Figure S5.1 Plot segmentation result using RGB-based signals (53 DAS)

Similarly, texture signal is a visible feature that reflects the homogeneous phenomenon in an image, which can be used to demonstrate the periodically changing crop canopy surface using structural arrangement properties (Öztürk & Akdemir, 2018). However, texture signals and their derived measures (e.g. gray-level co-occurrence matrix, GLCM) are insufficient to differentiate between rice canopy surface and adjacent plot boundaries because textural and spatial information are not related

(**Fig. S5.2**). In addition, we found that texture features were affected by nature illumination and water reflection in rice fields. Hence, this led to our solution introduced in the article, which combined CHM with 2D orthomosaic image to perform plot segmentation as duckweed was lower than rice seedlings in the field.

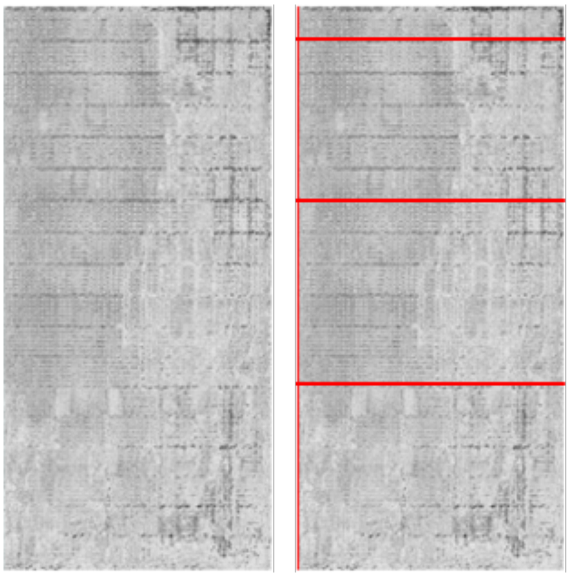

Figure S5.2 Plot segmentation result using textural signals (i.e. entropy feature extracted from the GLCM; 53 DAS)

5.2 Plot segmentation using Easy MPE

Easy MPE (Tresch *et al.*, 2019) uses Excess Green (ExG) to distinguish soil and plants, based on which plots are divided. In a rice paddy field, ExG signals (**Fig. S5.3a**) could not be adequately employed to recognise plot boundaries due to many connected duckweeds at early establishment phase (e.g. 21 DAS; **Fig. S5.3b**). In addition, Easy MPE took around 40 minutes to generate the plot mask on an ordinary Windows computer (intel i7 CPU, 16 GB RAM, and integrated UHD 630 Graphics); whereas AirMeasurer finished the same task within 15 minutes, including denoising, ground-level classification, CHM generation and plot segmentation.

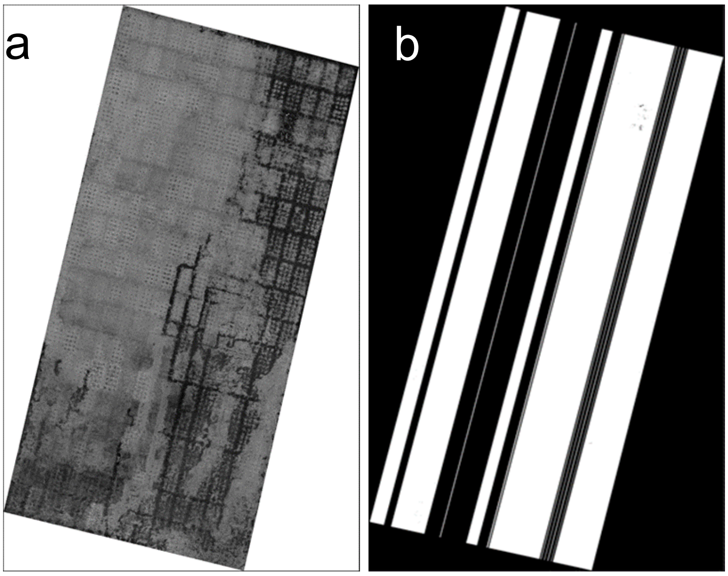

Figure S5.3 Plot segmentation results using Easy MPE (21 DAS)

(**a**) An excess green (ExG) image obtained using Easy MPE. (**b**) Plot segmentation using Easy MPE.

5.3 Plot segmentation using GRID

GRID (Chen & Zhang, 2020) utilises the K-means algorithm to cluster colour information in RGB images. Still, even for growth stages that the impacts of duckweeds were limited (e.g. 53 DAS; **Fig. S5.4a**), k-means performed reasonably satisfactorily (**Fig. S5.4b**), many plots were clustered together due to similar RGB representation, which could not be rectified in the software. Also, GRID took close to 3o minutes to generate the analysis results listed below.

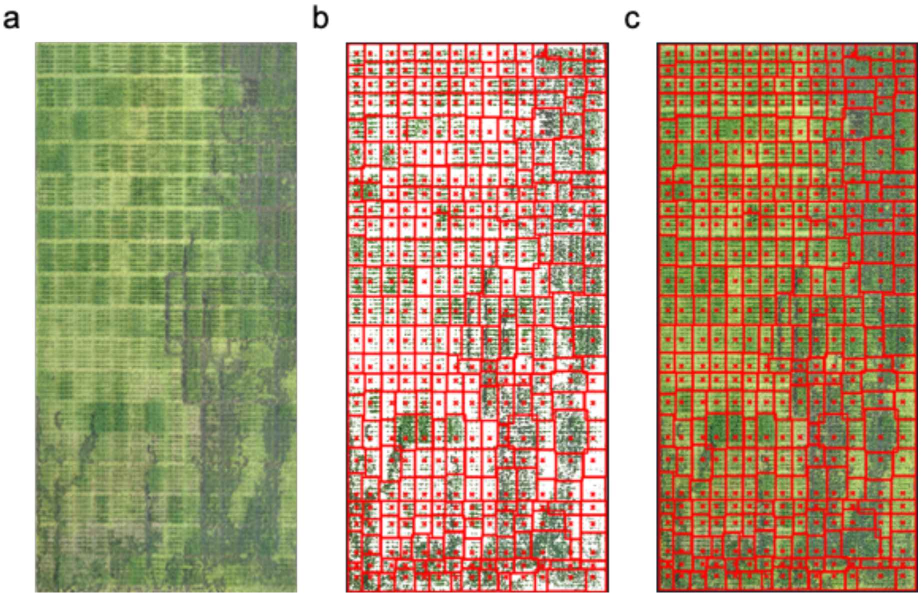

Figure S5.4 Plot segmentation results using GRID (53 DAS)

(**a**) An orthomosaic image acquire at 53 DAS. (**b**) Plot segmentation performed using GRID. (**c**) Segmentation results overlapped with the orthomosaic.

5.4 Plot segmentation using FIELDimageR and R/UAStools::plotshpcreate

FIELDimageR (Matias *et al.*, 2020) requires users to manually rotate the input image and then divide vegetation index using soil signals, followed by the manual input of the number of rows and columns of the field experiment layout. We applied the FIELDimageR software to process rice paddy field and encountered a variety of problems, including missing plot boundary information through binarizing the experimental field (**Fig. S5.5a**), and the segmented plots mismatched with the actual field layout (particular in the middle of the field; **Fig. S5.5b**) as most of the plots were not distanced evenly which is common in trials even with RTK-assisted seed drilling. Also, FIELDimageR took over 15 minutes to realign the input orthomosaic image; and, AirMeasurer only spent 20 seconds to accomplish the same task.

Finally, we tested R/UAStools::plotshpcreate (Anderson & Murray, 2020) to create multi-polygon shapefiles to extract plots. The software package requires users to enter experimental design (e.g. planting layout, field orientation based on the deviation of the north-south direction, and plot size). Also, it needs users to pre-install several third-party software such as QGIS to extract A-B Line coordinates (used for measuring the angle of the field), which is different from the GUI-based AirMeasurer, as well as the open-source and modular systems design when we were developing the AirMeasurer platform. Too many input parameters and third-party software packages could result in a sharper learning curve for non-expert researchers. We did not test AirSurf (Bauer *et al.*, 2019) as it was not designed to segment plant plots.

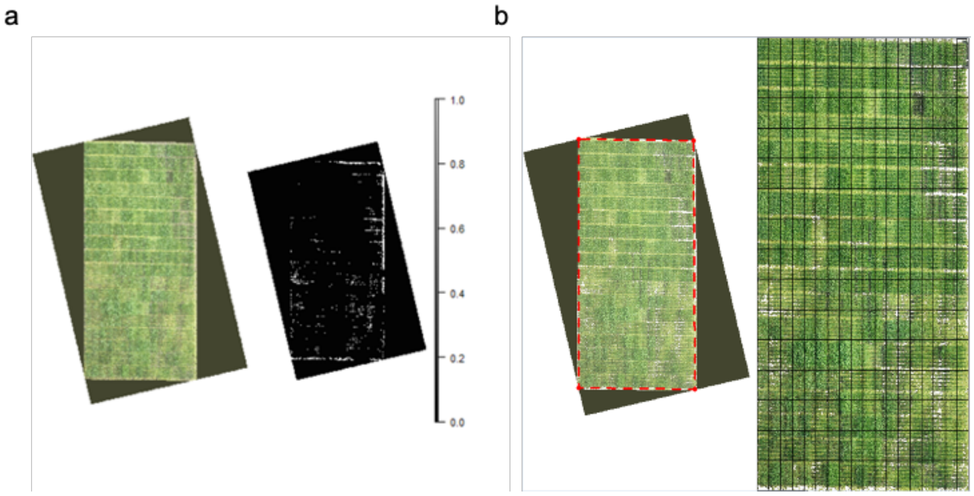

Figure S5.5 Plot segmentation results using FIELDimageR (53 DAS)

(**a**) An orthomosaic image acquire at 53 DAS and processed by FIELDimageR. (**b**) Plot segmentation results generated by FIELDimageR after manually inputting parameters such as the field orientation, and the number of rows and columns of the field.

Supporting Information Notes S6 – Source code of the plot segmentation algorithm

6.1 Step 1 – Input a CHM image

```
# Apply 2D perspective transform to the CHM
chm_path = r"C:\Users\Think\Desktop\test\3D_point_cloud\chm20190807.tif"
       RTK_information_path = r"C:\Users\Think\Desktop\test\RTK_information\s_mtps.shp"
dst_PT,columns_val,rows_val=transform(chm_path, RTK_information_path)


# The transform function to CHM image for standard analysis
def transform(chm_path, RTK_information_path):
    sf = shapefile.Reader(RTK_information_path,'rb')
    shapes = sf.shapes()
    list_p = []
    num=0
    for i in shapes:
        list_p.append(shapes[num].points[0])
        num+=1
    dataset = rasterio.open(chm_path)
    # transfer geo-coordinates into 2D coordinates in the CHM image
    upper_left_x, upper_left_y = (list_p[0][0],list_p[0][1])
    upper_left_row, upper_left_col = dataset.index(upper_left_x, upper_left_y)
    lower_left_x, lower_left_y = (list_p[1][0],list_p[1][1])
    lower_left_row, lower_left_col = dataset.index(lower_left_x, lower_left_y)
    upper_right_x, upper_right_y = (list_p[3][0],list_p[3][1])
    upper_right_row, upper_right_col = dataset.index(upper_right_x, upper_right_y)
    lower_right_x, lower_right_y = (list_p[2][0],list_p[2][1])
     lower_right_row, lower_right_col = dataset.index(lower_right_x, lower_right_y)
     # Calculate distance
    columns_1 = np.int64(math.sqrt((upper_left_row-lower_left_row)**2
                              + (upper_left_col-lower_left_col)**2))
    columns_2 = np.int64(math.sqrt((upper_right_row-lower_right_row)**2
                              + (upper_right_col-lower_right_col)**2))
    columns_val = np.max((columns_1,columns_2))
    rows_1 = np.int64(math.sqrt((upper_left_row-upper_right_row)**2
                           + (upper_left_col-upper_right_col)**2))
    rows_2 = np.int64(math.sqrt((lower_left_row-lower_right_row)**2
                           + (lower_left_col-lower_right_col)**2))
    rows_val = np.max((rows_1,rows_2))
    # Read image for 2D perspective transform
    img_tif = io.imread(chm_path)
     pts1 = np.float32([[upper_left_col,upper_left_row],[upper_right_col,
     upper_right_row],[lower_left_col,lower_left_row],
    [lower_right_col,lower_right_row]])
    pts2 = np.float32([[0,0],[rows_val,0],[0,columns_val],[rows_val,columns_val]])
    M_PT = cv2.getPerspectiveTransform(pts1,pts2)
    dst_PT = cv2.warpPerspective(img_tif,M_PT,(rows_val,columns_val))
     return dst_PT, columns_val, rows_val
# End of the transform function
```

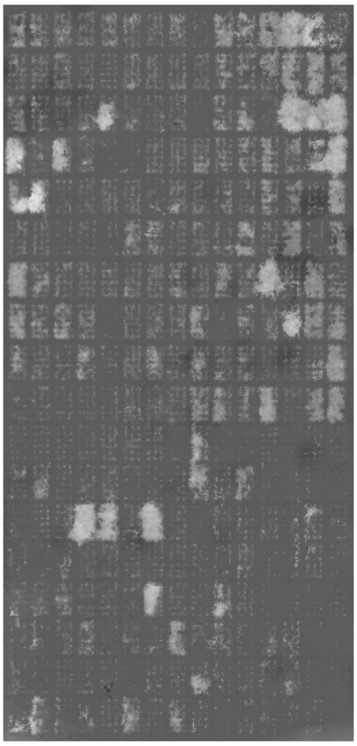

Figure S6.1 A CHM image after 2D perspective transformation

6.2 Step 2 – Binarization of the CHM with different kernels

```
# Use the binarization function to process the CHM
ero_all = binarization(dst_PT)

# The binarization function to process the input CHM image
def binarization(dst_PT):
    ero_all = []
    kernel_size = [5,8,26]
    for size in range(len(kernel_size)):
        thresh = filters.threshold_isodata(dst_PT)
        dst_th =(dst_PT <= thresh)*1.0
        kernel = cv2.getStructuringElement(cv2.MORPH_ELLIPSE,(kernel_size[size],
               kernel_size[size]))
        ero = cv2.erode(dst_th,kernel,iterations = 1)
        ero_all.append(ero)
    return ero_all
# End of the binarization function
```

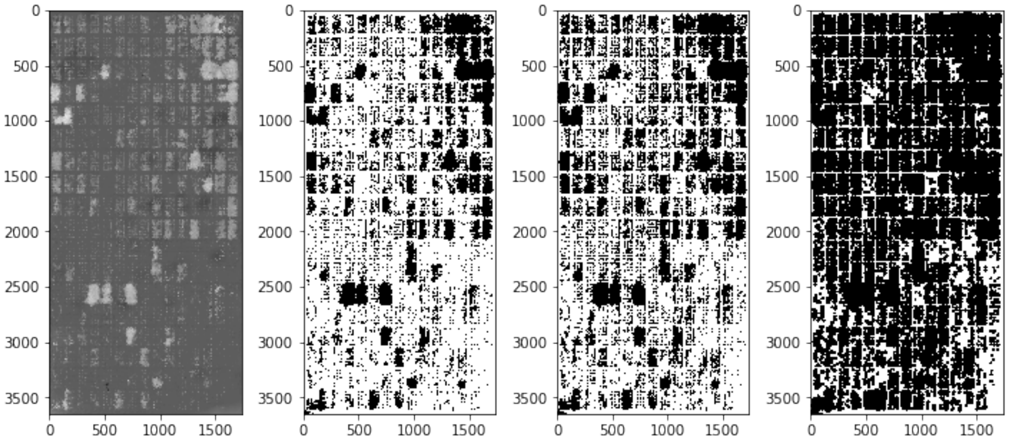

Figure S6.2 Binarization results (field level masks) with different processing kernels

6.3 Step 3 – Detect horizonal lines from the field level mask

```
# Use the line_horizontal function to process all the field-level masks
hough_h_se = line_horizontal(dst_PT, ero_all)

# The line_horizontal function to process the input field-level masks
def line_horizontal(dst_PT, ero_all):
    # Filter horizontal lines function
    def line_merge_h(lines,dif):
        lines.sort(); cons = []
        for i in range(len(lines)-1):
            if lines[i+1] - lines[i] < dif:
                avg = lines[i]; cons.append(avg)
            elif lines[i] - lines[i-1] >= dif and i != 0:
                cons.append(lines[i])
            elif i == 0:
                cons.append(lines[i])
        if lines[-1] - lines[-2] >= dif:
            cons.append(lines[-1])
        return cons
    # Use Hough transform to detect horizontal lines
    hough_h_m_all = []; hor_cons_all = []; hor_cons_num = []
    height,width = dst_PT.shape[:2]
    for i in range(len(ero_all)):
        hough_bw_h = np.zeros((height,width))
        tested_angles = np.linspace(np.pi / 2, np.pi / 2, 10)
        h, theta, d = hough_line(ero_all[i], theta=tested_angles)
        _, angle, dist=hough_line_peaks(h, theta, d)
        d_sort=np.sort(dist); d_dif = np.diff(d_sort)
        d_dif_max_h = d_dif.max()*0.65
        origin = np.array((0, dst_PT.shape[1]))
        for _, angle, dist in zip(*hough_line_peaks(h, theta, d)):
            y0, y1 = (dist - origin * np.cos(angle)) / np.sin(angle)
            cv2.line(hough_bw_h,(0, int(y0)),(width, int(y1)),255,6)
        # Produce lines based on the line drawing
        hough_bw_h = np.bitwise_not(hough_bw_h.astype("uint8"))
        hough_h_inv=util.invert(hough_bw_h)
        # Begin to filter horizontal lines
```

```
        lines_h = cv2.HoughLines(hough_h_inv.astype("uint8"),1,np.pi/90,100)
        hor = []
        for line in lines_h:
            if line[0][1] > 1.55 and line[0][1] < 1.58:
                hor.append(line[0,0])
        hor.sort(); hor_cons = []
        while len(hor_cons) != len(hor):
            hor_cons = hor; hor = line_merge_h(hor,d_dif_max_h)
        hor_cons_num.append(len(hor_cons))
        hor_cons_all.append(hor_cons)
        hough_h_m = np.zeros((height,width))
        for i in hor_cons:
            cv2.line(hough_h_m,(0, int(i)),(width, int(i)),255,1)
        hough_h_m = np.bitwise_not(hough_h_m.astype("uint8"))
        hough_h_m = util.invert(hough_h_m)
         # Append analysis result
        hough_h_m_all.append(hough_h_m)
        # End of internal function
    # Return detected lines
    se = hor_cons_num.index(max(hor_cons_num))
    hor_cons_se = hor_cons_all[se]; hough_h_se = hough_h_m_all[se]
    return hough_h_se
# End of the line_horizontal function
```

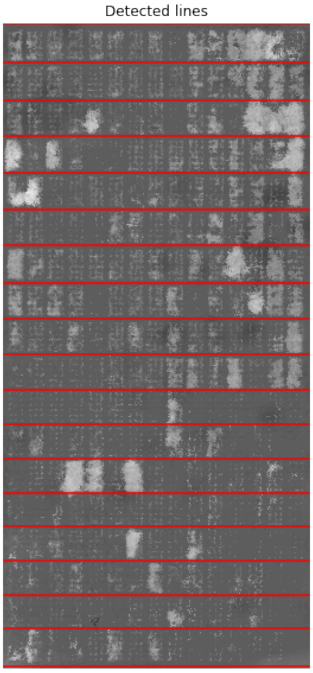

Figure S6.3 Result of the horizontal line detection using Hough transformation

6.4 Step 4 – Detect vertical lines from the field level mask

```
# Use the line_vertical function to process all the field-level masks
def line_vertical(dst_PT, ero_all):
    # The line_vertical function to process the input field-level masks
    def line_merge_v(lines,dif):
        lines.sort()
        cons = []
        for i in range(len(lines)-1):
            if lines[i+1] - lines[i] < dif:
```

```
                avg = (lines[i+1]+lines[i])/2
                cons.append(avg)
            elif lines[i] - lines[i-1] >= dif and i != 0:
                cons.append(lines[i])
            elif i == 0:
                cons.append(lines[i])
        if lines[-1] - lines[-2] >=dif:
            cons.append(lines[-1])
        return cons

    # Use Hough transform to detect vertical lines in the mask
    hough_v_m_all = []; ver_cons_all = []; ver_cons_num = []
    height,width = dst_PT.shape[:2]
    for i in range(len(ero_all)):
        hough_bw_v = np.zeros((height,width))
        out = dst_PT.copy()
        lines_v_or = cv2.HoughLines(ero_all[i].astype("uint8"),0.1,
                      np.pi/1,int(height*0.52))
        dist_v = []
        for line in lines_v_or:
            if line[0][1] > 0.01 and line[0][1] < 1.55:
                continue
            for rho,theta in line:
                a = np.cos(theta); b = np.sin(theta)
                x0 = a*rho; y0 = b*rho
                x1 = int(x0 + height*(-b)); y1 = int(y0 + height*(a))
                x2 = int(x0 - height*(-b)); y2 = int(y0 - height*(a))
                dist_v.append(rho)
                cv2.line(out,(x1,y1),(x2,y2),(0,0,255),1)
                cv2.line(hough_bw_v,(x1,y1),(x2,y2),255,1)
        d_sort_v=np.sort(dist_v)
        d_dif_v = np.diff(d_sort_v)
        d_dif_max_v = d_dif_v.max()*0.45
        hough_bw_v = np.bitwise_not(hough_bw_v.astype("uint8"))
        hough_v_inv = util.invert(hough_bw_v)

        # Filter vertical lines that are clustered
        lines_v = cv2.HoughLines(hough_v_inv.astype("uint8"),1,np.pi/90,100)
        ver = []
        for line in lines_v:
            if line[0][1] < 0.01:
                ver.append(line[0,0])
        ver.sort(); ver_cons = []
        while len(ver_cons) != len(ver):
            ver_cons = ver
            ver = line_merge_v(ver,d_dif_max_v)
        ver_cons_num.append(len(ver_cons))
        ver_cons_all.append(ver_cons)
        hough_v_m = np.zeros((height,width))
        for i in ver_cons:
            cv2.line(hough_v_m,(int(i),0),(int(i), height),255,1)
        # Line merge
        hough_v_m = np.bitwise_not(hough_v_m.astype("uint8"))
        hough_v_m = util.invert(hough_v_m)
        hough_v_m_all.append(hough_v_m)

    se = ver_cons_num.index(np.median(ver_cons_num))
    ver_cons_se = ver_cons_all[se]
    hough_v_se = hough_v_m_all[se]

    # Visualise the final result
    fig, ax = plt.subplots(figsize=(10, 10))
    ax.imshow(dst_PT, plt.cm.gray)
    origin = np.array((0, dst_PT.shape[1]))
    origin_0 = np.array((0, dst_PT.shape[0]))
    for i in ver_cons_se:
        ax.plot((i,i),origin_0,'-r',6)
    ax.set_axis_off()
    ax.set_title('Detected lines')
    return hough_v_se

# End of the line_vertical function
```

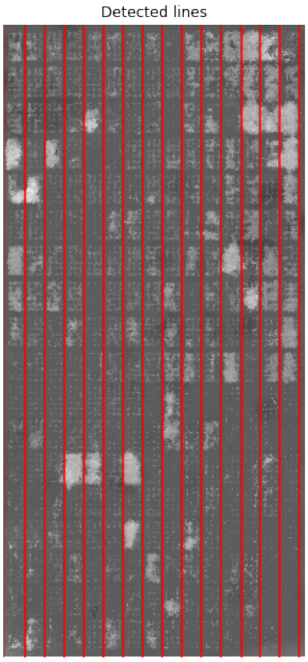

Figure S6.4 Result of the vertical line detection using Hough transformation

6.5 Step 4.1 (optional) – Use base lines to assist with horizontal line detection

```
# Use the base_line_horizontal function to provide horizontal base lines if Step 3 could not
detect all the horizontal lines based on the field mask
def base_line_horizontal(dst_PT, hor_cons_se, column_number,columns_val_base):
    # Add the horizontal base lines based on the row number of the field
    blank_img = np.zeros((dst_PT.shape[0],dst_PT.shape[1]))
    h_value = (columns_val_base)/column_number
    h_line_base = blank_img.copy()
    h_line_rgb = np.zeros((dst_PT.shape[0],dst_PT.shape[1],3),np.uint8)
    inds_y_h = dst_PT.shape[1]-1
    inds_x_h = np.arange(30, len(h_line_base)-40, int(h_value))
    h_line_base[inds_x_h, :inds_y_h] = 1
    for i in inds_x_h:
        cv2.line(h_line_rgb,(0, i),(inds_y_h, i),(255,255,0),6)
    lines_h_base = cv2.HoughLines(h_line_base.astype("uint8"),1,np.pi/90,100)
    hor_base = []
    for line in lines_h_base:
        if line[0][1] > 1.55 and line[0][1] < 1.58:
            hor_base.append(line[0,0])

    #Combine the horizontal base lines with the lines detected by the Hough Transform
    fig, axes = plt.subplots(1, 2, figsize=(10, 10))
    ax = axes.ravel()
    ax[0].imshow(h_line_rgb)
    ax[0].set_axis_off()
    ax[1].imshow(dst_PT,plt.cm.gray)
    origin = np.array((0, dst_PT.shape[1]))
     origin_0 = np.array((0, dst_PT.shape[0]))
     # Assemble horizontal lines
    for i in hor_base:
```

```
        ax[1].plot(origin,(i,i),'-y',6)
    for j in hor_cons_se:
        ax[1].plot(origin,(j,j),'-r',6)
    ax[1].set_xlim(origin)
    ax[1].set_ylim((dst_PT.shape[0], 0))
    ax[1].set_axis_off()
    plt.tight_layout()
    plt.show()
    return h_line_base, hor_base
# End of the base_line_horizontal function
```

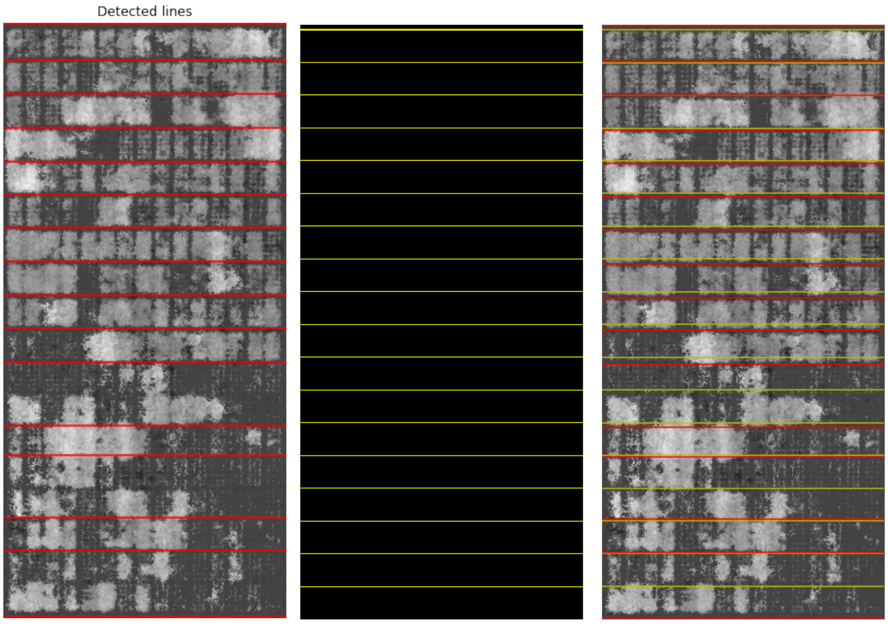

Figure S6.5 Result of the hortizonal line detection (red) together with horizontal base lines (yellow)

6.6 Step 4.2 (optional) – Use base lines to assist with vertical line detection

```
# Use the base_line_vertical function to provide vertical base lines if Step 4 could not detect
all the vertical lines based on the plot boundaries

def base_line_vertical(dst_PT, ver_cons_se, row_number, rows_val_base):

    # Add the vertical base lines according the input column number
    blank_img = np.zeros((dst_PT.shape[0],dst_PT.shape[1]))
    v_value = rows_val_base/row_number
    v_line_base = blank_img.copy()
    v_line_rgb = np.zeros((dst_PT.shape[0],dst_PT.shape[1],3),np.uint8)
    inds_x_v = dst_PT.shape[0]-1
    inds_y_v = np.arange(0, dst_PT.shape[1], int(v_value))
     v_line_base[:inds_x_v, inds_y_v] = 1

    # draw lines
    for j in inds_y_v:
        cv2.line(v_line_rgb,(j,0),(j, inds_x_v),(255,255,0),6)
    # Perform Hough transform
    lines_v_base = cv2.HoughLines(v_line_base.astype("uint8"),1,np.pi/90,100)
    ver_base = []
    for line in lines_v_base:
```

```
        if line[0][1] < 0.01:
            ver_base.append(line[0,0])

    #Combine the vertical base lines with the lines detected by the Hough Transform
    fig, axes = plt.subplots(1, 2, figsize=(10, 10))
    ax = axes.ravel()
    ax[0].imshow(v_line_rgb)
    ax[0].set_axis_off()
    ax[1].imshow(dst_PT,plt.cm.gray)
    origin = np.array((0, dst_PT.shape[1]))
    origin_0 = np.array((0, dst_PT.shape[0]))
    for i in ver_base:
        ax[1].plot((i,i),origin_0,'-y',6)
    for j in ver_cons_se:
        ax[1].plot((j,j),origin_0,'-r',6)
    ax[1].set_xlim(origin)
    ax[1].set_ylim((dst_PT.shape[0], 0))
    ax[1].set_axis_off()
    plt.tight_layout()
    plt.show()
    return v_line_base, ver_base
# End of the base_line_vertical function
```

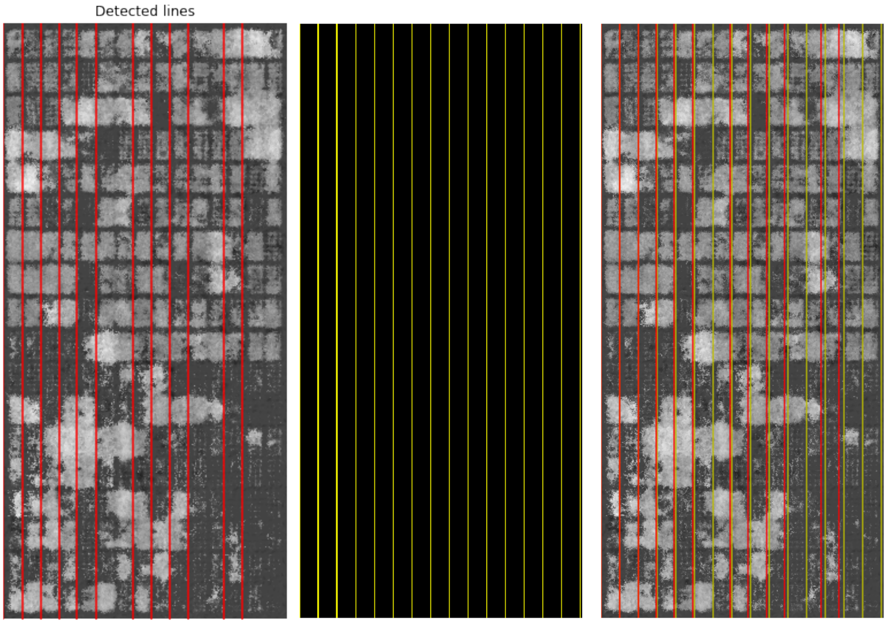

Figure S6.6 Result of the vertical line detection (red) together with vertical base lines (yellow)

6.7 Step 5 – Assemble all the horizontal and vertical lines to present plots in the field

```
# Use the base_line_horizontal function to provide horizontal base lines if Step 3 could not
detect all the vertical lines based on the plot boundaries

# The line_hough_merge function to merge closed lines
def line_hough_merge(hough_h_se, hough_v_se, dst_PT):
    hough_merge_line = np.logical_or(hough_h_se, hough_v_se)
    lines_merge = cv2.HoughLines(hough_merge_line.astype("uint8"),1,np.pi/90,100)
    hor = [] # The collection of horizonal lines
     ver = [] # The collection of verticle lines
```

```
    # Iteration to go through all the lines that can be merged
    for line in lines_merge:
        if line[0][1] < 0.01:
            ver.append(line[0,0])
        elif line[0][1] > 1.55 and line[0][1] < 1.58:
            hor.append(line[0,0])
     # Select lines and draw them on the image
     fig, ax = plt.subplots(figsize=(10, 10))
     ax.imshow(dst_PT, plt.cm.gray)
    origin = np.array((0, dst_PT.shape[1]))
    origin_0 = np.array((0, dst_PT.shape[0]))
    for i in ver:
        ax.plot((i,i),origin_0,'-r',6)
    for j in hor:
        ax.plot(origin,(j,j),'-r',6)
    ax.set_xlim(origin)
    ax.set_ylim((dst_PT.shape[0], 0))
    ax.set_axis_off()
    ax.set_title('Detected lines')
     return hough_merge_line
# End of the line_hough_merge function
```

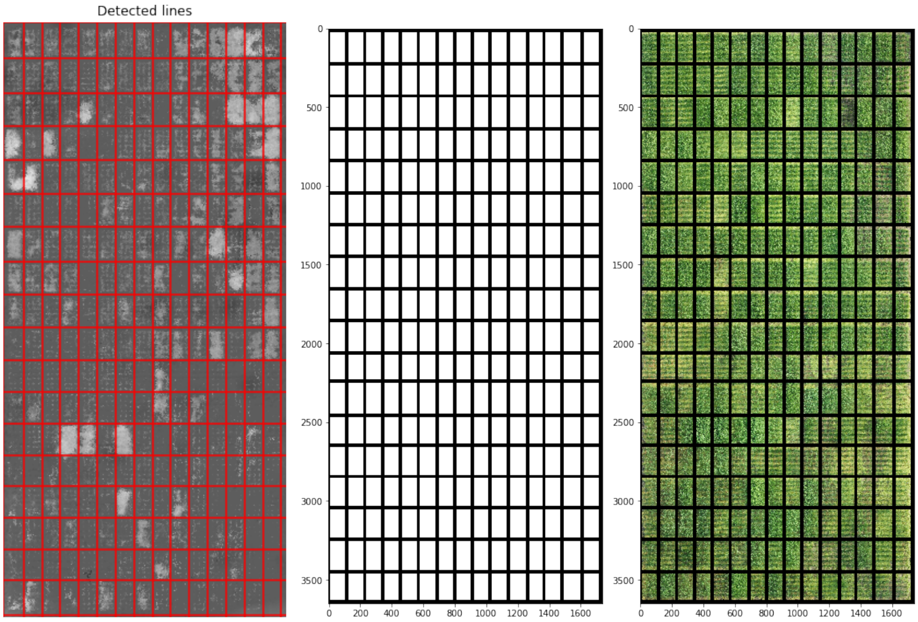

Figure S6.7 Assemble all the lines (red) to generate a field-level mask to segment plots in the field

Supporting Information Notes S7 – The reasoning behind choosing H_{90th} for height measurement

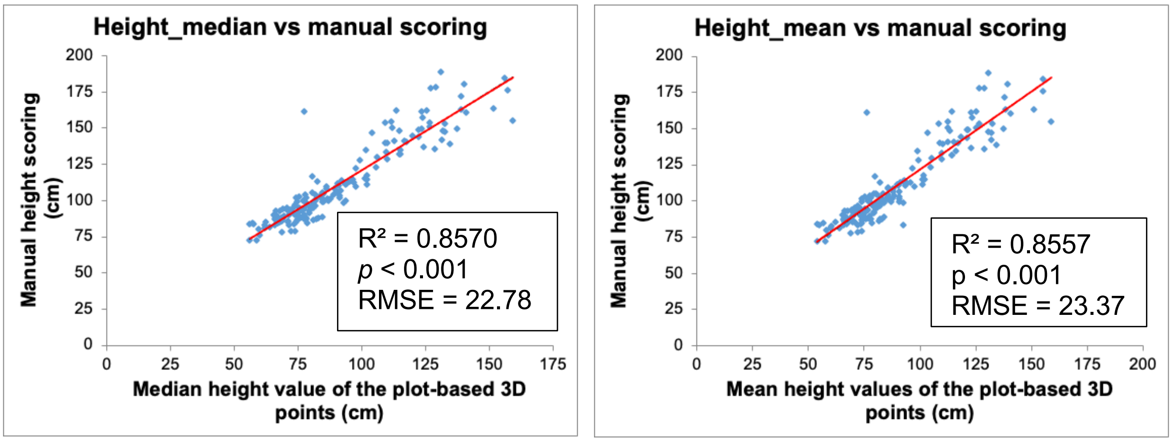

Figure S7.1 Correlation analysis between manual scored maximum plant height and the mean or median height values sampled from plot-based 3D points in the 2019 season for rice landraces

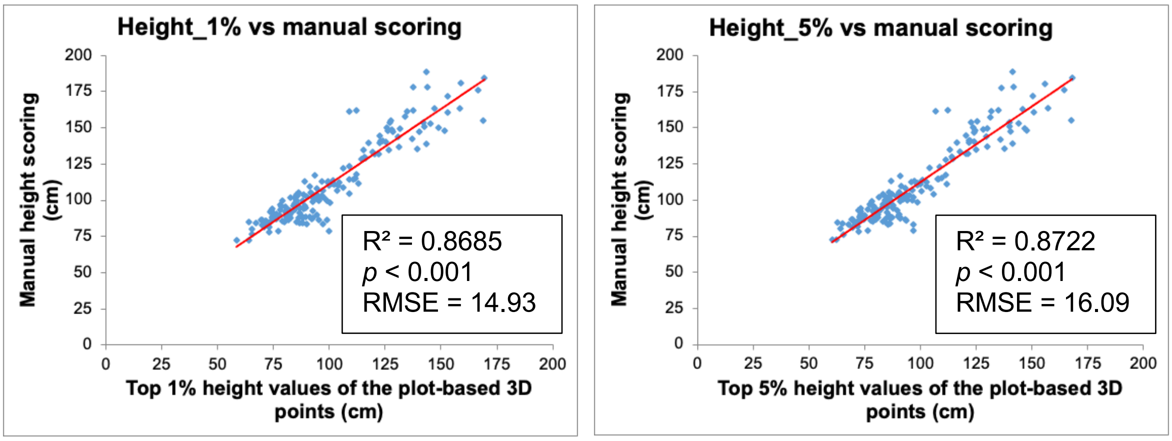

Figure S7.2 Correlation analysis between manual scored maximum plant height and the top 1% or 5% height values sampled from plot-based 3D points in the 2019 season for rice landraces

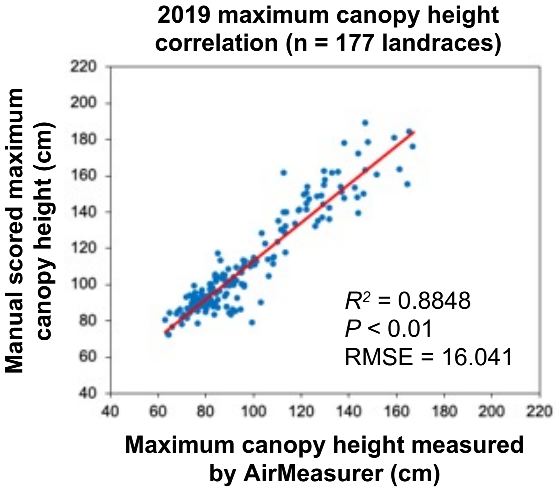

Figure S7.3 Correlation between manual scored maximum plant height and the top 10% height values (H_{90th}) sampled from plot-based 3D points in the 2019 season for rice landraces.

Supporting Information Notes S8 – Source code for computing canopy coverage and ExG indices

```
# Combine ExG and canopy coverage for plot-level canopy analysis
def plot_exg_canopycov(image_dir, chm_dir, RTK_information_path, mask_path, scale_factor):
    np.seterr(divide='ignore',invalid='ignore')
    mask_line = io.imread(mask_path)
    selem = disk(7)
    Grid_Image = dilation(mask_line, selem)
    Grid_Segment = np.logical_not(Grid_Image)
    Grid_Segment_Refine = erosion(Grid_Segment, disk(3))
    list_bbox_area = []
    Labelled_Plot_Img_o, num_features_o = ndi.measurements.label(Grid_Segment_Refine)
    for region in regionprops(Labelled_Plot_Img_o):
        list_bbox_area.append(region.area)
    re_area = np.max(list_bbox_area)*0.4
    Grid_Segment_Refine = remove_small_objects(Grid_Segment_Refine, re_area)
    Labelled_Plot_Img, num_features = ndi.measurements.label(Grid_Segment_Refine)
    height,width = Labelled_Plot_Img.shape[:2]
    test_dir=os.path.abspath(os.path.dirname(chm_dir))
    try:
        os.mkdir(test_dir+'\\'+'result')
        result_dir = test_dir+'\\'+'result'
    except FileExistsError:
        result_dir = test_dir+'/'+'result'
    try:
        os.mkdir(result_dir+'\\'+'exg')
        exg_dir=result_dir+'\\'+'exg'
        os.mkdir(result_dir+'\\'+'canopy')
        canopy_dir=result_dir+'\\'+'canopy'
    except FileExistsError:
        exg_dir=result_dir+'\\'+'exg'
        canopy_dir=result_dir+'\\'+'canopy'
    sf = shp.Reader(RTK_information_path,'rb')
    shapes = sf.shapes()
    list_p = []
    num=0
    for i in shapes:
        list_p.append(shapes[num].points[0])
        num+=1
    geo_coords = coor_sort_new(list_p)
    chm_path = os.listdir(chm_dir)
    img_path = os.listdir(image_dir)

    for name in tqdm(range(len(chm_path))):
        dataset = rasterio.open(chm_dir+'\\'+chm_path[name])
        upper_left_x, upper_left_y = (geo_coords[0][0],geo_coords[0][1])
        upper_left_row, upper_left_col = dataset.index(upper_left_x, upper_left_y)
        lower_left_x, lower_left_y = (geo_coords[1][0],geo_coords[1][1])
        lower_left_row, lower_left_col = dataset.index(lower_left_x, lower_left_y)
        upper_right_x, upper_right_y = (geo_coords[2][0],geo_coords[2][1])
        upper_right_row, upper_right_col = dataset.index(
```

```
                                    upper_right_x, upper_right_y)
lower_right_x, lower_right_y = (geo_coords[3][0],geo_coords[3][1])
lower_right_row, lower_right_col = dataset.index(
                                    lower_right_x, lower_right_y)
columns_1 = np.int64(math.sqrt((upper_left_row-lower_left_row)**2
                              + (upper_left_col-lower_left_col)**2))
columns_2 = np.int64(math.sqrt((upper_right_row-lower_right_row)**2
                              + (upper_right_col-lower_right_col)**2))
columns_val = np.max((columns_1,columns_2))
rows_1 = np.int64(math.sqrt((upper_left_row-upper_right_row)**2
                           + (upper_left_col-upper_right_col)**2))
rows_2 = np.int64(math.sqrt((lower_left_row-lower_right_row)**2
                           + (lower_left_col-lower_right_col)**2))
rows_val = np.max((rows_1,rows_2))
dataset.close()
img_tif = io.imread(chm_dir+'\\'+chm_path[name])
pts1 = np.float32([[upper_left_col,upper_left_row],
                      [upper_right_col,upper_right_row],
                      [lower_left_col,lower_left_row],
                   [lower_right_col,lower_right_row]])
pts2 = np.float32([[0,0],[rows_val,0],
       [0,columns_val],[rows_val,columns_val]])
M_PT = cv2.getPerspectiveTransform(pts1,pts2)
dst_PT = cv2.warpPerspective(img_tif,M_PT,(rows_val,columns_val))
dst_th =(dst_PT >= np.percentile(dst_PT, 1))*1.0
kernel = cv2.getStructuringElement(cv2.MORPH_ELLIPSE, (5, 5))
erosion_chm=cv2.dilate(dst_th, kernel,iterations = 1)

# Load orthomosaic
dataset_rgb = rasterio.open(image_dir+'\\'+img_path[name])
height_tif, width_tif=dataset_rgb.shape[:2]
while height_tif>32767 or width_tif>32767:
    dataset_rgb.close()
    with fiona.open(RTK_information_path, "r") as shapefile:
        shapes = [feature["geometry"] for feature in shapefile]
    with rasterio.open(image_dir+'\\'+img_path[name]) as src:
        out_image, out_transform = rasterio.mask.mask(
                      src,shapes,filled=False)
        out_meta = src.meta
        transform, width_re, height_re
              = rasterio.warp.calculate_default_transform(
            src.crs, src.crs, out_image.shape[2]*0.5,
              out_image.shape[1]*0.5, *src.bounds)
    out_meta.update({"driver": "GTiff",
                 "height": out_image.shape[1]*0.5,
                 "width": out_image.shape[2]*0.5,
                 "transform": transform})
    with rasterio.open(image_dir+'\\'+img_path[name], "w",
       **out_meta) as dest:
        dest.write(out_image)
    dataset_rgb = rasterio.open(image_dir+'\\'+img_path[name])
    height_tif, width_tif=dataset_rgb.shape[:2]
```

```
upper_left_x, upper_left_y = (geo_coords[0][0],geo_coords[0][1])
upper_left_row, upper_left_col = dataset_rgb.index(
              upper_left_x, upper_left_y)
lower_left_x, lower_left_y = (geo_coords[1][0],geo_coords[1][1])
lower_left_row, lower_left_col = dataset_rgb.index(
              lower_left_x, lower_left_y)
upper_right_x, upper_right_y = (geo_coords[2][0],geo_coords[2][1])
upper_right_row, upper_right_col = dataset_rgb.index(
              upper_right_x, upper_right_y)
lower_right_x, lower_right_y = (geo_coords[3][0],geo_coords[3][1])
lower_right_row, lower_right_col = dataset_rgb.index(
              lower_right_x, lower_right_y)
img_rgb = io.imread(image_dir+'\\'+img_path[name])

pts1_rgb = np.float32([[upper_left_col,upper_left_row],
              [upper_right_col,upper_right_row],
              [lower_left_col,lower_left_row],
              [lower_right_col,lower_right_row]])
pts2_rgb = np.float32([[0,0],[width,0],[0,height],[width,height]])
M_PT_rgb = cv2.getPerspectiveTransform(pts1_rgb,pts2_rgb)
dst_PT_rgb = cv2.warpPerspective(img_rgb,M_PT_rgb,(width,height))

new_plot=dst_PT_rgb[:,:,:3] * (color.gray2rgb(
              img_as_uint(erosion_chm)*255)).astype(np.uint8)
plot_img_LAB = color.rgb2lab(new_plot)
a_2D_Color_ND = plot_img_LAB[:, :, 1]
b_2D_Color_ND = plot_img_LAB[:, :, 2]
LAB_Image_Ref = (b_2D_Color_ND - a_2D_Color_ND)
global_thresh_Ref_Points_Value = filters.threshold_otsu(LAB_Image_Ref)
if global_thresh_Ref_Points_Value > 0 and
       global_thresh_Ref_Points_Value >= LAB_Image_Ref.max() * 0.425:
        binary_global_LAB = LAB_Image_Ref > LAB_Image_Ref.max() * 0.75
          # close to 32% - tails of 1SD
elif global_thresh_Ref_Points_Value > 0 and global_thresh_Ref_Points_Value <
          LAB_Image_Ref.max() * 0.425:
        binary_global_LAB = LAB_Image_Ref > LAB_Image_Ref.max() * 0.35
# Close to 2 standard deviations, Was using 0.25 for 1SD
else: # global_thresh_Ref_Points_Value < 0, in very rare cases
        binary_global_LAB = LAB_Image_Ref > 0
# 1-pixel regions, expanded 1 pixel, which needs to be rescaled
Img_cleaned = remove_small_objects(binary_global_LAB, 1)
selem = disk(1)
Img_cleaned_Dilated = dilation(Img_cleaned, selem)
Img_cleaned_Ref = remove_small_holes(Img_cleaned_Dilated)
# In total 1 pixels have been expanded around the outline of every object
erode_binary_Img = erosion(Img_cleaned_Ref, disk(1))
erode_binary_Img = remove_small_objects(erode_binary_Img, 1)
chm_rgb_plot=dst_PT_rgb[:,:,:3] * (color.gray2rgb(erode_binary_Img))
list_cencoor = list()
Centroid_coordinates = list()
exg_mean = list()
canopy_coverage = list()
for region in regionprops(Labelled_Plot_Img):
```

```
    minr, minc, maxr, maxc = region.bbox
    col_val = ((maxc-minc)/2)*scale_factor  # Plot scale
    row_val = ((maxr-minr)/2)*scale_factor
    Centroid_coordinates.append(region.centroid)
    list_cencoor.append(region.centroid[1])
    roi=chm_rgb_plot[int(region.centroid[0]-row_val):
       int(region.centroid[0]+row_val),
      int(region.centroid[1]-col_val):
       int(region.centroid[1]+col_val)]
    canopy_num_all = len(erode_binary_Img[
       int(region.centroid[0]-row_val):
       int(region.centroid[0]+row_val),
     int(region.centroid[1]-col_val):
       int(region.centroid[1]+col_val)].flatten())
    canopy_num = len(erode_binary_Img[
       int(region.centroid[0]-row_val):int(region.centroid[0]+
       row_val), int(region.centroid[1]-col_val):
       int(region.centroid[1]+col_val)]
     [erode_binary_Img[int(region.centroid[0]-row_val):
       int(region.centroid[0]+row_val),
     int(region.centroid[1]-col_val):
       int(region.centroid[1]+col_val)]==True])
    canopy_cov = canopy_num/canopy_num_all
    canopy_coverage.append(canopy_cov)
    # exg
    img_rgb = roi.astype(np.float64)
    r, g, b = cv2.split(img_rgb[:,:,0:3])
    r=r[erode_binary_Img[int(region.centroid[0]-row_val):
       int(region.centroid[0]+row_val),
     int(region.centroid[1]-col_val):
       int(region.centroid[1]+col_val)]==True]
    g=g[erode_binary_Img[int(region.centroid[0]-row_val):
       int(region.centroid[0]+row_val),
      int(region.centroid[1]-col_val):
       int(region.centroid[1]+col_val)]==True]
    b=b[erode_binary_Img[int(region.centroid[0]-row_val):
       int(region.centroid[0]+row_val),
       int(region.centroid[1]-col_val):
       int(region.centroid[1]+col_val)]==True]
    sum = r + g + b
    r = np.divide(r, sum)
    g = np.divide(g, sum)
    b = np.divide(b, sum)
    ex_g = 2.0 * g - r - b
    exg_mean.append(np.mean(ex_g))

diff_rows = np.diff(np.array(Centroid_coordinates)[:,0])
thresh = filters.threshold_isodata(diff_rows)
rows_fil=list(filter(lambda x:x>thresh, diff_rows))
rows_fil = len(rows_fil)+1
columns = int(len(Centroid_coordinates)/rows_fil)
row_index=[]
column_index=[]
```

```
for i in range(int(rows_fil)):
    for j in range(int(columns)):
        row_index.append(i+1)
        column_index.append(j+1)
Centroid_coordinates_y_array = np.array(list_cencoor)
canopy_array = np.array(canopy_coverage)
exg_array = np.array(exg_mean)
canopy_new = []
exg_new = []
for n in range(0, rows_fil):
    sub_array_y = Centroid_coordinates_y_array[n*columns:columns+n*columns]
    sub_array_canopy = canopy_array[n*columns:columns+n*columns]
    sub_array_exg = exg_array[n*columns:columns+n*columns]
    rank = np.argsort(sub_array_y)
    sub_array_canopy = sub_array_canopy[rank]
    sub_array_exg = sub_array_exg[rank]

    for i in range(0,sub_array_canopy.shape[0]):
        sub_element_canopy = sub_array_canopy[i]
        sub_element_exg = sub_array_exg[i]
        canopy_new.append(sub_element_canopy)
        exg_new.append(sub_element_exg)

dt_exg = pd.DataFrame({'rows':
       row_index, 'columns':column_index,'exg':exg_new})
       dt_exg.to_csv(exg_dir+'\\'+chm_path[name][3:-4]+'_exg'+'.csv',encoding="gbk")
display(dt_exg)

dt_canopy = pd.DataFrame({'rows':
       row_index, 'columns':column_index,'canopy_coverage':canopy_new})
       dt_canopy.to_csv(canopy_dir+'\\'+chm_path[name][3:-
       4]+'_canopy_coverage'+'.csv',encoding="gbk")
display(dt_canopy)
```

Supporting Information Notes S9 – Vegetative indices and texture-based traits

9.1 Vegetative indices integrated in AirMeasurer

UAV-mounted RGB, multi- and hyper-spectral image sensors are often used to calculate vegetative indices based on spectral reflectance from plant canopy (Xue & Su, 2017). Although the price of spectral sensors has been decreasing in recent years, they often remain out of reach for many researchers. Therefore, we used RGB signals and their derived colour spaces to compute growth-related vegetation indices. We computed: (1) ExG and excessive red (ExR, using the equation "1.4 * R – B") to enhance weights of the green and red channels (Meyer & Neto, 2008); (2) *normalised vegetative index* (NVI), using the equation "ExG – ExR" to monitor vegetation changes; (3) the *visible atmospherically resistant index* (VARI), using the equation "(G – R)/(G + R – B)", which has been associated with plant health due to loss of green foliage caused by abiotic or biotic stresses (Gitelson *et al.*, 2002); (4) the *normalized difference yellowness index* (NDYI), using the equation "(G – B)/(G + B)", has been related to canopy longevity and senescence, correlating with relative yield potential (Sulik & Long, 2016). When calculating vegetative indices, we chose to remove outliers by setting boundaries to 1.5 * the interquartile range (IQR) in every colour channel and normalised indices (0-1) for cross-referencing.

9.2 Texture-based indices

Besides vegetative indices, we also integrated textural analysis into AirMeasurer to describe canopy expansion and structural changes. Textural analysis was based on overhead plant canopy images, including: (1) canopy uniformity, using angular second moment, ASM (Zhang & Tang, 2018), which measures the homogeneity of the distribution of ExG elements (i.e. enhanced leaf signals) and the size of their texture (Sun *et al.*, 2020); if the values of green leaf objects are distributed centrally, the ASM values tend to be larger, indicating the distribution of canopy is more uniform; (2) canopy dissimilarity, using the correlation in the grayscale co-occurrence matrices, GLCMs (Liu *et al.*, 2012) to estimate the similarity of the grey values in row or column directions; when directional textures appeared (e.g. lodging), the value of the matrix tends to be larger. The values of the above traits were normalised before being used in cross referencing between trials.

Supporting Information Notes S10 – A step-by-step user guide of the AirMeasurer GUI

The graphic user interface (GUI) for AirMeasurer was developed using the Python native GUI package, Tkinter. The GUI software (in EXE format) can be executed on Windows operating system (Windows 10 tested). Users can run the software by double clicking the .exe executable, which can be downloaded from our GitHub repository (https://github.com/The-Zhou-Lab/UAV/releases/tag/V2.0). On the initial GUI window (**Fig. S10.1**), users can select 2D orthomosaics (in TIFF) and associated 3D point clouds (in LAS), as well as 3D- or geo-coordinates (i.e. RTK information, in SHP format) to initiate the initial analysis. The introduction of the input section can be displayed by clicking the information icons (coloured blue) in each input section.

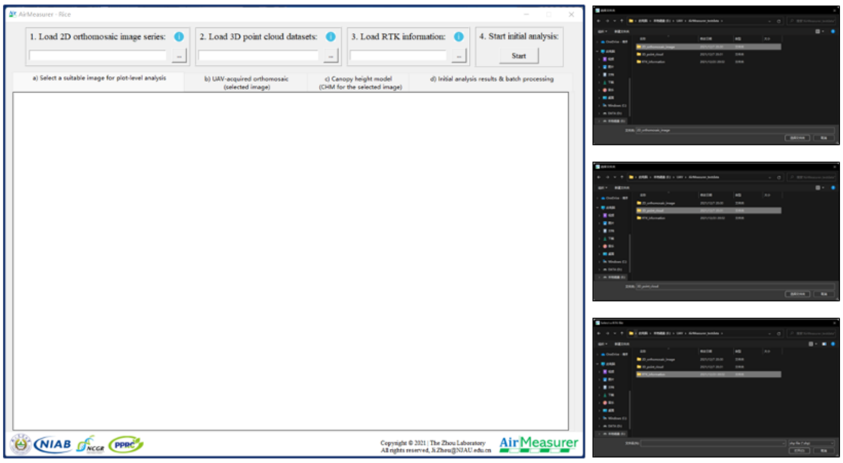

Figure S10.1 The initial GUI of AirMeasurer

10.1 Select an input image to process

After selecting the input datasets, the user can initiate the analysis by clicking the 'Start' button. Then, AirMeasurer will automatically process and display all the input 2D orthomosaic images in the "tab a" (**Fig. S10.2**), showing the central parts of all the 2D orthomosaics (over 700 MB per image).

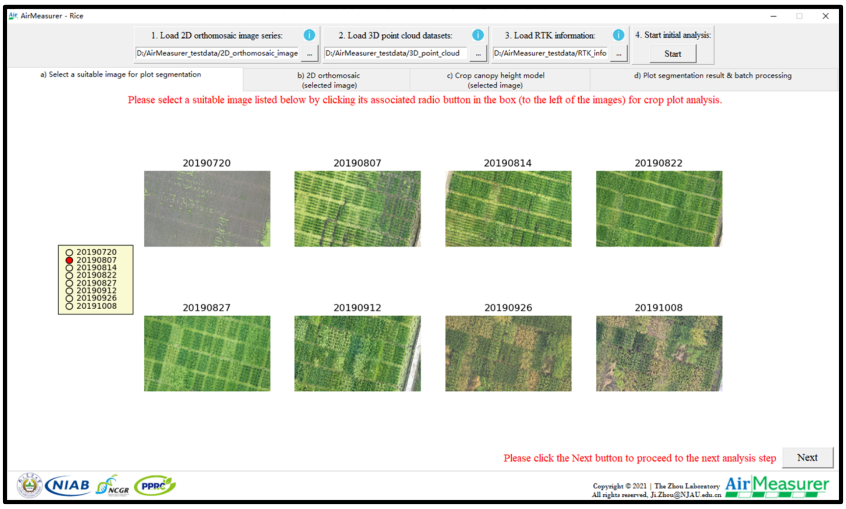

Figure S10.2 Select an orthomosaic image to begin the initial analysis

By only displaying the central parts of the 2D orthomosaics, AirMeasurer can considerably reduce the display time (less than 30 seconds to display eight 2D orthomosaic images with a total size of 5.7 GB). Based on the eight input images, users can select an image using its associated radio button to begin the initial analysis. Users are required to click the 'Next' button to proceed to the next step.

10.2 Initial plot segmentation using the GUI

The 'tab b' displays the entire field-level 2D orthomosaic image selected in the 'tab a' together with a compass, straight towards the north of the experimental field, followed by the 3D- or geo-referenced image within the region of interest (ROI) defined by the input SHP file (**Fig. S10.3**).

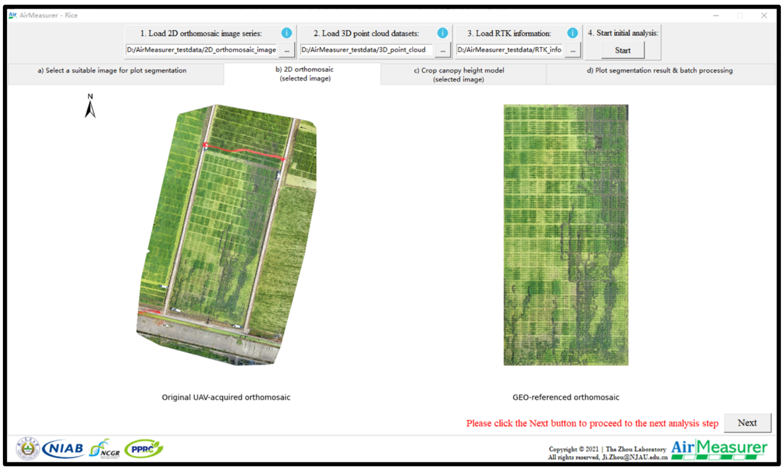

Figure S10.3 Display the original field-level orthomosaic image and the 3D- or geo-referenced orthomosaic image within the ROI

In "tab c", a grayscale CHM image (grayscale values represent height values) will be generated using the input 3D point clouds within the defined ROI. At this step, initial plot masks and plot segmentation will be displayed.

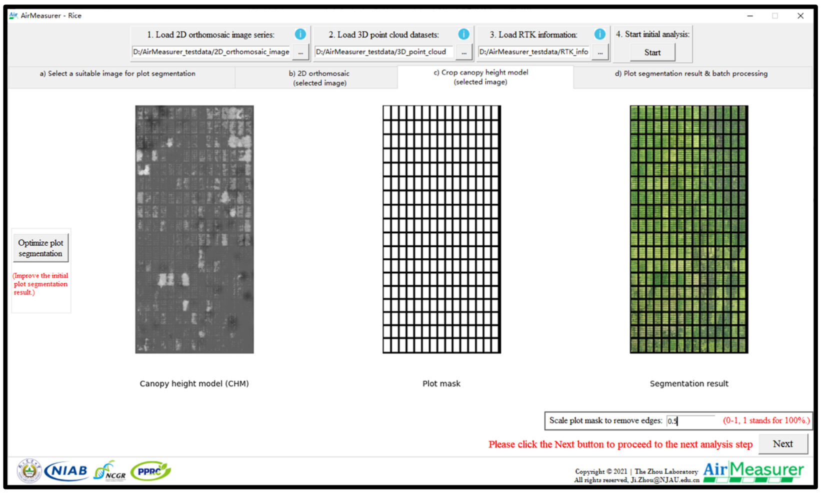

Figure S10.4 Display the CHM using 3D point clouds and initial plot segmentation results

If users are happy with the segmentation result and plot masks, they can enter a value (0-1, where 1 stands for 100% of the original masks) in the "Scale plot mask" input box (e.g. scale = 0.5; **Fig. S10.4**, right), which removes plot edges and overlapping plants through shrinking the masks.

10.3 Plot segmentation with manual optimisation

If users have selected an image to process the plot segmentation which is not satisfactory due to unclear gaps between plots. A wrongly segmented plot masks could be produced (**Fig. S10.5**, left). In this case, users can click the "Optimize plot segmentation" button in "tab c" (**Fig. S10.4**, left) to open an "Optimize mask" window to draw horizontal or vertical lines using their mouse to improve the plot delineation (**Fig. S10.5**, right). Detailed operation can be seen in **Supporting Information Video S1**.

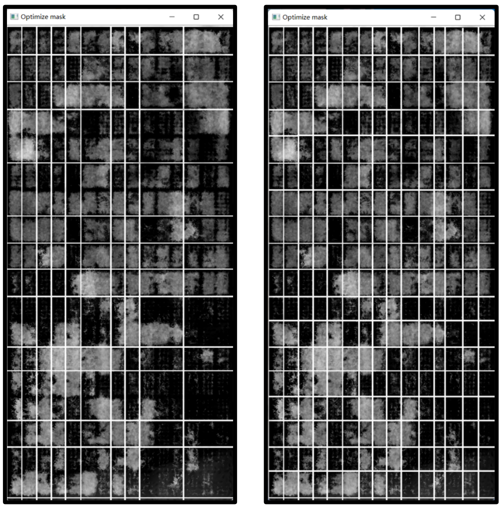

Figure S10.5 Wrongly segmented plots (left) and the refined plot segmentation after manual optimisation (right)

The Optimization button can assist users to generate base lines for the experimental field and rectify wrongly segmented plot masks under many concomitances (**Fig. S10.6**). After the optimisation, users can proceed to the next step for batch processing.

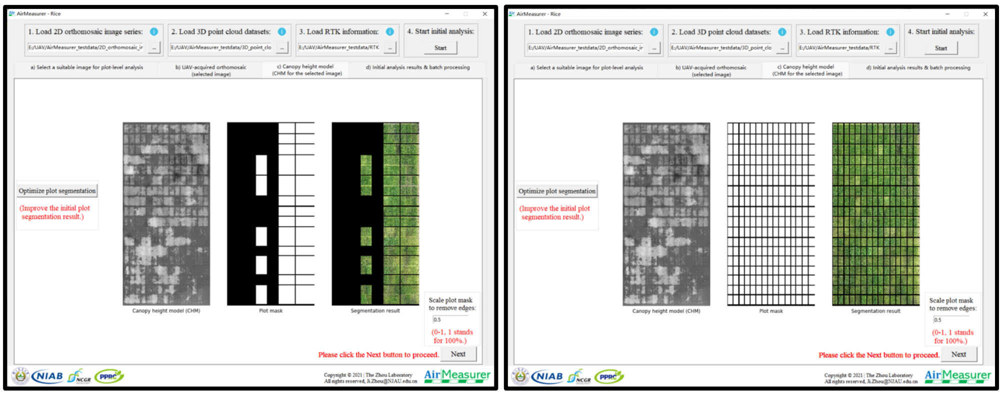

Figure S10.6 Wrongly segmented plots (left) and the improved results (right)

10.4 Batch processing

The field-level plot masks will be displayed in “tab d” together with refined sampling areas for all the plots (**Fig. S10.7**). If users are happy with the segmentation result, they can click the ‘Batch processing’ button to start the batch processing of all input 2D/3D images benchmarked with the refined plot masks.

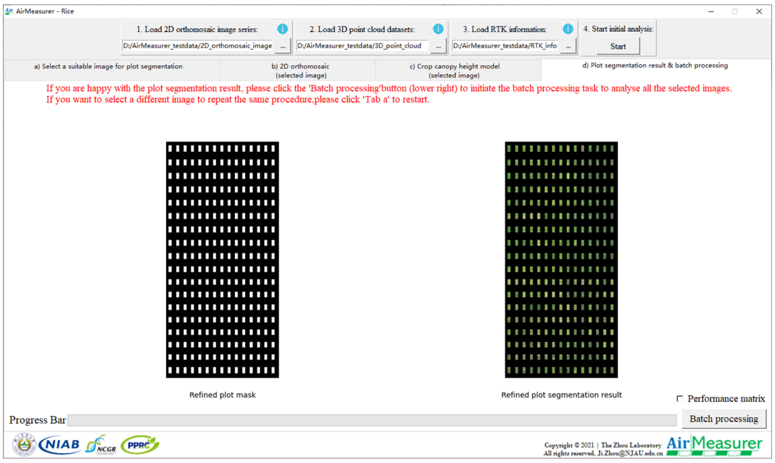

Figure S10.7 Display the result of plot segmentation before batch processing

AirMeasurer will create a Result folder named after the date and time of the input aerial imagery, which contains the batch processing results, including processed 2D/3D images (in PNG) and trait analysis (in CSV) for all the plots in the field experiment (**Fig. S10.8**). Users can also select the “Performance matrix” box (above the ‘Batch processing’ button) to generate a performance matrix to visualise plot-based phenotypic changes during the season based on all the input images (**Fig. S10.9**). At the end of the batch processing, users can download the Result folder via the GUI software.

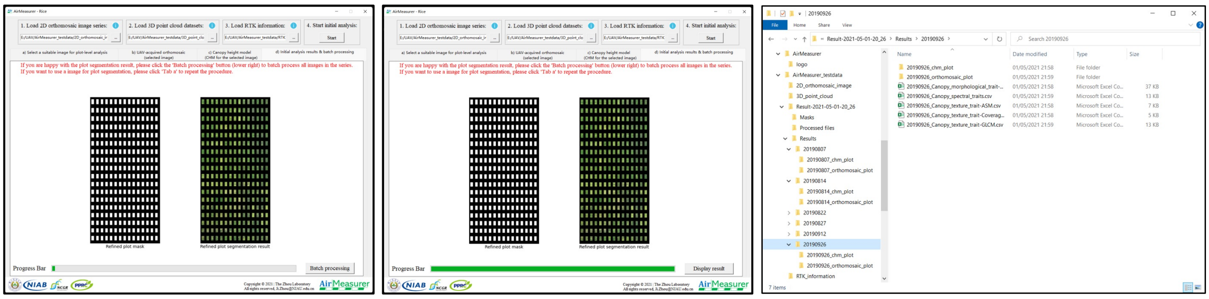

Figure S10.8 Batch processing of all the input 2D/3D UAV images and the result folder

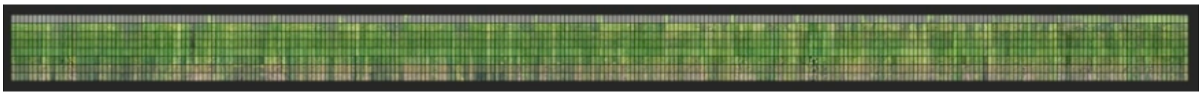

Figure S10.9 The performance matrix generated based on eight 2D/3D aerial image series

10.5 Testing AirMeasurer with different plot layouts and crops

We applied AirMeasurer to analysis wheat field experiments with varied plot layouts. The aerial imaging was conducted on wheat trials supported by the BBSRC’s Designing Future Wheat Programme

(at the John Innes Centre's Church Farm in the 2018 season; **Fig. S10.10**, upper) and the National Natural Science Foundation of China (at the Zhenjiang Agricultural Technology Innovation Center in the 2021 season; **Fig. S10.10**, lower), which produced a series of 2D orthomosaics and 3D point clouds for 240 winter wheat varieties with six-metre plots and different plot layouts.

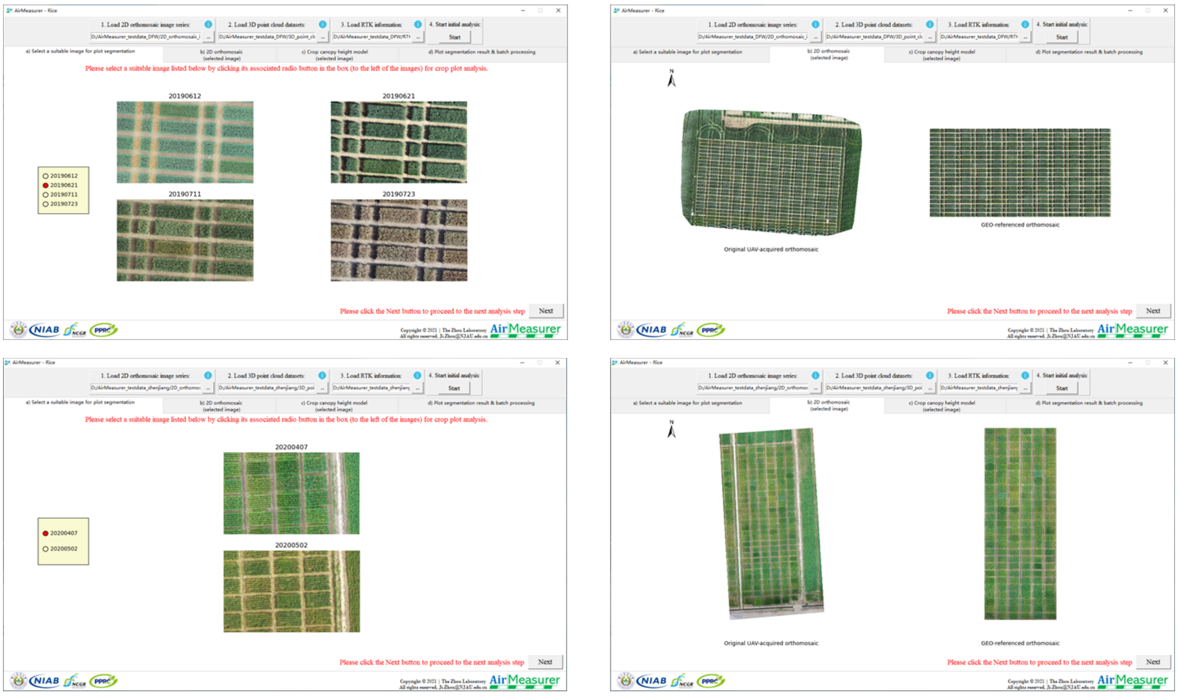

Figure S10.10 Wheat orthomosaic images processed with AirMeasurer with different plot layouts

Then, AirMeasurer was used to process the input 2D/3D wheat image series with the small 1 metre-plot removed for the JIC trials (**Fig. S10.11**; upper) and plot rescaled for the Zhenjiang trials (**Fig. S10.11**; lower) The automated trait analysis measured plot-level morphological, spectral, and textural features and produced canopy height, yield estimation (NDYI), vegetative greenness and a range of traits that are agronomically important.

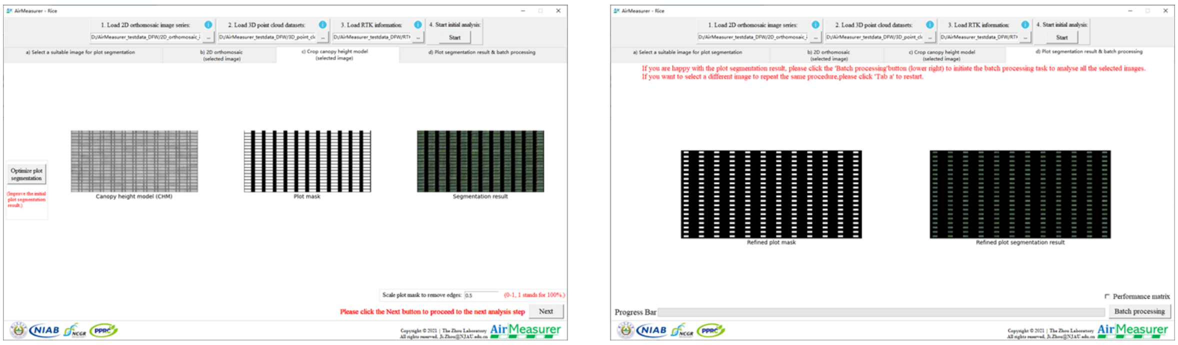

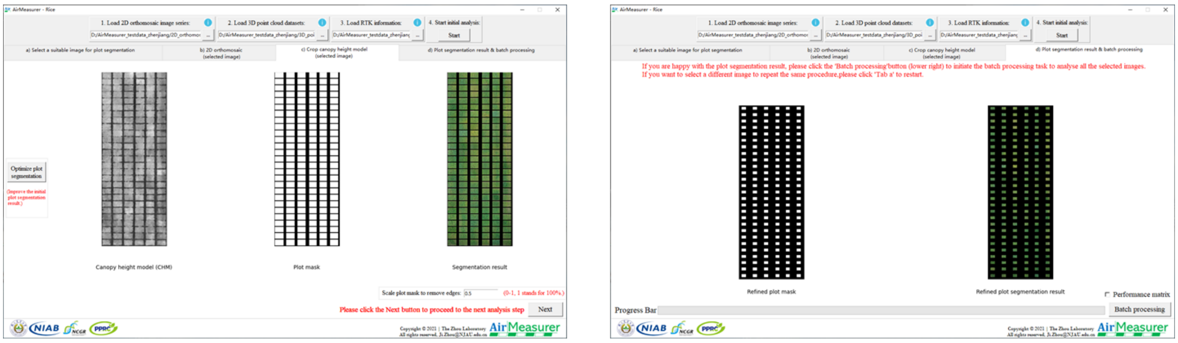

Figure S10.11 Display CHM, plot segmentation and retailed plots processed by AirMeasurer

Supporting Information Notes S11 – The entire performance matrix in the 2019 trial

A comprehensive overview of 261 rice landraces (254 varieties used for genetic mapping) performed during the 2019 season. The performance matrix used overhead plot-based canopy images to index all the varieties (**Figs. S11.1-11.4**) and rearranged the 261 landraces to three domestic groups (*indica*, *japonica* and intermediary), such that genotypes are columns and UAV measurement dates are rows. UAV-based phenotyping dates (each row in the figures below) were July 20th, Aug 7th, Aug 14th, Aug 22nd, Aug 27th, Sep 12th, Sep 26th and Oct 8th 2019. Each panel depicts 60 rice genotypes.

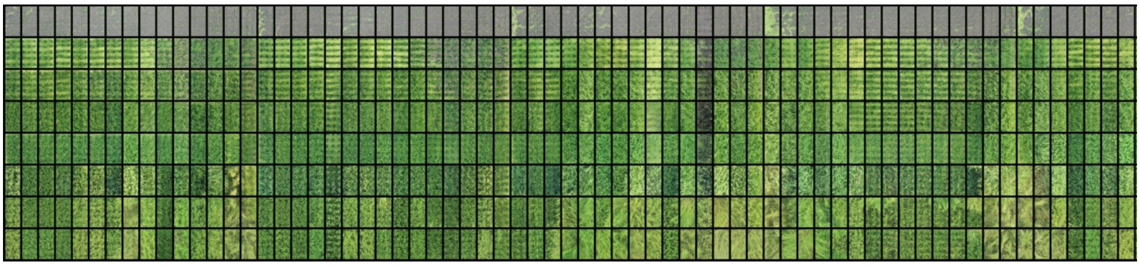

Figure S11.1 The entire performance matrix of 261 rice landraces in the 2019 season (part 1)

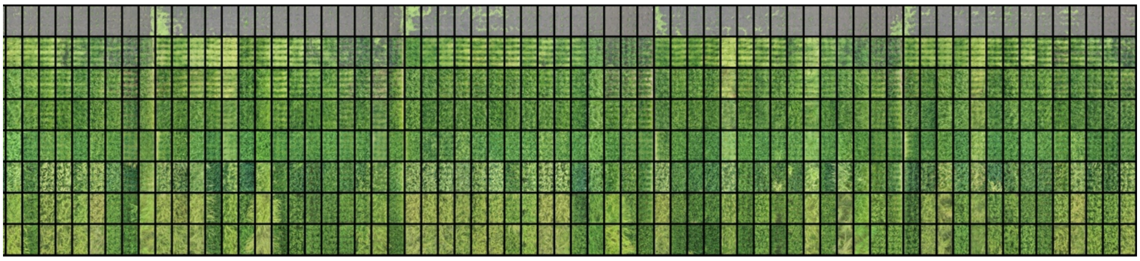

Figure S11.2 The entire performance matrix of 261 rice landraces in the 2019 season (part 2)

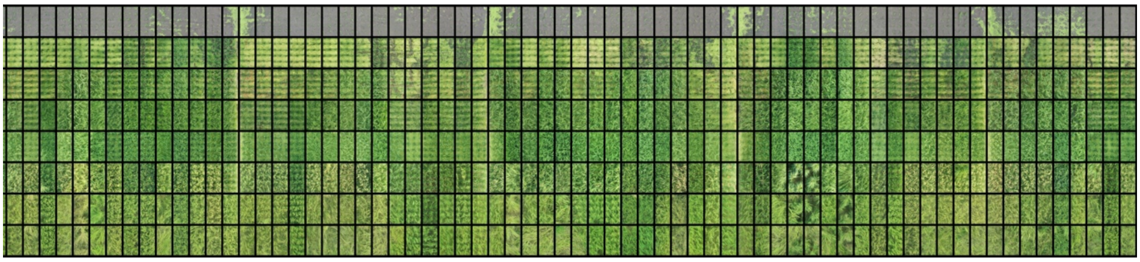

Figure S11.3 The entire performance matrix of 261 rice landraces in the 2019 season (part 3)

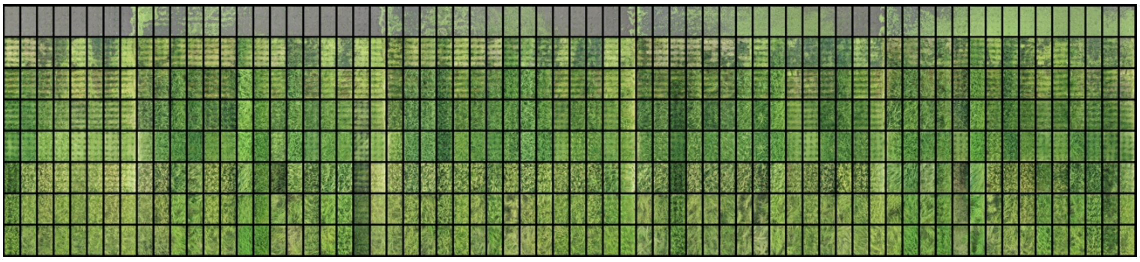

Figure S11.4 The entire performance matrix of 261 rice landraces in the 2019 season (part 4)

Supporting Information Notes S12 – Estimation of a complex trait – heading date

12.1 Modelling approach

Heading date (refers to as flowering time) is a key agronomic trait cereal breeding and cultivation. As a complex trait, the trait was estimated by field specialists through forecasting or back scoring in the field, which was laborious and prone to error (Yamamoto *et al.*, 2009). As the trait is controlled by several loci, we pioneered the prediction of this complex trait using multiple phenotypes. The modelling approach consisted: (1) *trait selection*, collating all the morphological, textural, and spectral traits measured by AirMeasurer; (2) *data pre-processing*, applying the Gaussian fitting to all the traits to compute the DAT (in days) when these traits peaked in dynamic phenotype measurement; (3) *feature engineering and selection*, engineering features to search for patterns between all the peaked traits and manually estimated heading date using an iterative loop, R^2 was used as the criteria to select traits, resulting in three traits (i.e. height, VARI and canopy coverage) identified; (4) *model training*, a range of learning techniques were tested, including XGBoost, LightGBM, multiple linear regression (MLR) and support vector regression (SVR), using a training set of 215 lines (60% for training, n = 129; 40% for testing, n = 86) with outliers removed through a residual analysis (95%, 2 SDs); amongst all the models, SVR (correlation coefficient $p = 0.849$) and MLR ($p = 0.841$) performed the best; and (5) *model validation*, both SVR and MLR were validated using the testing data, with SVR obtained a slightly higher R^2 in predicting the heading date trait ($R^2 = 0.725$, $p < 0.0001$; n = 86 plots) and MLR achieved a marginally lower correlation ($R^2 = 0.708$, $p < 0.001$; n = 86). We kept both models in GWAS analysis. The source code and explanation for the modelling approach can be seen as follows:

12.2 Feature engineering and selection

```
# Load dataset, split train/test, define cross-validation method
df = pd.read_csv('Shanghai_HeadingDate.csv')
df = df.loc[df['Not_Outlier'] == 1]
# Define target and input parameters
y = df['Manual_Heading (days)']
x = df.iloc[:, 4:7]
x_train, x_test, y_train, y_test = train_test_split(x, y, test_size=0.4, random_state=0)
# define cross-validation
kf = KFold(n_splits=5)

# Engineer features relating to key phenotypic traits to search for
```

```
# patterns between variables that correlate with heading date
x_base_features = x_train.copy()
if x_base_features: # not null
        # base feature engineering
        x_train['Height/VARI'] = x_train.iloc[:, 0] / x_train.iloc[:, 1]
        x_train['Height+VARI'] = x_train.iloc[:, 0] + x_train.iloc[:, 1]
        x_train['Height*VARI'] = x_train.iloc[:, 0] * x_train.iloc[:, 1]
        x_train['Height/Canopy'] = x_train.iloc[:, 0] / x_train.iloc[:, 2]
        x_train['Height+Canopy'] = x_train.iloc[:, 0] + x_train.iloc[:, 2]
        x_train['Height*Canopy'] = x_train.iloc[:, 0] * x_train.iloc[:, 2]
        x_train['VARI/Canopy'] = x_train.iloc[:, 1] / x_train.iloc[:, 2]
        x_train['VARI+Canopy'] = x_train.iloc[:, 1] + x_train.iloc[:, 2]
        x_train['VARI*Canopy'] = x_train.iloc[:, 1] * x_train.iloc[:, 2]
        x_train['Height_VARI_mean'] = np.mean(x_train.values[:, [0, 1]], axis=1)
        x_train['Height_Canopy_mean'] = np.mean(x_train.values[:, [0, 2]], axis=1)
        x_train['VARI_Canopy_mean'] = np.mean(x_train.values[:, [1, 2]], axis=1)
        x_train['Height_VARI_Canopy_mean']=np.mean(x_train.values[:,[0,1,2]], axis=1)
        x_test['Height/VARI'] = x_test.iloc[:, 0] / x_test.iloc[:, 1]
        x_test['Height+VARI'] = x_test.iloc[:, 0] + x_test.iloc[:, 1]
        x_test['Height*VARI'] = x_test.iloc[:, 0] * x_test.iloc[:, 1]
        x_test['Height/Canopy'] = x_test.iloc[:, 0] / x_test.iloc[:, 2]
        x_test['Height+Canopy'] = x_test.iloc[:, 0] + x_test.iloc[:, 2]
        x_test['Height*Canopy'] = x_test.iloc[:, 0] * x_test.iloc[:, 2]
        x_test['VARI/Canopy'] = x_test.iloc[:, 1] / x_test.iloc[:, 2]
        x_test['VARI+Canopy'] = x_test.iloc[:, 1] + x_test.iloc[:, 2]
        x_test['VARI*Canopy'] = x_test.iloc[:, 1] * x_test.iloc[:, 2]
        x_test['Height_VARI_mean'] = np.mean(x_test.values[:, [0, 1]], axis=1)
        x_test['Height_Canopy_mean'] = np.mean(x_test.values[:, [0, 2]], axis=1)
        x_test['VARI_Canopy_mean'] = np.mean(x_test.values[:, [1, 2]], axis=1)
        x_test['Height_VARI_Canopy_mean']=np.mean(x_test.values[:, [0,1,2]], axis=1)

# Select variables that have highest correlation/dependence with/on heading date
selector = SelectKBest(score_func=f_regression, k=7)
selector.fit(x_train, y_train)
features.append(np.argwhere(selector.get_support() == True))
idx = selector.get_support()
x_train = x_train.iloc[:, idx]
x_test = x_test.iloc[:, idx]
correlations = np.corrcoef(x_train.T, y_train)[:-1, -1]**2
base_correlations = np.corrcoef(x_base_features.T, y_train)[:-1, -1]**2
# End of engineering features, using R2 to select traits
```

12.3 MLR modelling

```
# Testing validation
# Standard error
def stdError_func(y_test, y):
  return np.sqrt(np.mean((y_test - y) ** 2))
# RMSE, root mean square error
def R2_1_func(y_test, y):
  return 1 - ((y_test - y) ** 2).sum() / ((y.mean() - y) ** 2).sum()
# R2 correlation
```

```
def R2_2_func(y_test, y):
  y_mean = np.array(y)
  y_mean[:] = y.mean()
  return 1 - stdError_func(y_test, y) / stdError_func(y_mean, y)

# Training the MLR-based model
# Use the data file defined in section 8.1
x = np.array(df.iloc[:,4:7].values)
y = np.array(df.iloc[:,3:4].values)
cft = linear_model.LinearRegression()
cft.fit(x, y)
print("model coefficients", cft.coef_)
print("model intercept", cft.intercept_)

# Model evaluation
predict_y =  cft.predict(x)
strError = stdError_func(predict_y, y)
R2_1 = R2_1_func(predict_y, y)
R2_2 = R2_2_func(predict_y, y)
score = cft.score(x, y)
print('strError={:.2f}, R2_1={:.2f},  R2_2={:.2f}, clf.score={:.2f}'.format(
    strError,R2_1,R2_2,score))

# End of model training and testing
```

```
model coefficients [[0.60995154 0.76321628 0.27883231]]
model intercept [-55.68898622]
strError=5.96, R2_1=0.74,  R2_2=0.49, clf.score=0.74
```

We applied the multiple regression model to forecast the heading date trait using the testing data. We obtained an R^2 value 0.74 ($p < 0.0001$; $n = 86$ plots) between the prediction and the manual estimation. Using the training data to train the learning model and computed coefficients and intercept of the multiple regression (**Eqn S1.**):

$$HeadingDate(i) = 0.610 * H(i) + 0.763 * V(i) + 0.279 * C(i) - 55.689 \qquad \text{(S1)}$$

Where *H* is the number of days between the sowing date and the date when crop height reached the peak value, *V* is the number of days between the sowing date and the date when VARI reached the peak value, *C* is the number of days between the sowing date and the date when canopy coverage reached the peak value, and *i* is the variety monitored during the season.

12.4 SVR modelling

```
# Prepare arrays to train, test and validate the SVR model
preds = []
valid_target = []
valid_preds = []
```

```
# Predict using regression SVM model
for train_index, test_index in kf.split(x_train):
    reg = SVR(C=2500, epsilon=2)
    X_train, X_valid = x_train.iloc[train_index], x_train.iloc[test_index]
    Y_train, Y_valid = y_train.iloc[train_index], y_train.iloc[test_index]
    reg.fit(X_train, Y_train)
    preds.append(reg.predict(x_test))
    valid_preds.append(reg.predict(X_valid))
    valid_target.append(Y_valid)

preds = np.mean(preds, axis=0)
valid_preds = np.concatenate(valid_preds)
valid_target = np.concatenate(valid_target)

# Print metrics such as mean squared error and R2
print("Validation MSE", mean_squared_error(valid_preds, valid_target))
corr = np.corrcoef(valid_preds, valid_target)
print("Validation correlation", corr[0, 1]**2)
corr = np.corrcoef(preds, y_test)
print("Test MSE", mean_squared_error(preds, y_test))
print("Test correlation", corr[0, 1]**2)
# End of model training and testing
```

```
Validation MSE 42.3401686670458
Validation correlation 0.724363929415012
Test MSE 40.802470734598245
Test correlation 0.7254510686615001
```

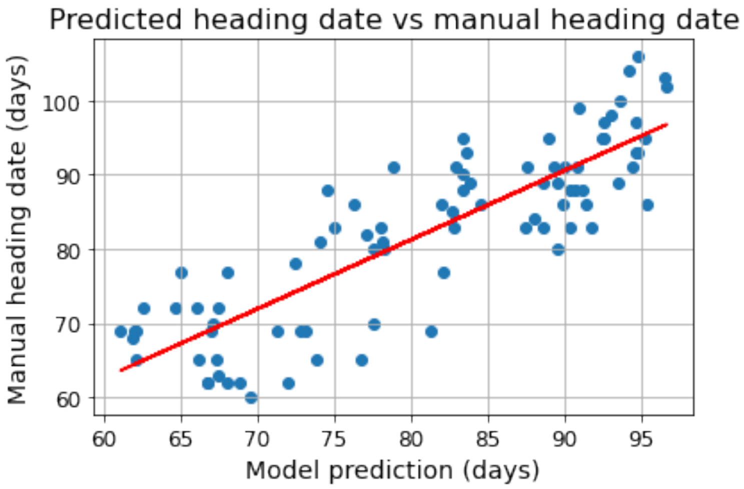

Figure S12.1 Textural traits measured based on canopy-level CHM images

Supporting Information Notes S13 – GWAS using heading dates estimated by the SVR model

13.1 GWAS using estimated heading date with indica

We first chose 97 *indica* landraces to perform GWAS using the heading date trait estimated by the MRL model, with the *P* value threshold was set as 1.7×10^{-6} (FDR of 0.2). Using the threshold, we found a SNP associated with the trait (**Supporting Information Table S13.1**; **Fig. S13.1**), suggesting the strongest signal ($-\log_{10}(P) = 5.79$) was just ~17.14kb away from the *OsSOC1* gene on chromosome 3. Besides the significant SNP, we also found a very strong signal ($-\log_{10}(P) = 4.65$) was ~30.24kb away from the *Hd3a* gene located on chromosome 6.

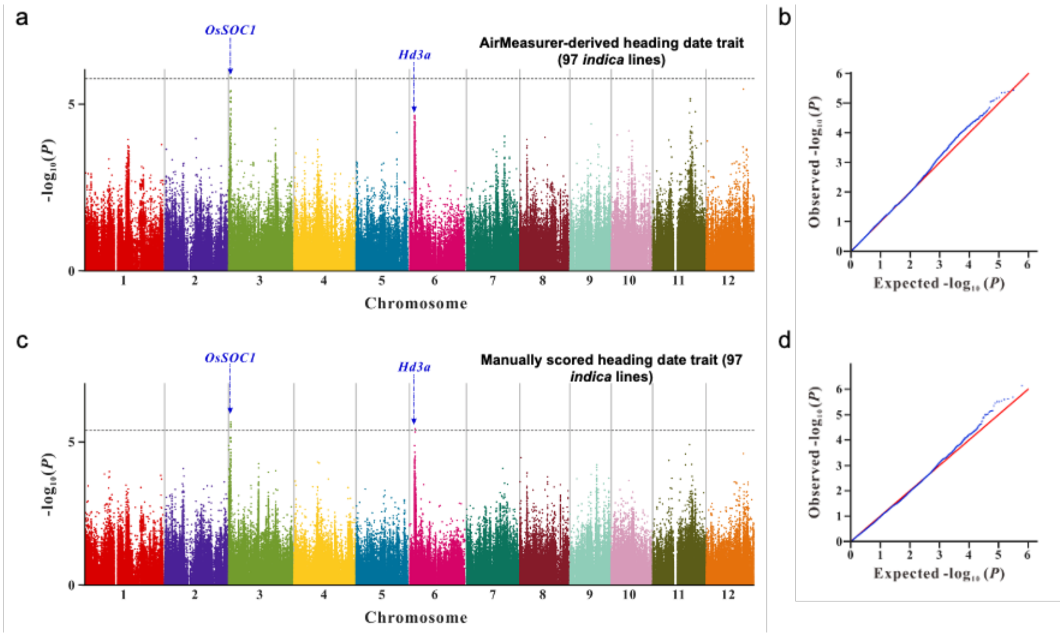

Figure S13.1 The GWAS analysis of the heading date trait using 97 *indica* rice landraces through EMMAx. For the significant loci identified, known loci were pointed out by blue arrows.

Supporting Information table S13.1. Genome-wide significant association signals of traits using EMMAx.

Trait	Chr.	Position[a]	$-\log_{10}P$	Candidate genes
Heading date estimated by manual scoring	3	1,295,758	6.14	*OsSOC1*
	6	3,287,878	5.46	*Hd3a*
Heading date estimated by AirMeasurer's modelling	3	1,295,758	5.79	*OsSOC1*

[a]Position in bp according to IRGSP 4.0

13.2 GWAS using estimated heading date with all landraces

By performing GWAS using the heading date trait estimated by the SVR model for the 114 *indica* landraces with a total of 100 permutation tests. We found a SNP associated with the heading trait estimated by the SVR model. The Manhattan plot and the threshold (blue dotted line; **Fig. S13.2a**) suggested that the strongest signal (with -log$_{10}$(P) = 6, indicated with a blue arrow) was ~17.14kb away from the *OsSOC1* gene on chromosome 3. Besides the significant SNP over the threshold, we also found a strong signal (-log$_{10}$(P) = 5.16) that was ~149.01kb away from the *Hd3a* gene located on chromosome 6. Similarly, 100 permutation tests were performed to determine the significant *P* value threshold for 104 *japonica* landraces. The GWAS results suggested that the strongest signal was ~346.05kb away from the *RCN2b* gene on chromosome 2 (**Fig. S13.2b**), which was reported to control the progression of rice developmental stages (Nakagawa *et al.*, 2002). When applying GWAS to all 259 rice landraces, only one significant SNP was identified, which was ~361.71kb away from the *RCN2* gene (**Fig. S13.2c**).

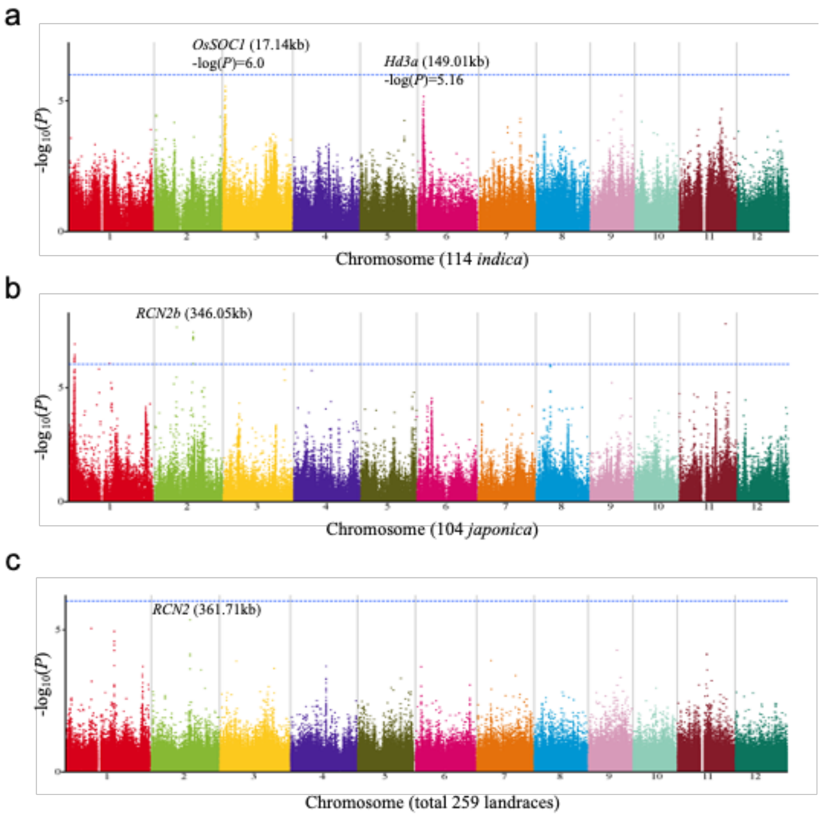

Figure S13.2 The GWAS analysis of heading date with 114 *indica* landraces using EMMAx

Supporting Information Notes S14 – Applying AirMeasurer to examine wheat plots under different nitrogen treatments

We applied AirMeasurer and the imaging protocol in wheat experiments to study traits such as plant height, yield and vegetative greenness using 54 wheat varieties (Zhu *et al.*, 2021). Following the aerial imaging, collected image series (**Fig. S14.1a**) were first pre-processing to generate 2D orthomosaics and 3D point clouds (**Fig. S14.1b**), followed by automated analysis to measure plot-level morphological, spectral, and textural features to study canopy height, yield estimation (NDYI) and vegetative greenness.

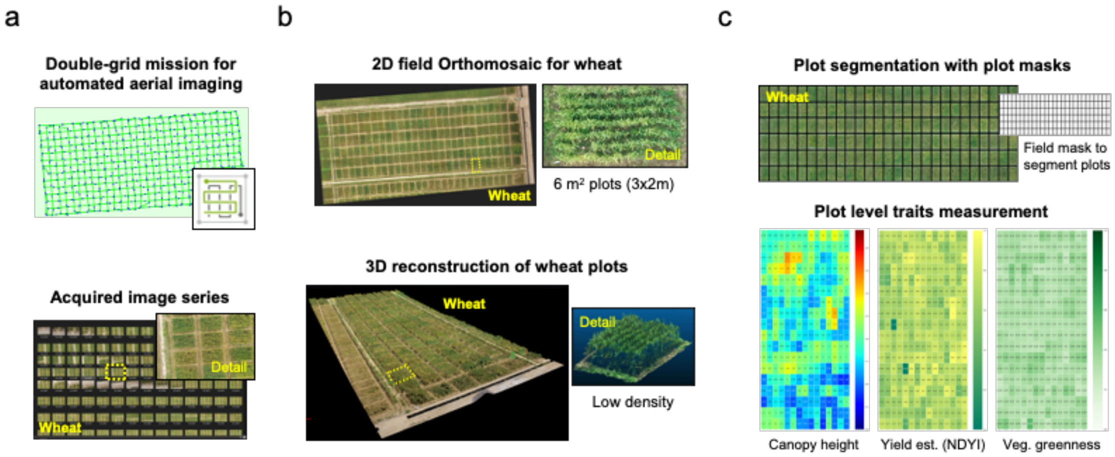

Figure S14.1 Apply the aerial imaging protocol and AirMeasurer to wheat field experiments

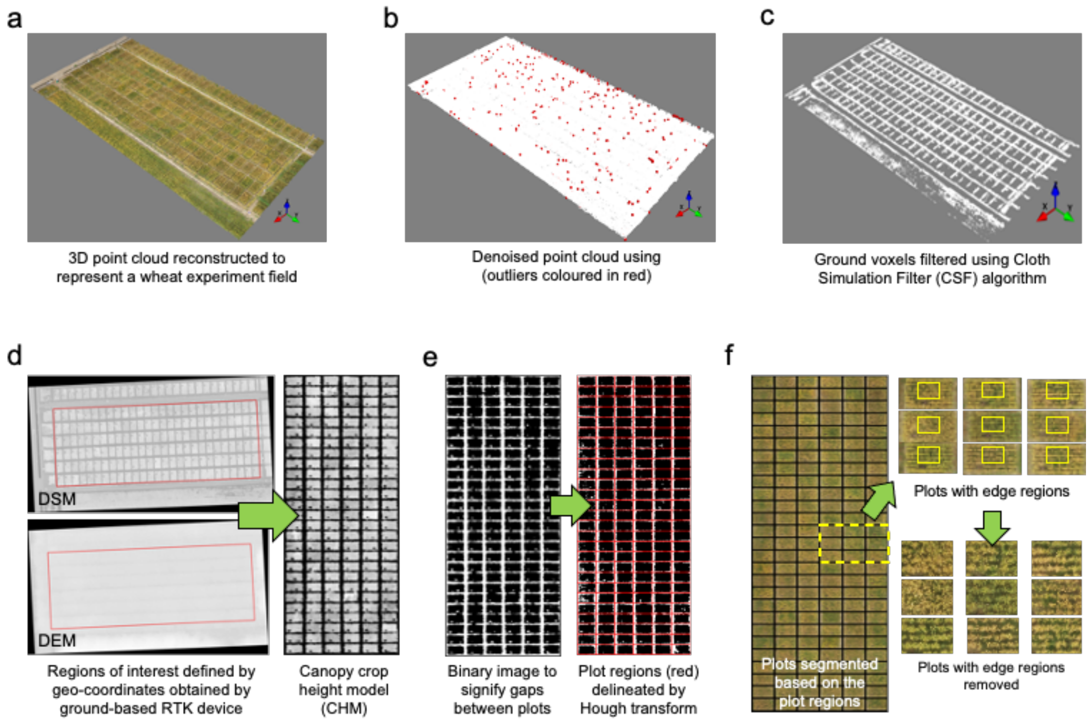

Figure S14.2 The analysis workflow of AirMeasurer for processing wheat traits

Similar to the analysis workflow for rice, we selected a LAS point cloud file and removed outliers (coloured red) using the SOR algorithm, followed by the application of the CSF to differentiate ground-based and above-ground 3D points (**Figs. S14.2a-c**). Then, we produced generate a 2D CHM and employed the plot segmentation algorithm in AirMeasurer to divide wheat plots, based on which plot-level traits were analysed within refined sampling areas (**Figs. S14.2d-f**).

Support Information Video

Video S1. The video shows how to use the GUI of AirMeasurer (in exe format) in operation, which includes loading datasets, displaying 2D orthomosaics, selecting an image for pre-processing, defining regions of interest (ROIs) using the input SHP file, generating CHM based on calibrated 3D point clouds, performing automated plot segmentation (if needed, improving the plot segmentation results with human optimisation), accepting initial analysis with scaled plot masks, carrying out batch processing for all the input 2D/3D aerial imagery, and producing the performance matrix and plot-based trait analysis results.

References

Anderson SL, Murray SC. **2020**. R/UAStools::plotshpcreate: Create Multi-Polygon Shapefiles for Extraction of Research Plot Scale Agriculture Remote Sensing Data. *Frontiers in Plant Science* **11**: 1–7.

Bauer A, Bostrom AG, Ball J, Applegate C, Cheng T, Laycock S, Rojas SM, Kirwan J, Zhou J. **2019**. Combining computer vision and deep learning to enable ultra-scale aerial phenotyping and precision agriculture: A case study of lettuce production. *Horticulture Research* **6**: 1–12.

Chen CJ, Zhang Z. **2020**. GRID: A python package for field plot phenotyping using aerial images. *Remote Sensing* **12**: 1697.

Gitelson AA, Stark R, Rundquist D, Gitelson AA, Kaufman YJ, Stark R, Rundquist D. **2002**. Novel algorithms for remote estimation of vegetation fraction. *Remote sensing of Environment* **80**: 76–87.

Holman FH, Riche AB, Castle M, Wooster MJ, Hawkesford MJ. **2019**. Radiometric Calibration of 'Commercial off the Shelf' Cameras for UAV-Based High-Resolution Temporal Crop Phenotyping

of Reflectance and NDVI. *Remote Sensing* **11**: 1657.

Huang X, Wei X, Sang T, Zhao Q, Feng Q, Zhao Y, Li C, Zhu C, Lu T, Zhang Z, *et al.* 2010. Genome-wide association studies of 14 agronomic traits in rice landraces. *Nature genetics* **42**: 961–967.

Huang X, Zhao Y, Wei X, Li C, Wang A, Zhao Q, Li W, Guo Y, Deng L, Zhu C, *et al.* 2012. Genome-wide association study of flowering time and grain yield traits in a worldwide collection of rice germplasm. *Nature Genetics* **44**: 32–39.

Liu L, Zhao L, Long Y, Kuang G, Fieguth P. **2012**. Extended local binary patterns for texture classification. *Image and Vision Computing* **30**: 86–99.

Matias FI, Caraza-Harter M V., Endelman JB. **2020**. FIELDimageR: An R package to analyze orthomosaic images from agricultural field trials. *Plant Phenome Journal* **3**: 1–6.

Meyer GE, Neto JC. **2008**. Verification of color vegetation indices for automated crop imaging applications. *Computers and Electronics in Agriculture* **63**: 282–293.

Nakagawa M, Shimamoto K, Kyozuka J. **2002**. Overexpression of RCN1 and RCN2, rice Terminal Flower 1/Centroradialis homologs, confers delay of phase transition and altered panicle morphology in rice. *Plant Journal* **29**: 743–750.

Öztürk Ş, Akdemir B. **2018**. Application of Feature Extraction and Classification Methods for Histopathological Image using GLCM, LBP, LBGLCM, GLRLM and SFTA. *Procedia Computer Science* **132**: 40–46.

Sulik JJ, Long DS. **2016**. Spectral considerations for modeling yield of canola. *Remote Sensing of Environment* **184**: 161–174.

Sun G, Wang X, Yang H, Zhang X. **2020**. A canopy information measurement method for modern standardized apple orchards based on UAV multimodal information. *Sensors (Switzerland)* **20**: 2985.

Tresch L, Mu Y, Itoh A, Kaga A, Taguchi K, Hirafuji M, Ninomiya S, Guo W. **2019**. Easy MPE: Extraction of quality microplot images for UAV-based high-throughput field phenotyping. *Plant Phenomics* **2019**: 1–9.

Xue J, Su B. **2017**. Significant remote sensing vegetation indices: A review of developments and applications. *Journal of Sensors* **2017**: 1353691.

Yamamoto T, Yonemaru J, Yano M. **2009**. Towards the understanding of complex traits in rice: Substantially or superficially? *DNA Research* **16**: 141–154.

Zhang T, Tang H. **2018**. A Comprehensive evaluation of approaches for built-up area extraction from Landsat OLI images using massive samples. *Remote Sensing* **11**: 1–24.

Zhu Y, Sun G, Ding G, Zhou J, Wen M, Jin S, Zhao Q, Colmer J, Ding Y, Ober ES, *et al.* **2021**. Large-scale field phenotyping using backpack LiDAR and CropQuant-3D to measure structural variation in wheat. *Plant Physiology* **187**: 716–738.
